# Recovering the precolonial population structure of Khoe-San descendant populations

**DOI:** 10.1126/sciadv.adz7115

**Published:** 2026-09-02

**Authors:** Stacy L. Edington, Dana R. Al-Hindi, Neus Font-Porterias, Alexandra Surowiec, William J. Palmer, Justin W. Myrick, Paul J. Norman, Caitlin Uren, Marlo Möller, Brenna M. Henn, Austin W. Reynolds

**Affiliations:** ^1^ Department of Biology, Baylor University, Waco, TX, USA.; ^2^ Department of Anthropology, University of California Davis, Davis, CA, USA.; ^3^ Department of Biomedical Informatics, University of Colorado School of Medicine, Aurora, CO, USA.; ^4^ South African Medical Research Council Centre for Tuberculosis Research, Division of Molecular Biology and Human Genetics, Faculty of Medicine and Health Sciences, Stellenbosch University, Cape Town, South Africa.; ^5^ Centre for Bioinformatics and Computational Biology, Stellenbosch University, Stellenbosch, South Africa.; ^6^ National Institute for Theoretical and Computational Sciences (NITheCS), Stellenbosch University, Stellenbosch, South Africa.; ^7^ Genomics for Health in Africa (GHA), Africa-Europe Cluster of Research Excellence (CoRE), University of Ghana, Legon, Ghana.; ^8^ UC Davis Genome Center, University of California Davis, Davis, CA, USA.; ^9^ Department of Microbiology, Immunology, and Genetics, University of North Texas Health Science Center at Fort Worth, Fort Worth, TX, USA.

## Abstract

San populations from Botswana and Namibia retain exceptional linguistic, cultural, and genetic diversity, but few Khoisan-speaking groups remain south of the Kalahari Desert. However, historically, far southern Africa was home to many San and Khoekhoe groups. Popular opinion often implies that such populations do not contribute to the ancestry of contemporary South Africans. Here, we characterize the genetic ancestry of self-identified South African Coloured groups and reconstruct precolonial and colonial population structures from 620 newly sampled individuals. These groups retain the majority of Khoe-San genetic ancestry (>48%), suggesting the persistence of Khoe-San ancestry to the present day. By isolating the Khoe-San ancestry component, we show that it is intermediate between the ≠Khomani San and Nama and distinct from Kalahari Khoe-San populations. We also find that signatures of the Indian Ocean slave trade can be traced to Indonesian islands such as Sulawesi, Java, and Flores, while the South Asian ancestry is regionally nonspecific.

## INTRODUCTION

Present-day populations in South Africa reflect diverse origins, from the Indigenous San and Khoe people to migrants from varying regions throughout Africa, Europe, and Asia. The region’s ethnic mosaic results from a series of complex migrations over the past 2 thousand years (kyr). The earliest migrations of pastoralist groups from eastern Africa into the region and later influxes of agropastoralist groups of western African ancestry have been well documented in both the archaeological and genetics literature (*1*, *2*). However, the impacts of more recent population movements during the colonial period have been less well studied from a genetic perspective.

The arrival of the Dutch East India Company (in Dutch: *Vereenigde Nederlandsche Geoctroyeerde Oostindische Compagnie* or *“*VOC”) in 1652 initiated South Africa’s colonial period in Table Bay, situated near modern day Cape Town (*3*). At the time, the Company intended for the Cape to act as a refreshment post for ships traveling between Europe and Asia, as its primary colonies were situated in Sri Lanka and Jakarta—historically known as Ceylon and Batavia (*4*). However, by 1690, the resupply station had grown into a permanent Dutch colony. The VOC maintained its occupation of the Cape for nearly 200 years, its rule ending when the British seized control of the region in 1806 (*4*). During this period, the VOC is estimated to have imported upward of 63,000 to 80,000 enslaved individuals from eastern coastal Africa, Madagascar, India, Sri Lanka, and the Malay Archipelago (*3*, *4*). While historians have reconstructed the port of origin for many enslaved individuals, substantial gaps remain in our understanding of how they contributed to the genetic diversity of contemporary South Africans.

**Fig. 1.**
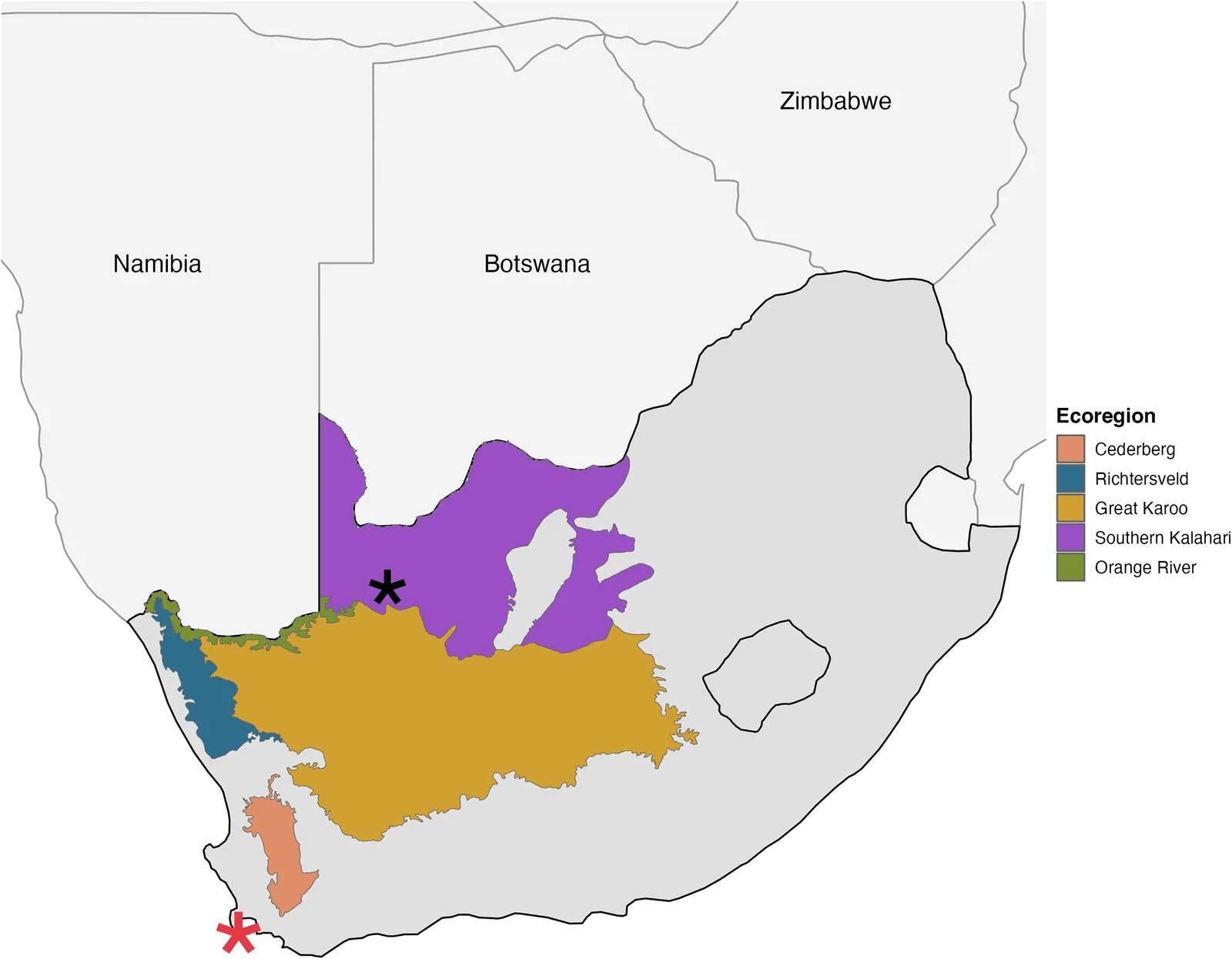
Map of South Africa highlighting the sampling regions included in this study. Highlighted areas indicate the geographical locations where participants were recruited: CDB (*n* = 204), Richtersveld (*n* = 206), KRO (*n* = 176), southern Kalahari (*n* = 310), and the ORG (*n* = 101). The total sample size across all regions was 997 individuals, including the 620 newly reported here. The red star indicates Cape Town, and the black star indicates Upington.

Over the next several decades, European colonists continued their expansion past Cape Town, reaching the Cederberg (CDB) mountains to the northwest by the 1720s, extending further north into the Richtersveld and southern Kalahari in the early 19th century, and advancing into the arid inland Karoo (KRO) by the 1840s (Fig. 1) (*5*). Their incursion resulted in population decline and the systematic displacement of the San and Khoe Indigenous groups (*6*, *7*). In some cases, San and Khoe individuals (hereafter collectively referred to as Khoe-San, reflecting their shared genetic ancestry) were incorporated into colonial society, forming new genetically heterogeneous communities with VOC-forced migrants and colonists, later subsumed under the term Coloured. This was a preapartheid term historically imposed to categorize people of mixed ancestry, that is, individuals who did not fit within the constructed White or Bantu racial categories (*3*). The term is still in use today and a formally recognized ethnic classification in South Africa, although we acknowledge that it may carry derogatory connotations in some contexts.

While this history is well established in academic contexts, South African Coloured (SAC) communities today have little formal exposure to this history beyond the initial founding of the VOC colony. Whether SAC communities outside of Cape Town have Khoe-San ancestry is often unclear unless individuals retain an oral history for their family. SAC individuals make up ∼41% of the population of the Northern and Western Cape Provinces (*8*) and are frequently described as five-way admixed (*9*, *10*), composed of Khoe-San, European, non–Khoe-San African (NKA), South Asian, and other Southeast Asian ancestries. While the global genetic ancestry of these communities along the southern coast has been characterized (*11*–*14*), the temporal dynamics of admixture and the relative genetic contributions of different ancestral groups of SAC communities further from Cape Town remain largely unexplored.

We address these gaps in knowledge by analyzing 620 newly genotyped samples from self-identified SAC and Khoe-San individuals from the Western and Northern Cape Provinces, along with 22 reference populations that serve as putative sources of genome-wide ancestry from regions proximal to VOC operation sites and trade routes. These include populations from the Coromandel and Bengal coasts and across the Indonesian archipelago. Using this new robust reference panel, we investigate the genomic impacts of colonial dynamics in these regions and assess putative source populations for each of the five major distinct ancestries previously found among the SAC populations: Khoe-San, Bantu/NKA, South Asian, European, and East/Southeast Asian.

In particular, we focus on the Khoe-San ancestry found in these communities. Compared to other global populations, the Khoe-San exhibit the highest levels of genetic heterogeneity, retain more ancestral alleles, and harbor autochthonous Y-chromosome and mitochondrial DNA haplotypes (*15*–*19*). Previous studies focused on the Khoe-San substructure for groups in and around the Kalahari Desert, estimated to have diverged ∼20 to 30 thousand years ago (kya) (*20*, *21*). The Botswanan San–Tuu–speaking !Xoo and Kx’a-speaking Ju|’hoansi harbor mostly northern Kalahari Khoe-San ancestry, while the South African Tuu–speaking ≠Khomani San and Khoekhoe pastoralists Nama retain the majority of southern Kalahari ancestry. Models including geographic location, language, and subsistence strategies improved the predictive power of these genetic components than geography alone, suggesting that all of these factors contribute to the genetic substructure within the Khoe-San (*21*). However, the genetic affinity of Khoe-San people south of the Kalahari Desert, including our study region, remains largely unknown, despite this region historically being home to thousands of individuals (*22*). We characterize the precolonial population structure and haplotype sharing of these Khoe-San descendant individuals by isolating the Khoe-San ancestry haplotypes and assessing their genetic similarities relative to four Khoe-San reference populations: ≠Khomani San, Nama, !Xoo, and Ju|’hoansi.

Last, we estimate the proportion of Khoe-San ancestry that persists across more than a dozen contemporary communities on the basis of sampled individuals from the CDB, KRO, Middle Orange River (ORG), and Upington regions and investigate how geographic and environmental factors influence gene flow between these communities. Previous efforts to characterize the Khoe-San ancestry among SAC populations were more heavily focused on the Western Cape (*11*, *23*) or were limited by small sample sizes from the Northern Cape Province [∼50 total (*14*, *21*, *24*)], potentially missing representation for the broader population.

## RESULTS

### Genetic composition of Khoe-San descendant communities

**Fig. 2.**
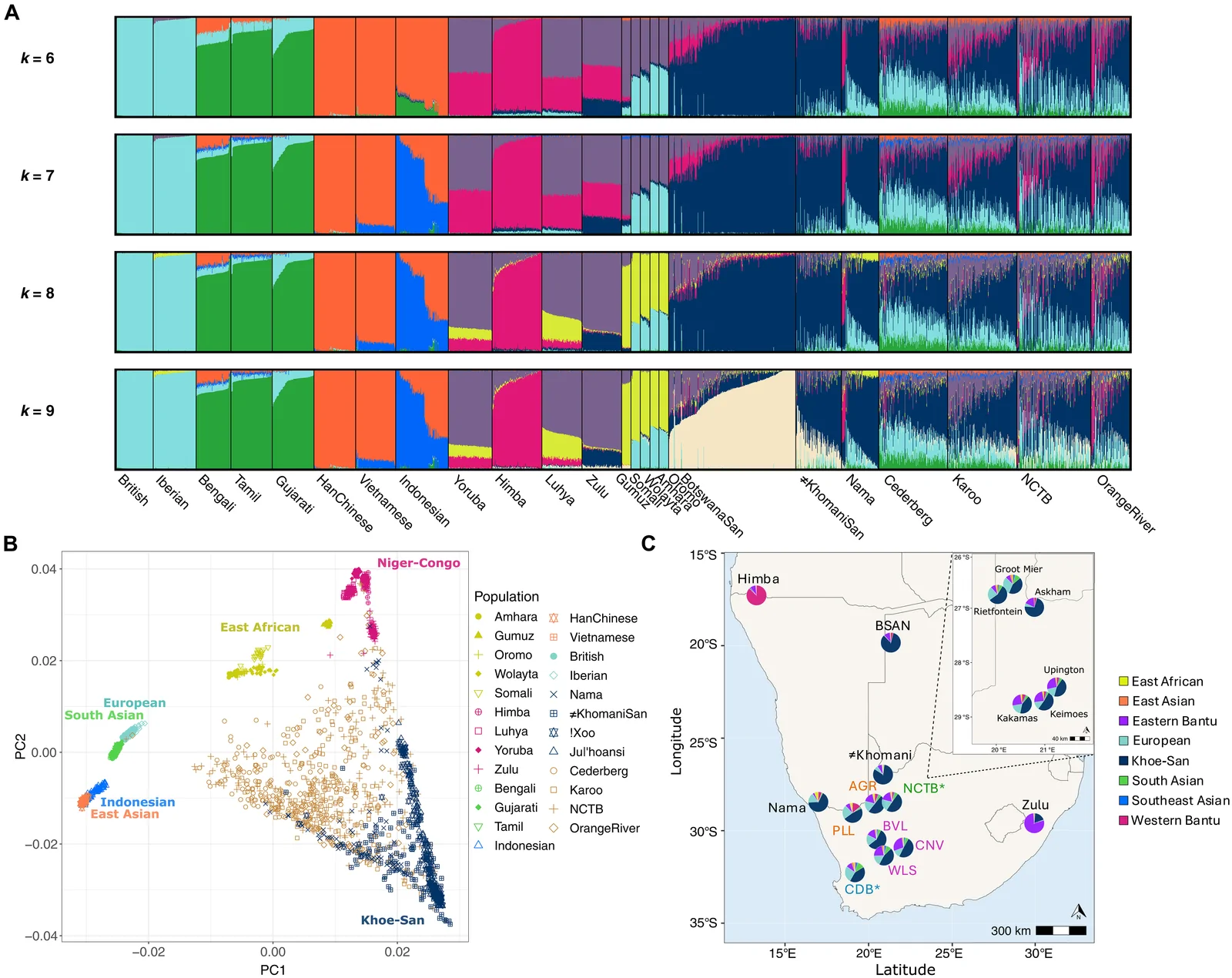
Characterizing the global population structure within newly collected samples. Colors throughout the figures corresponding to broadly inferred ancestry from ADMIXTURE *k* = 8. Population labels and abbreviations correspond the respective genome project (1000GP, African Genome Variation Project, and HGDP), with the exception of Indonesians (IND). In some instances, the !Xoo and Ju|’hoansi, who were inferred on the basis of a series of analyses (see Supplementary Text), are merged into one population: Botswanan San (BSAN). (**A**) Unsupervised ADMIXTURE run of *k* = 6 through *k* = 9. (**B**) PCA on 806,460 congruent H3Africa array markers. Visualized are 1165 individuals along PC1 and PC2, with shapes distinguishing populations within broader inferred ancestries (*k* = 8). Study populations are colored beige, while shapes indicate sampling locality within South Africa. (**C**) Approximate locations and ADMIXTURE *k* = 8 results of southern African populations included in the study. We report ADMIXTURE *k* = 8 results in the map to highlight differences in West and East southern Bantu components among the South African cohort. Newly collected samples are segregated and colored within sampling regions, with the exception of the CDB in blue and the NCTB project in green. Towns sampled across the CDB are to close in proximity to segregate, while the inset map depicts sampling locations for the NCTB project. ORG (labeled in orange) samples were collected from Pella (PLL) and Augrabies (AGR), while KRO (purple) samples were collected across three towns: Williston (WLS), Brandvlei (BVL), and Carnarvon (CNV).

We characterize the genetic composition of the largest set of SAC samples (*n* = 997) derived from outside of Cape Town, including newly collected samples spanning four southern African regions {CDB, KRO, Southern Kalahari [Upington area; Northern Cape Tuberculosis (NCTB)], and Middle ORG; Figs. 1 and 2C}. Newly collected southern African samples were assayed for >2.2 million single-nucleotide polymorphisms (SNPs) on Illumina’s H3Africa array and then merged with a reference panel composed of 1165 individuals from inter- and intracontinental populations (see the “Sample consolidation, genotyping, and PCA” section). Building upon previous studies, we assembled a comprehensive reference panel that aims to capture the greater depth and fine-scale genetic diversity of southern African populations. The reference panel was also assembled with the goal of historically contextualizing the various migrations into southern Africa (*11*, *13*, *14*, *24*). Here, we include populations that represent six broad genetic ancestries: Niger-Congo, Khoe-San, European, South, Southeast (Indonesian), and East Asian ancestries, with >120 samples representing each ancestry (Fig. 2, A and B). Niger-Congo languages are the most commonly spoken language families spoken across sub-Saharan Africa as a result of the Bantu expansion. Populations included in this study and those whose broader ancestry is labeled Niger-Congo reside in western, eastern, and southern Africa but can largely trace back their genealogies to western Africa. We include populations to capture western and eastern African Bantu expansion streams (*25*, *26*), five East African populations, 130 Indonesian genomes, and more than 500 individuals representing four Khoe and San populations to capture both northern and southern Kalahari genetic signals, with the Ju|’hoansi and !Xoo collectively labeled as Botswanan San (table S1). The final merged dataset was composed of 806,460 autosomal markers.

Following quality control filtering (see Methods), newly collected samples were assessed using principal components analysis (PCA) (*27*). Principal component 1 (PC1) segregates populations out of Africa from African populations, while PC2 separates within-Africa populations, mainly the southern African Khoe-San groups from Niger-Congo–speaking populations originating from western/central Africa (Fig. 2B). Most of KRO, CDB, Upington area (NCTB), and Middle ORG communities are distributed across the center of PC1 and PC2. Similar distribution of reference population and sampled SAC communities has previously been observed (*13*, *14*).

**Fig. 3.**
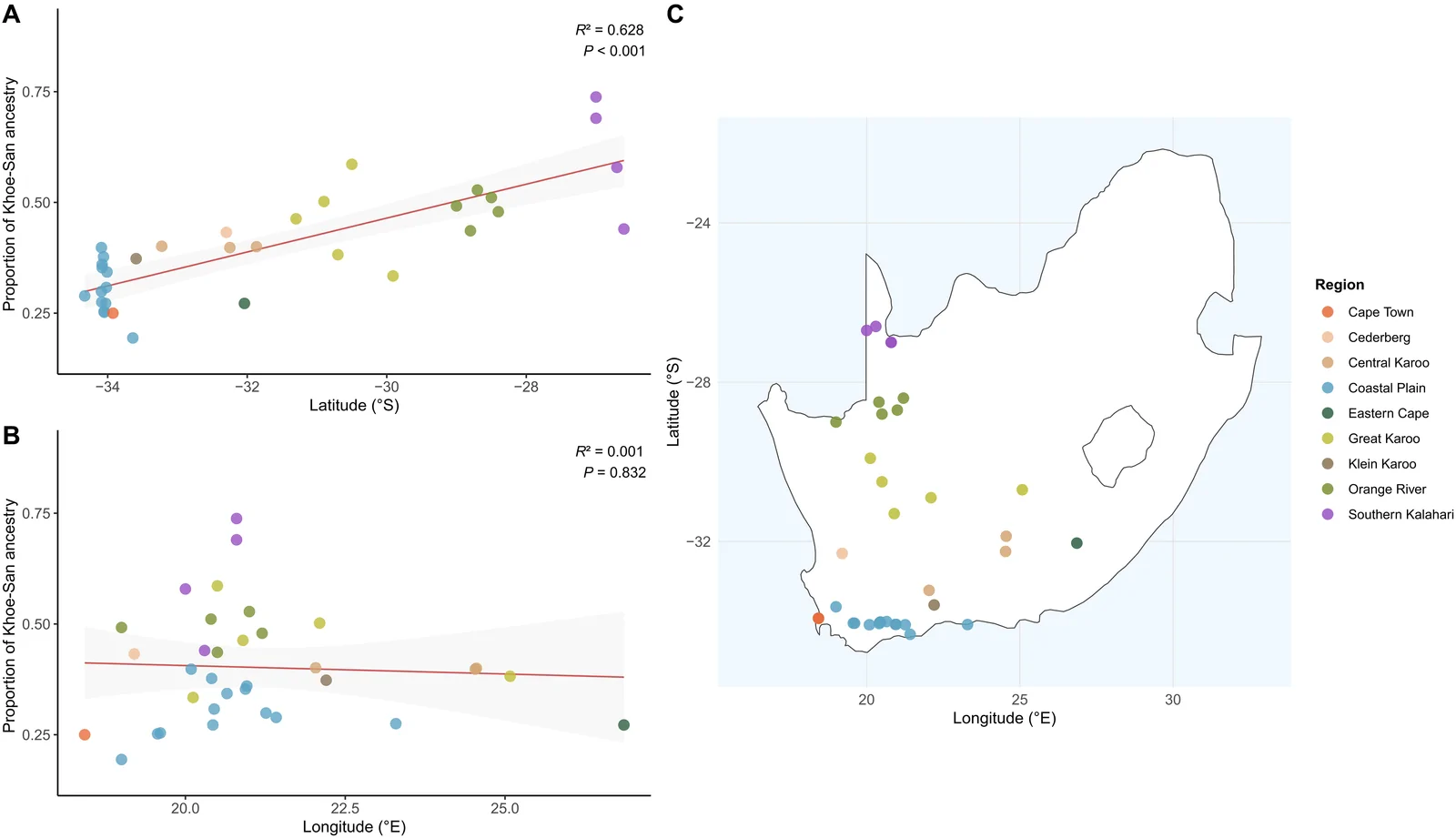
Proportion of Khoe-San ancestry among SAC individuals sampled across South Africa. Khoe-San ancestry proportions were plotted against (**A**) latitude and (**B**) longitude to investigate geographic patterns of admixture across South Africa. Sampling locations for individuals included in this study and previously published samples from Lankheet *et al.* (*10*) are shown in (**C**). Averaged ancestry proportions for newly collected samples were calculated from ADMIXTURE at *k* = 5. Khoe-San groups (e.g., Nama, ≠Khomani, and Botswana) were not included to avoid biasing the overall geographic relationship with their relatively high proportion of Khoe-San ancestry and long distance from Cape Town.

We further ran unsupervised ADMIXTURE (*28*) runs across multiple levels to infer ancestry clusters, setting *k* = 4:10 (Fig. 2A and fig. S1 and S2), with *k* = 9 and *k* = 10 demonstrating the lowest cross-validation error rates (cv error = 0.519; see fig. S3). At *k* = 5, this cluster splits, and we observe ancestries that correspond to European, South Asian, East and Southeast Asian, Khoe-San, and NKA. Because these components are commonly found among South Africans, particularly the SAC (*9*, *14*), we used ADMIXTURE *k* = 5 results to assess the proportion of each ancestry within the South African population and examine the relationship between ancestry and sampling region’s geographical distance from Cape Town. We find an increasing gradient of Khoe-San ancestry with increased distance from Cape Town but only on the axis of latitude, not longitude [coefficient of determination (*R*^2^) = 0.628, *P* < 0.001 and *R*^2^ = 0.001, *P* = 0.832, respectively] (Fig. 3, A and B). The proportion of Khoe-San ancestry ranges from an average of 40% near Cape Town to 80% in the southern Kalahari (Figs. 2C and 3). Conversely, South Asian and East and Southeast Asian ancestries show a negative correlation with increasing distance from Cape Town (Fig. 2C and fig. S4), while the distance from Cape Town explains a lower amount of the variability in European and NKA ancestry (Fig. 2C and fig. S4).

The inferred NKA ancestry begins to segregate into two components at *k* = 6 (Fig. 2A), one of which is the highest in the Himba from Namibia, likely a result of their endogamy and recent bottleneck (*29*). At *k* = 7, the southeastern Asian component distinguishes inferred ancestry in Indonesians from the Han Chinese and Vietnamese. A proportion of Indonesian ancestry is present in the sampled SAC populations, although the extent varies across groups. The CDB exhibits the highest proportion at 3.1 ± 2.1%, followed by the KRO at 2.1 ± 1.4%, while the NCTB and ORG communities show slightly lower proportions, 1.3 ± 1.4% and 1.1 ± 1.3%, respectively. Expectedly, this likely has a correlation with the distance from Cape Town, as previously published SAC samples from District 6, Cape Town, were reported to have a higher Indonesian (∼20%) and South Asian ancestry (21%) (*13*).

Eastern African ancestry begins to differentiate from the other populations at *k* = 8 (Fig. 2A and table S2), shown in yellow (Fig. 2, A and C). The Gumuz show the highest frequency of this component compared to other Ethiopian and Kenyan groups, with smaller proportions also present in the Yoruba and Nama (∼6.5%). The relatively elevated East African ancestry in the Nama has been previously identified by genetic and archeological research resulting from the sheep/goat/cattle pastoralist diffusion around 2 kya (*14*, *30*, *31*). However, this ancestry is a minority among the ≠Khomani San (∼1.5 ± 2.1%) and Botswanan San populations (∼1.5 ± 1.4%), with most of NKA ancestry similar to the Yoruba, Luhya, and Zulu (and variably affiliated with the Himba across *k*’s). The ORG sampling sites (Pella and Augrabies) collectively show higher East African ancestry (3.4 ± 2.3%) compared to the other populations (CDB: 2.4 ± 1.5%; NCTB: 2.0 ± 1.9%; KRO: 1.0 ± 1.1%; see Fig. 2, A and C).

The northern and southern Kalahari Khoe-San ancestries segregate at *k* = 9 (Fig. 2A), primarily differentiating the ≠Khomani San and Nama from the two Kx’a-speaking Botswanan San populations, with the latter representing a northern Kalahari ancestry. Together, the Botswanan San populations harbor the highest northern Kalahari ancestry (76.4 ± 18.5%), relative to other Khoe-San reference populations, and minimal southern Kalahari ancestry (10 ± 6.6%). The majority of the Khoe-San ancestry, both the ≠Khomani San and the Khoekhoe-speaking Nama, is southern Kalahari (60.0 ± 12.7% and 69.5 ± 19.2%, respectively), while the ≠Khomani contain a notably higher proportion of northern Kalahari ancestry (25.5 ± 15.2%) compared to the Nama, who show only 2.2 ± 2.9%. Populations sampled from the CDB, KRO, and ORG and in the Upington area (NCTB) share relatively consistent southern Kalahari Khoe-San ancestry, ranging between 42.0 and 46.8%, while northern Kalahari frequencies are uniformly lower, spanning from 2.6% in the CDB to 9.0% among NCTB participants, similarly to other sampled SAC populations from South Africa (*14*). At this *k* value, we find that the Zulu encompass 16.7 ± 1.7% southern Kalahari Khoe-San ancestry compared to their low northern Khoe-San component of 2.3 ± 0.9% (Fig. 2A and fig. S2).

Last, at *k* = 10 (see fig. S2 and table S3), a new cluster (lime green) appears in a subset of Indonesian individuals. At this resolution, the previously inferred East Asian component (orange; highest among the Han Chinese) found across the CDB, KRO, ORG, and NCTB participants (*k* = 7:9) now clusters with new Indonesian ancestry (fig. S2). The CDB displays the highest proportion of this component, 3.0 ± 1.8%, followed by 2.3 ± 1.7% in the KRO, 1.4 ± 1.8% in the NCTB, and 0.8 ± 1.3% in the ORG. The ≠Khomani San results replicate previously published findings, showing little to no Indonesian ancestry from either cluster (0.005%), while the Nama and Botswanan San show no detectable Indonesian clusters across all *k*’s (<0.001) (*13*).

### Identifying potential sources for the African population structure

**Fig. 4.**
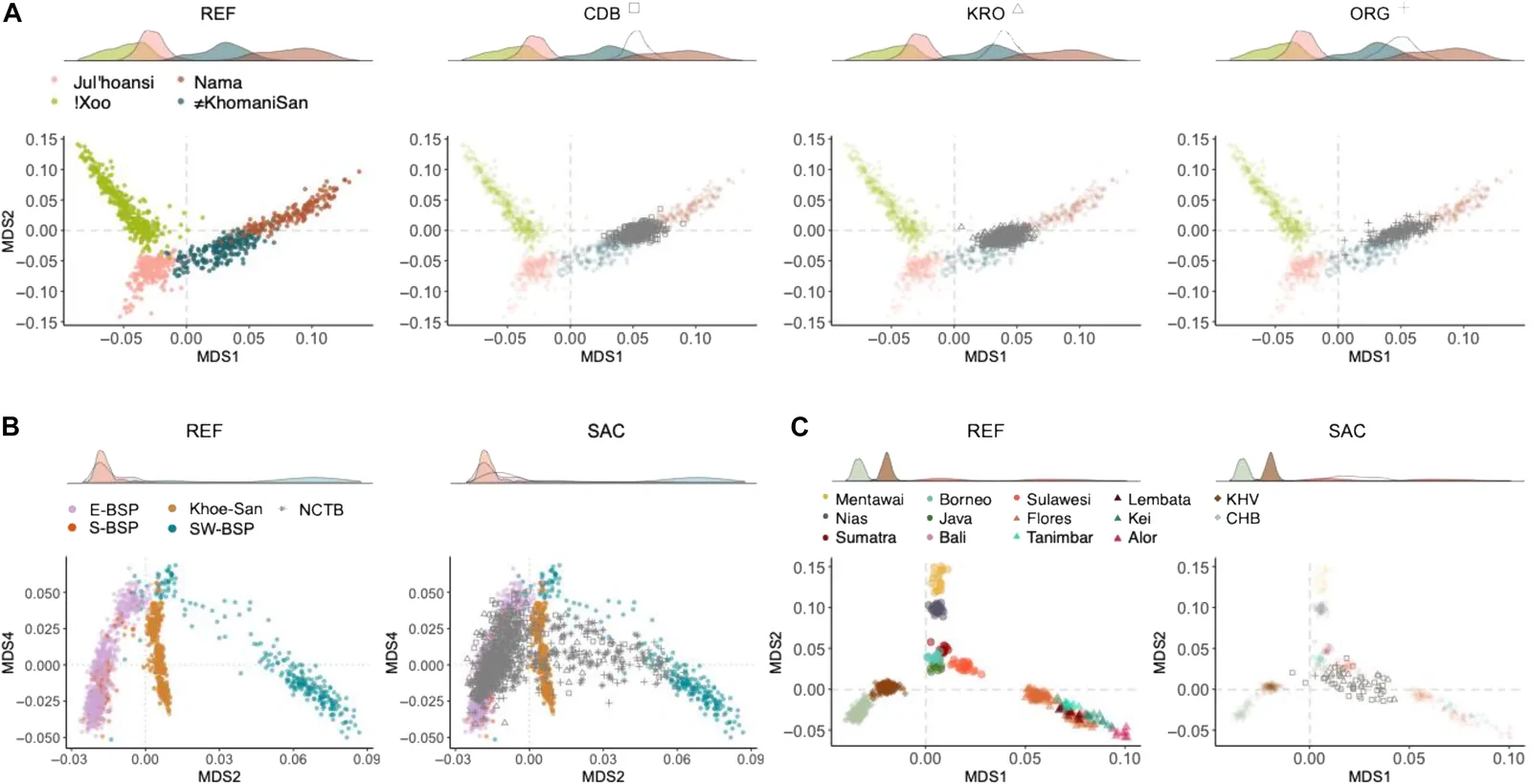
asMDS analysis across three ancestries. For all panels (A to C), the left plot indicates the reference populations, while the right plot projects the queried population’s haplotypes for that local ancestry onto asMDS. Examples were selected for projected haplotypes in the CDB, ORG, KRO, and Upington area (NCTB) cohorts. Haplotypes were filtered for a minimum 75% probability for the respective ancestry being assessed. Shapes for our sampled populations are found beside their respective asMDS. Each asMDS figure is accompanied by a density plot corresponding to asMDS1 above the individual-level asMDS. (**A**) Khoe-San–specific MDS analysis with each sampled populations projected separately as gray shapes. (**B**) NKA-specific MDS analysis. Reference populations are plotted by Bantu language branch, namely Eastern, Southern, and Southwestern Bantu speakers. The right panel includes the CDB-, ORG-, KRO-, and NCTB-projected NKA haplotypes. The Khoe-San individuals here reflect Botswanan San individuals, which control for the low-level Khoe-San ancestry in many of the Bantu-speaking populations along MDS1 (fig. S6). (**C**) Southeast and East Asian–specific MDS analysis. Reference Indonesian samples are labeled and colored by island. Shapes geographically differentiate Asian populations into three regions: mainland (diamond), western Indonesians (circles), and eastern Indonesians (triangles).

To investigate potential sources for the African population structure, we performed multidimensional scaling (MDS) analysis on our sampled populations from the CDB, KRO, Upington area (NCTB), and the Middle ORG. Ancestry-specific MDS (asMDS) allows for a “zoomed-in” view of specific ancestry components in admixed populations. Local ancestry inference (LAI) was preformed using GNOMIX with five broad ancestry reference groups (Khoe-San, European, South Asian, NKA, and East/Southeast Asian) (*32*, *33*). Ancestry-specific haplotypes were then projected via asMDS (see Methods; Fig. 4, A and B) (*32*). We aim to best characterize specific source populations for the five primary ancestries within the admixed study populations.

Our Khoe-San asMDS analysis incorporates South African and Botswanan Khoe and San groups with diverse Khoisan languages. In particular, the reference populations are the !Xoo, ≠Khomani, Ju|’hoansi San, and the Nama (Fig. 4A) (*34*, *35*). In addition, we included a control group, the Bantu-speaking Zulu from southeastern South Africa, who have previously been characterized to have ∼20% Khoe-San ancestry (fig. S5) (*36*). We replicate the previously observed structure among the four Khoe-San reference populations, with the first axis largely distinguishing between the northern and southern Khoe-San ancestries (i.e., structure between the Botswanan San and the ≠Khomani San and Nama) (*20*, *37*). The !Xoo and Ju|’hoansi group (originally subsumed under “BSAN”) centroids are distinct from ≠Khomani San and the Nama (table S4). The second axis separates the !Xoo and Ju|’hoansi from one another. Khoe-San inferred haplotypes from the CDB, KRO, and NCTB and the Middle ORG cluster between the overlapping tail ends of Nama and ≠Khomani San haplotype distributions along asMDS 1 (Fig. 4A), although all study populations’ centroids lie closer to the ≠Khomani San than the Nama (table S4).

For the NKA asMDS analysis, we included previously published Bantu-speaking groups from Botswana, Mozambique, Namibia, South Africa, and Zambia (*25*). Two Botswanan San (!Xoo and Ju|’hoansi) groups were also included in the analysis to account for the low levels of Khoe-San ancestry commonly found among Bantu-speaking populations. The first dimension segregates the Botswanan San from Bantu-speaking populations, while MDS2 separates Namibian Southwestern Bantu speakers from Eastern and Southern Bantu speakers spanning Mozambique, South Africa, and Zambia (fig. S6). MDS3 captures the separation of the two Botswanan San groups. MDS4 reflects a cline of Namibian Wambo, Zambian, and Mozambican Bantu speakers to South African Bantu speakers (fig. S6).

We focus our projections on MDS2 and MDS4 (Fig. 4B), given that these dimensions best capture the geographic structure among southern African Bantu speakers. For all projected populations, a minority of NKA haplotypes is pulled toward the Namibian Himba and Herero, likely reflecting the Damara ancestry self-reported by many individuals (see Discussion). For most of the remaining haplotypes disaggregated by MDS4, the KRO, ORG, and NCTB cohorts trend toward the South African groups more so than those sampled from the CDB mountains (figs. S6 and S7 and table S6). The CDB NKA haplotypes overlap Zambian and Mozambican populations, as well as northern South African Bantu speakers such as the Venda and Tsonga (figs. S6 and S7). The KRO NKA haplotypes are shifted closer to Nguni speakers like the Zulu and Xhosa along MDS4 while noting that there is extensive overlap among South Africa Bantu-speaking populations. The NCTB samples show a similar pattern. Participants from along the ORG overlap both Nguni speakers and Sotho-Tswana speakers; they also contain the largest proportion of individuals pulling toward the Himba and Herero.

### Identifying potential ancestry sources for the non-African population structure

Here, we assess potential ancestry sources for the non-African population structure with asMDS analyses on European, South Asian, and East and Southeast Asian haplotypes. The European asMDS includes five reference populations: British from England and Scotland (GBR), Iberian populations in Spain (IBS), Toscani in Italia (TSI), Finnish in Finland (FIN), and the French. The inclusion of Dutch genomes would have been an ideal European ancestral reference; however, we were unable to obtain access to the available data for our analyses. The local ancestry prediction accuracy was the lowest for this ancestry (89.7%), with most of the model’s confusion reflecting misassignment of South Asian segments as European (6.9%) of the time; the *k* = 5 ADMIXTURE correlation is still high (*R*^2^ = 0.963; figs. S8 to S11). We assess European haplotypes found among our sampled individuals and within ≠Khomani San and Nama individuals with less than 85% Khoe-San ancestry and filtered our study samples for those with at least 20% European ancestry. We find that European-assigned haplotypes in our sampled populations cluster within and between the British and French populations (figs. S12 and S13A), although population centroids are closer to the French (fig. S12 and table S5). This pattern is concordant with historical migration from these regions into South Africa (*38*).

The South Asian asMDS is less specific regarding the putative source populations (fig. S13B). We used five reference populations: the Bengali in Bangladesh (BEB); Indian Telugu in the UK (ITU); Punjabi in Lahore, Pakistan (PJL); Gujarati Indians in Houston, Texas, US (GIH); and Sri Lankan Tamil in the UK (STU). As with the European ancestry analysis, we included haplotypes from ≠Khomani San and Nama individuals with less than 85% Khoe-San ancestry and filtered our samples for those with at least 20% South Asian ancestry. We observe a regional cline from northwest to southeast along MDS1, with the Gujarati (western India) and Punjabi (Pakistan) anchoring one end and the Bengali, Telugu, and Tamil anchoring the other. Along MDS2, the Bengali separate from the Telugu and Tamil. Haplotypes found within the CDB, KRO, and ORG participants generally pulled between the Bengali, Gujarati, and Punjabi but are generally scattered, with some clustering toward the Telugu and Tamil (fig. S13B). Although we are unable to definitively state which source populations among these references best captures the South African haplotypes, there is a noticeable increase in South Asian haplotypes derived from CDB samples compared to our other sampled populations, likely reflecting their closer proximity to Cape Town (fig. S13B).

Included in the East and Southeast Asian asMDS analysis are the Han Chinese and Vietnamese from 1000Genomes alongside 130 Indonesians (Fig. 4C). The East and Southeast Asian ancestry comprises a small fraction of the study population’s overall genetic composition, reaching 4.7% in the CDB and as low as 1.4% in the ORG. Given the region’s slave trade and historical record, we hypothesize that Indonesians would best represent this component found among SAC populations. Our LAI model’s accuracy at assigning this ancestry is 96.3% and correlates well with reported *k* = 5 ADMIXTURE proportions (*R*^2^ = 0.943) (figs. S8 to S11).

We refine previous research, which demonstrated that SAC individuals from the Western Cape shift toward Indonesians (*24*), by implementing a haplotype-based analysis using Indonesian genomes sampled across 12 islands and a larger, more diverse SAC cohort (*13*, *24*). The first dimension separates eastern Indonesian islanders from mainland East Asians, while the second dimension captures previously observed regional variation within Indonesia, distinguishing western from eastern islanders (*39*). We find that Indonesians are more accurate proxies for the East and Southeast Asian components by observing clear haplotype clustering from the study populations near Indonesian samples as opposed to the Han Chinese or Vietnamese (Fig. 4C). Here, we observe more specific methods near western islands Sulawesi, Sumatra, and Java, with some trending toward Flores near regions that correspond with major ports during the Dutch East Indian Ocean trade routes (*40*, *41*).

### Geographic predictors of population structure

**Fig. 5.**
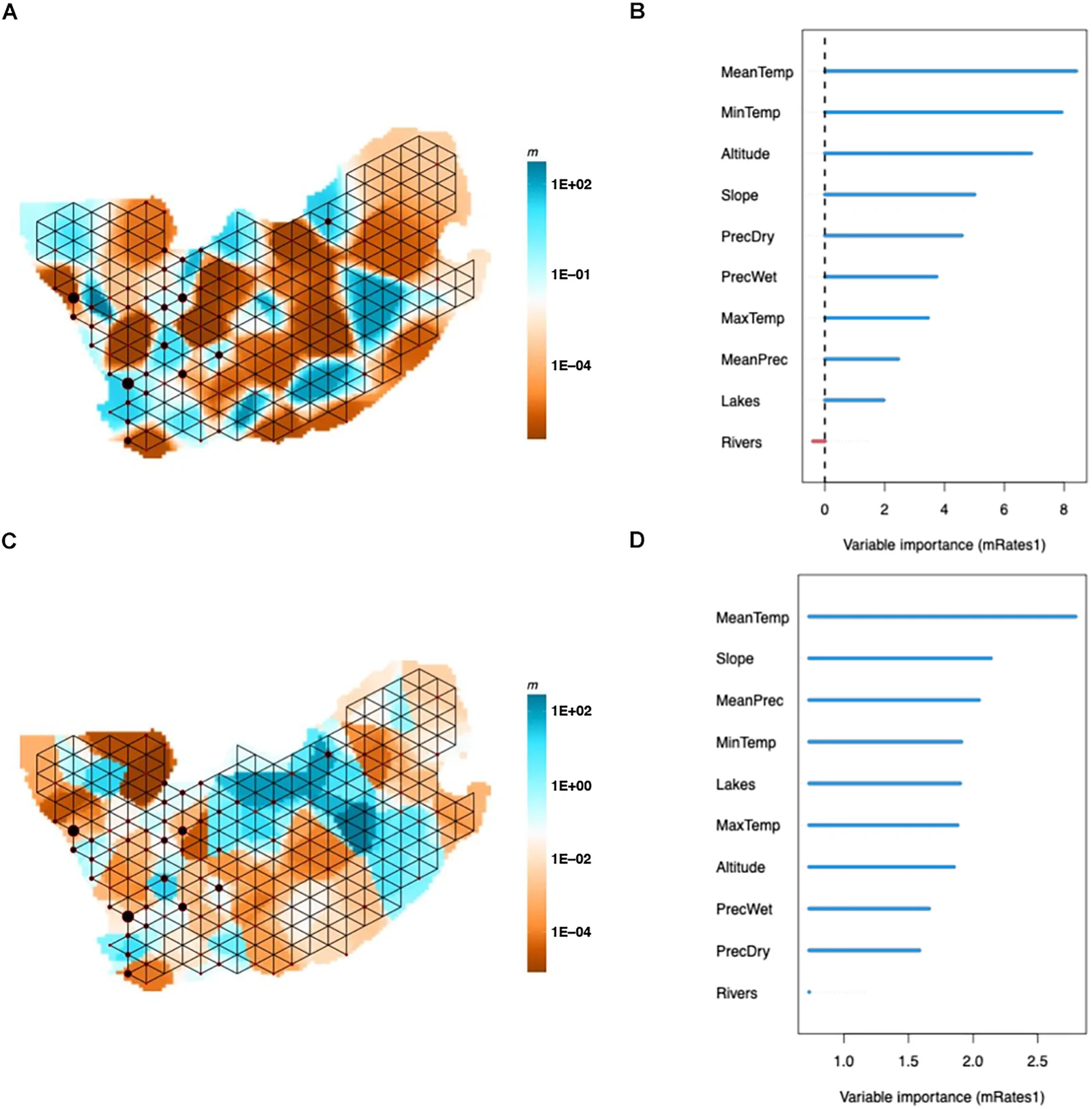
Environmental and geographical factors affect the population structure. In both (A) and (C), the blue color represents higher migration rates and fewer obstacles to migration; the orange color represents barriers to migration and a lower migration rate. The points on the map indicate the sampling locations of the study. (**A**) MAPS results showing estimated migration rates for IBD segments of 2 to 6 cM representing 56 to 13 generations ago. (**B**) SPRUCE random forest importance table with mean annual temperature and minimum temperature during the coldest month being the most important (Pearson’s correlation *R* = 0.259). (**C**) MAPS results showing estimated migration rates for IBD segments greater than 6 cM representing less than 13 generations ago. (**D**) SPRUCE random forest importance table with mean annual temperature and slope being the most important variables explaining the variation in estimated migration rate (Pearson’s correlation *R* = 0.387).

To estimate how geographic and environmental factors affect the human population structure, we used the machine learning tool SPRUCE (Spatial Prediction using Random Forest to Uncover Connectivity among Environments). SPRUCE uses the migration rates and deme outputs from MAPS (Migration and Population Surface estimation) as the dependent variable in the random forest regression (see the “SPRUCE” section) (*42*). The inferred symmetric migration rate (*m*) is converted to dispersal distance (*s*) by MAPS, “which can be interpreted roughly as the expected distance an individual disperses in one generation” (*42*). We estimated two time periods, ∼56 to 13 generations ago [individual IBD segments of 2 to 6 centimorgans (cM)] and less than 13 generations ago (individual IBD segments greater than 6 cM) (*43*). The migration surfaces were similar for the two time periods. In Fig. 5C, corresponding to ∼13 generations ago, we see that there are slightly more barriers to migration in the CDB and relatively more migration within the Greater KRO compared to 56 to 13 generations ago (Fig. 5A and figs. S14 to S17). Random forest regression indicates that environmental variables remain similar across time periods, although some variation exists (figs. S18 and S19). The most important variables explaining variation in migration rates are mean temperature, slope, and minimum temperature during the coldest month (Fig. 5, B and D, and fig. S18).

**Fig. 6.**
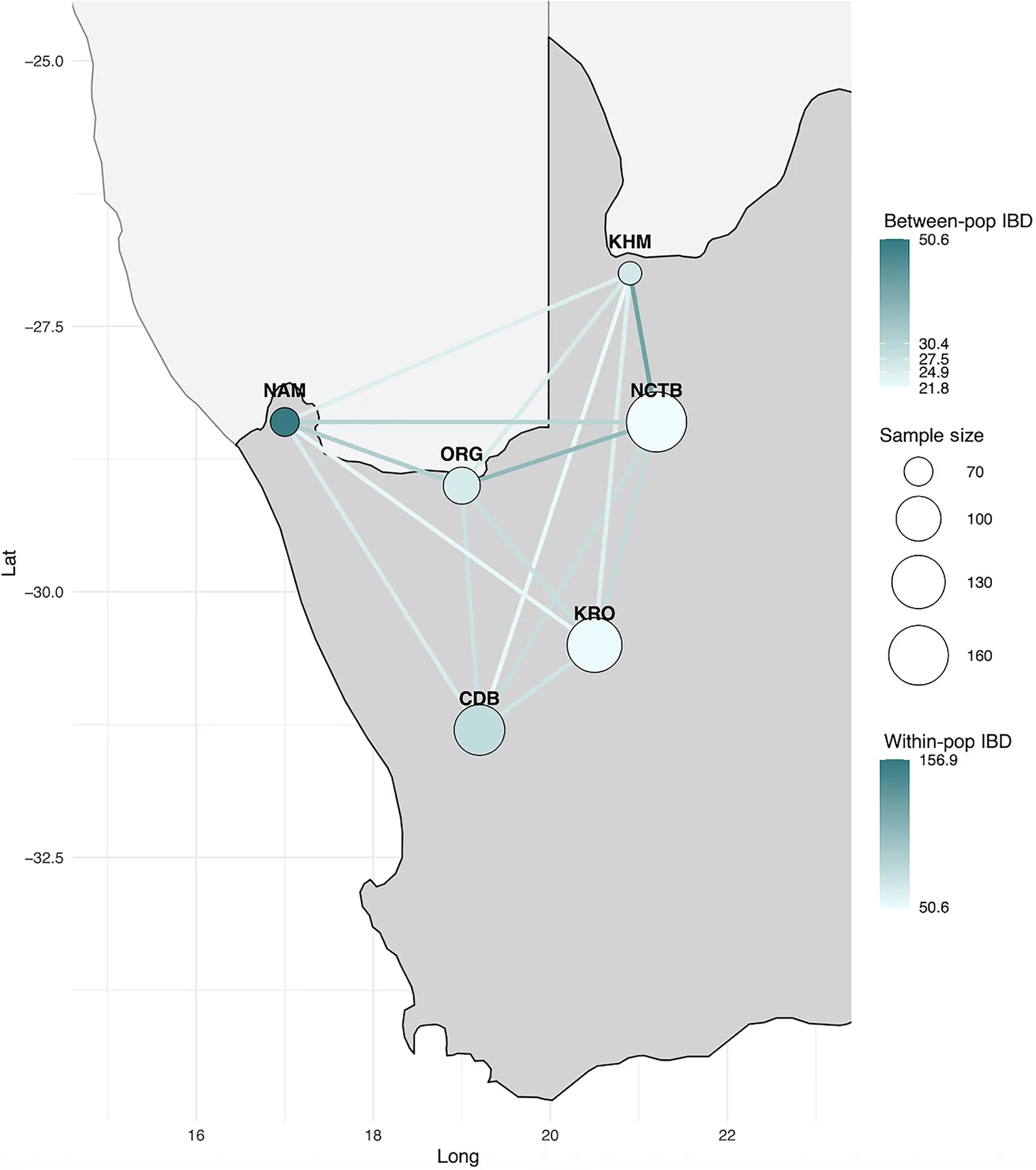
IBD sharing indicates low levels of migration across communities. The nodes are located on geographical sampling coordinates from each community and are scaled by the number of individuals sampled in that region. The nodes are colored on the basis of within-community mean IBD sharing. The edges between groups are colored on the basis of mean IBD sharing between communities.

To better understand the demographic connections in relation to geography, we further investigated identity-by-descent (IBD) sharing within and between communities using GERMLINE2 (see Methods). We see higher mean shared IBD segments within communities than between, with the Nama having the highest mean shared IBD (Fig. 6 and fig. S20). The IBD network shows patterns of sharing between communities that are consistent with the expectation that geographically closer communities are more likely to share IBD than more distant groups. However, this relationship is relatively weak, suggesting that other social and geographic factors may play a role.

## DISCUSSION

The genetic diversity of present-day South African populations has been significantly shaped by a series of complex migrations over the past 2 kyr. Previous studies have predominantly focused on disentangling the Khoe-San population structure and understanding deep human evolutionary history with relatively less attention given to colonial impact and population dynamics (*11*, *21*, *44*–*46*). There is limited knowledge of how colonization affected the genetic ancestry of Indigenous populations in South Africa, largely due to the systematic displacement of these populations during and after the colonial period. Today, few individuals self-identify as members of South Africa’s Indigenous Khoe and San groups.

Previous studies have examined the complex ancestry components of SAC individuals in Cape Town and communities along the southwestern coast (*14*, *31*). On the basis of a large-scale study of more than 600 participants, we provide a comprehensive genetic analysis of SAC populations outside of Cape Town (*n* = 620) and characterize the genetic composition across multiple regions of South Africa [CDB, KRO, southern Kalahari (Upington area; NCTB), and Middle ORG; Figs. 1 and 2C). Our data demonstrate that the SAC population in the northwest of the country is shaped by multiple sources of gene flow, consistent with prior findings in Cape Town and the surrounding areas (*11*, *21*, *23*, *47*, *48*). European colonization introduced not only European ancestry but also the forced migration of enslaved people through the Dutch East India (VOC) slave trade. This brought additional ancestry components into Indigenous Khoe-San populations, including gene flow from western and eastern Africa, South Asia, Malayasia, Madagascar, and Indonesia (fig. S2) (*11*, *14*, *21*, *23*, *31*, *47*, *48*). Using asMDS, we were able to differentiate between putative ancestral source populations for the five ancestries commonly found within SAC communities (*47*, *48*) and identify better proxies for these sources (*21*). While we have made an effort to collect comprehensive and representative population genetic datasets from the contemporary residents of the regions that are the putative sources of these ancestries, large datasets of high-coverage genomes from precolonial samples from these regions would be necessary to further refine the source populations contributing to SAC ancestry components.

### Western African ancestry reflects both ancient Bantu expansion and colonial slave trade

Our analysis reveals distinct patterns of admixture timing and intensity across the CDB, KRO, and Middle ORG/Upington areas. NKA ancestry varies geographically, from ∼11% in the CDB to about 22% in the KRO and along the Middle ORG (tables S4 and S7; ADMIXTURE *k* = 5). This distribution likely captures signatures of the Bantu expansion, with this component accounting for roughly 80% of genetic composition among southern Bantu-speaking Zulu, or it may represent NKA lineages introduced by the VOC’s slave trade.

Previous studies have found the NKA admixture into the Khoe-speaking hunter-gatherers and Kxa-speaking hunter-gatherers to vary in timing, ranging from as early as 1.2 to 1.5 kya (*20*). The Richtersveld Nama show a pulse of NKA gene flow at 700 years ago. These dates suggest that much of the gene flow observed in the northern Khoe-San populations dates to the initial wave of Bantu expansion across the Kalahari (*25*, *26*). However, another wave of NKA ancestry was introduced to the Cape Colony as the VOC forcibly relocated enslaved individuals from Madagascar and Mozambique and along the Swahili Coast (*3*). The positioning of CDB individuals near Mozambican and southeastern African populations (Fig. 4B) is consistent with the historical role of Mozambique as a major Portuguese slave trading hub in the Indian Ocean slave trade, through which enslaved individuals were transported from across the African continent (*49*). Rather than reflecting a single western Bantu-speaking source population, this dispersed pattern along MDS4 suggests that the NKA ancestry present in the CDB more likely represents descendants of individuals with diverse African origins. These multiple waves of admixture are further supported by tract length patterns in the CDB mountains. The decay pattern of NKA tracts resembles that observed for South Asian ancestry, consistent with colonial-era admixture events. This contrasts with tract length profiles in KRO and Middle ORG participants, which indicate older gene flow (fig. S11).

After performing asMDS, the NKA component often closely resembles southeastern Bantu-speaking groups rather than southwestern Bantu-speaking populations (Figs. 2 and 4). In the KRO town of Carnarvon, this ancestry is consistent with self-reported Xhosa parents or grandparents among study participants. Conversely, the Nama and Middle ORG participants have higher proportions of southwestern Bantu-speaking ancestry (∼5.6 and 9.5%, respectively) relative to other sampled populations (≤3.4%). This pattern positions their population centroids intermediate to the Himba and southeastern African reference populations in the NKA asMDS analysis (Fig. 2C and figs. S6 and S7). Ethnographic data from participants sampled along the Middle ORG frequently report being “Nama-Dama” or having a Damara ancestor. The Damara are a historically mobile population who today resides in the semiarid regions of Namibia and Northern Cape, South Africa (*22*, *50*).

### Indonesian-ancestry and other intercontinental migration

Initially established as a refueling station in 1652, the Cape Colony (now known as Cape Town) became the gateway for intercontinental migration into southern Africa. While many of the early colonists were Dutch, migrants from other western European regions also became part of the Cape Colony. Protestant refugees (known as the French Huguenots) arrived in the late 17th century fleeing religious persecution. They lived in South Africa under Dutch rule in areas like Franschhoek and Drakenstein Valley in the mountainous region to the northeast of Cape Town (*51*). The British gained control of the colony from the Dutch in 1806 (*52*, *53*). Reflecting the European colonial history at the Cape, our study populations retain European haplotypes intermediate to French and British populations in the asMDS analysis, with population centroids positioned closer to the French (fig. S12). Although French and British populations serve as reasonable proxies, a limitation of our study is the lack of access to Dutch reference genomes, which would have been most appropriate given Dutch colonial history.

The VOC’s extensive Indian Ocean trade network also facilitated the forced migration of enslaved populations from Asia to the Cape Colony (*54*). Distinguishing among Asian sources has been challenging in previous studies, potentially due to methodological constraints, limited sampling, genetic drift following initial admixture in South Africa, or a combination of these factors (*13*, *24*). We find a noticeably higher signal of South Asian haplotypes in the CDB samples compared to the other sampled locations, likely due to their proximity to Cape Town (fig. S21) (*14*). Although our analysis yields ambiguous results for distinguishing among specific South Asian regional ancestries, further methodological developments could potentially refine these haplotype assignments.

In contrast to the ambiguous South Asian patterns, our analysis is able to provide further resolution to East/Southeast Asian ancestry components in the SAC. The asMDS for this component clearly points to Indonesia as the best proxy source population, rather than the mainland Asian populations (e.g., Han Chinese) typically used to capture this signal (Fig. 4C) (*11*, *14*, *48*). Inferred proportions of East/Southeast Asian ancestry in our study populations is relatively low, ranging from trace levels (mean <1%) in the ≠Khomani San to about 6% in the CDB, following a south-to-north gradient as seen in some other ancestry components (fig. S4 and table S7). We included only those whose combined maternal and paternal haplotype proportions reflected at least 10% Southeast Asian ancestry, which is a limited proportion of the study cohort. In addition, input segments were required to have a minimum probability of 0.75 to be classified as East/Southeast Asian. Although the low proportion of East/Southeast Asian ancestry and the applied thresholds introduced some limitations, they also increased confidence in the results.

Detection of this Indonesian component in the CDB and other sampled regions aligns with historical records (*55*, *56*). An estimated quarter of all enslaved individuals at the Cape Colony were of Malay or Indonesian descent (*57*). The clustering patterns observed in our southeastern Asian asMDS correspond with major VOC trade routes connecting South Africa to predominantly western Indonesian islands (*40*, *41*). Islands such as Sulawesi, Sumatra, and Timor had formalized “slave clauses” with the Dutch in the 1600s (*35*). We see higher proportions of the South Asian component than the Southeast Asian component, approximately double the Southeast Asian inferred global ancestry proportions across populations. This pattern may reflect that notion that enslaved individuals from Indonesia remained concentrated in the central Cape Colony rather than dispersing northward or that those who did migrate north, whether through forced relocation or escape, experienced demographic decline, resulting in their minimal genetic contribution to the SAC populations outside of present-day Cape Town.

### SAC populations retain high Khoe-San ancestry

The VOC initially relied on livestock trade with Indigenous populations during the first 30 to 40 years of their occupation at the Cape Colony but made little effort to expand or incorporate Indigenous groups into the colony (*58*). However, as European colonists began expanding outward from the Cape Colony in the 17th century, they successively forced Indigenous populations into the interior and more marginal areas such as the Kalahari and CDB mountains (*6*). However, colonial ambitions intensified significantly in the 1730s, leading to heightened Indigenous resistance. A frontier war erupted between the colonists and the Khoe-San living in the CDB in 1739 (*59*), followed by significant population decline resulting from smallpox epidemics. Historical records indicate that many surviving Khoe and San individuals were forced into servitude and employed as laborers on settler farms (*60*). In some cases, these Indigenous individuals admixed with those enslaved by the VOC, forming new communities that lead to people who largely self-identify as SAC today (*61*).

The systematic displacement and marginalization of Khoe-San populations during and after the initial European colonization have led to a widespread belief in contemporary South Africa that Khoe-San ancestry is largely absent from modern populations, yet our analysis demonstrates substantial Khoe-San genetic contributions across study populations. Our results indicate that SAC populations from the Western and Northern Cape Provinces retain a substantial proportion of their genetic ancestry from Khoe-San populations, averaging 41 to 53% (*11*). Haplotype-based analysis of Khoe-San segments (with >75% posterior probability) finds this component to be more similar to the southern Kalahari ancestry—represented by the ≠Khomani San and Nama—rather than the northern and central Kalahari, represented in this study by !Xoo and Ju|’hoansi (Fig. 4). This result corresponds with the sampling sites’ geographic location south of the Kalahari (Figs. 1 and 2C) (*50*). Previous studies have shown a clear north-south structure in Khoe-San genetic diversity among modern populations, with more recent work suggesting an even more geographically complex pattern, reflective of the Kalahari Desert as a barrier to gene flow (*20*, *21*, *62*–*64*). The population centroids of the KRO and ORG in our asMDS results are closer to the ≠Khomani San than the Nama (fig. S22 and table S3), with the CDB situated intermediate.

We hypothesize that the observed patterns could result from one or more of the following: (i) European disruption led to widespread displacement and increased gene flow among Khoe and San populations south of the Kalahari, resulting in a Khoe-San ancestry signature generally intermediate between the ≠Khomani San and Nama. (ii) A gradient of ancestry between the Nama and ≠Khomani could reflect a demic diffusion of Khoekhoe pastoralists both along the western coast and along the ORG into the interior. If the diffusion incorporated local hunter-gatherer populations, rather than with replacement (*65*), such a process could fit the asMDS. (iii) Last, the Richtersveld Nama have experienced a severe postcolonial bottleneck; it is possible that their extreme MDS values represent localized drift, separating them from similar groups (such as CDB Khoe-San). Future research collecting additional samples from individuals in Namibia and formal demographic modeling are necessary to address these hypotheses.

### Colonial impact on ecogeographic population structure

Colonial-era processes, centered at Cape Town and extending outward through systematic displacement and the VOC trade network, have created spatial gradients in the distribution of the five main ancestry components. Using ADMIXTURE *k* = 6 results, we considered how ancestry proportions varied across sampling regions. We observe a gradient of increasing Khoe-San ancestry with increased distance from Cape Town on the axis of latitude, following colonial patterns of systematic displacement of the Khoe and San groups (*6*), but observe the opposite in European, South Asian, and southeastern Asian ancestries (Fig. 3A and fig. S4). We find higher proportions of European and Asian ancestries in individuals closer to Cape Town, likely because Cape Town was the initial heart of the settlement (*3*, *14*). Unlike the other ancestries, the proportion of NKA ancestry has no relation with the sampling site’s distance from Cape Town (fig. S4), reflective of the demographic history mentioned above.

In addition to these geographic gradients of ancestry, our results reveal how European colonization fundamentally altered population movement and structure across the landscape. Using effective migration rate analysis of shared IBD tracts among our study populations, we compared two distinct time periods: precolonial (approximately 450 CE to 1650 CE; 56 to 13 generations ago) and colonial (1650 CE to present; <13 generations ago) (*57*, *66*). During the precolonial period, we observe higher migration rates along the CDB and within the arid Greater KRO interior, with natural barriers to movement between these regions (Figs. 1 and 5A). The CDB’s rugged landscape may have constrained population movement, while the comparatively flatter semiarid KRO allowed for greater mobility within the region.

Colonial contact altered these migration patterns, resulting in a decrease in migration rates along the coastal plain and the KRO (Fig. 5, A and C). European colonization systematically restricted Indigenous movement through natural resource monopolization, policies limiting individual mobility, and military outposts protecting colonial boundaries (*6*, *67*). Colonial control of water resources became a particularly powerful tool for controlling Indigenous populations, limiting Khoe-San movement and undermining pastoralist autonomy (*6*). This shift is reflected in our environmental modeling: Water resources show low importance in predicting precolonial migration rates, but the presence of lakes becomes significantly more important postcontact, potentially reflecting increased sedentism during the colonial period (Fig. 5, B and D) (*60*).

As the British took control of the Cape Colony from the Dutch in the early 19th century, the land became the property of the Crown, and Indigenous people were viewed as obstacles to colonial success (*68*). Legal restrictions further constrained mobility—the colonial government’s 1809 law requiring Khoe-San individuals to adopt sedentism institutionalized the restriction of movement and facilitated labor control (*69*). These policies systematically dismantled the traditionally mobile lifestyles of Khoe and San populations, forcing cultural assimilation as European colonizers imposed colonial norms while devaluing Indigenous lifeways as “primitive.” The persistence of this disruption is evident today, as most Khoe-San descendant individuals speak Afrikaans and English rather than traditional click-phoneme languages (*50*).

This study provides a large-scale characterization of genetic composition across multiple regions in South Africa, revealing that Khoe-San ancestry constitutes a significant proportion of ancestry among SAC populations (41 to 53%) from CDB, Great KRO, and Middle ORG. Despite historical colonial disruption, our results show that the Khoe-San descendant communities retained a significant portion of Khoe-San genetic ancestry. Using local ancestry assignment, we are able to further pinpoint possible sources for colonial-era migration into South Africa. For example, we identify Indonesia as the likely source population for the previous “East Asian” genetic component, further supporting the historical record that a fourth of all enslaved individuals at the Cape Colony were of Malay and Indonesian descent. This ancestry remains a small minority in our sampled individuals, however. The substantial Khoe-San ancestry persisting in modern populations, the geographic gradients reflecting colonial settlement patterns, and the disrupted mobility patterns evident in contemporary genetic structure all demonstrate how colonial processes fundamentally altered the demographic landscape of South Africa. We emphasize that genetic signatures alone do not fully capture the complex power dynamics that shaped these demographic changes. Understanding the genetic legacy of colonization requires recognizing both what these patterns reveal about historical processes and the limitations of genetic data in capturing the full scope of colonial impact.

## METHODS

### Sample collection

Genetic and demographic data from several towns in the CDB, KRO, and ORG were newly collected between 2017 and 2023; locations are indicated in Fig. 1. DNA from saliva samples was collected with Oragene OGR-600 kits (DNA Genotek), and ethnographic information regarding ancestry, language, and parental place of birth was collected for all participants. Institutional review board approval was obtained from Stony Brook University (727494), University of California-Davis (1220180-7), and the Health Research Ethics Committee of Stellenbosch University (N11/07/210 and N11/07/210A). All individuals gave signed written and verbal consent with a community witness present before participating. Participants were asked to self-identify without categorical prompts. Before sampling, municipal or nonprofit leaders in each community were consulted on the aims and approach of the project. Community-level results were returned to most of towns between 2023 and 2025, including community meetings and pamphlet and poster distribution paired with discussions involving local municipalities, high schools, and community members.

### Ethnic composition of study participants

Study participants were recruited from communities of predominantly Coloured census, the Middle ORG, CDB, and KRO (Fig. 1). To avoid racial categories, participants were asked to self-identify their ethnicity without prompts/examples from interviewers. A total of 79% of participants chose Coloured as their primary ethnicity, whereas 12% chose either “Khoe-San” or a specific Khoe-San descent ethnicity, such as Nama. The remaining individuals chose a Bantu-language ethnicity (<4%) or provided a more complex answer such “I am South African” or “I am mixed.” The self-identified ethnicity of participants included in this study from a clinical cohort of the Middle ORG region are 88.4% Coloured, 4.6% Tswana, 4.2% Khoe-San (e.g., Nama and San), and 1.9% as “other” (*70*).

### Sample consolidation, genotyping, and PCA

Newly collected samples were genotyped for >2.2 million SNPs using the Illumina H3Africa array. Raw genotype data were processed using Illumina’s GenomeStudio to call common variants (minor allele frequency >0.05) on the autosomes and X- and Y-chromosomes, followed by zCall to call rare variants. Details of this pipeline are available at https://github.com/hennlab/snake-SNP_QC. All datasets were aligned to the 1000 Genomes Phase 3 reference panel, and all ambiguous variants were removed. Variants with a genotype missingness rate greater than 5% were removed using PLINK’s --geno filter, and individuals with more than 10% missing genotypes were excluded using the --mind filter. The remaining variants were then pruned for linkage disequilibrium using a threshold of *r*^2^ < 0.3.

We consolidated a reference panel incorporating data from four published genome sequencing projects, the African Genome Variation Project (AGVP) (*71*), Human Genome Diversity Project (HGDP) (*72*), Indonesian Diversity Genome Project (IDGP) (*73*), 1000 Genomes Project (1000GP) (*74*); 94 ≠Khomani San whole-genome sequences (*75*); previously published African samples (*76*) genotyped on the Omni5 array; and Bantu-speaking groups genotyped on the H3Africa array (*25*). All Nama participants were sampled from Richtersveld, South Africa, and data were previously published by Martin *et al.* (*75*).

We extracted populations of interest from each dataset listed above. The Omoro, Amhara, Wolayta, Somali, Zulu, and Gumuz data were extracted from the AGVP (*71*), and the Himba data were previously published by Scelza *et al.* (*77*). Indonesian populations from Mentawai, Nias, Sumatra, Borneo, Java, Bali, Sulawesi, Flores, Tanimbar, Lembata, Kei, and Alor were sourced from the IDGP (*73*). The data of British (GBR), Iberians from Spain (IBS), Han Chinese (CHB), Vietnamese (KHV), Yoruba (YRI), Luhya (LWK), Bengali (BEB), Tamil (STU), and Gujarati (GIH) were obtained from 1000GP (*74*). Last, the French data were obtained from HGDP and the Botswanan San data from previously genotyped African samples (*76*). The Botswanan San include two San populations, the Ju|’hoansi and !Xoo. However, the published dataset (*76*) did not include population IDs, leaving us to infer these IDs using a combination of methods further detailed in the Supplementary Materials (see figs. S23 to S26). The Botswanan San samples were processed before the lifting or merging with the larger dataset to maximize the variants used in the analysis. This left the Botswanan San on hg19 with 2,993,754 variants, and the NAM, ≠Khomani San, ORG, CDB, NCTB, and KRO aligned to hg38 with 1,015,964 markers. Sample sizes for each population can be found in table S1.

Datasets were filtered for biallelic variants congruent with markers assayed using Illumina’s H3Africa array. With the exception of the included 1000GP and HGDP populations, each dataset was lifted from hg19 to hg38 using Picard Tools LiftoverVCF (*78*). Samples were then merged with the newly collected cohort of South African samples genotyped on H3Africa (*79*). The newly collected samples include SAC individuals sampled along the CDB mountains, KRO, ORG, and NCTB Project. The merged data were filtered for Hardy Weinberg Equilibrium (<0.00005), minor allele frequency (<0.00047 to remove singletons and doubletons), and individual and SNP missingness (>5%) using plink (version 1.9). The compiled dataset contained 806,460 autosomal SNPs and 1165 individuals. Specific to the analyses focused on the NKA component, further filtering was applied to the Bantu-speaking populations from Fortes-Lima *et al.* (*25*), removing variants with 5% missingness and individuals with 10% missingness before merging with the larger dataset. This provided the NKA asMDS analysis with remaining 767,722 variants. Merging was assessed using PCA’s 1-10, with PC loadings generated through smartPCA (*27*). VCF files were converted to “.ped” and “.map” files for eigensoft smartPCA input.

### Phasing

The dataset was phased using the program Shapeit4 (version 4.2.2) (*80*) and the Consortium on Asthma among African Ancestry Populations (CAAPA2) reference panel (*81*). This reference panel includes 78 ≠Khomani San and 49 Nama individuals, in addition to other continental African populations, making it the most representative reference panel available for the individuals sampled in this study. All Shapeit4 parameters were set to default, with the exception of “--mcmc-iterations” being set to “10b,1p,1b,1p,1b,1p,1b,1p,10m.”

### Identifying relatives

We used Parent Offspring Pedigree Inference Robust to Endogamy (PONDEROSA), a software designed to identify relatives within more endogamous populations and/or populations that have undergone recent bottlenecks, resulting in elevated levels of shared IBD regions (*82*). The program provides an option to simulate related individuals from the population for various relationship classes (i.e., maternal/paternal half-siblings, maternal/paternal grandparents, and avuncular relationships). It accomplishes this by integrating the program ped-sim (*83*–*85*). The IBD distributions for the simulated relationships are then used to train the model that will be applied on the real data.

PONDEROSA was independently preformed on each of the southern African populations, including the newly collected SAC individuals. We segregated the ≠Khomani San, Nama, CDB, KRO, ORG, and NCTB samples by region/population and proceeded to run simulations on each of the six southern African groups included in this study.

With the exception of the Botswanan San, samples were lifted from hg38 to hg19 without variant loss; to use PONDEROSA, this required a sex-recombination map aligned to hg19 (*83*–*85*). IBD calling was then carried out using PONDEROSA’s built-in script initiating phasedIBD (*86*), with all settings set to default. We simulate 200 pairs of individuals for each population and then use the training classifiers to identify relatives in the populations. Relatives having a greater than 80% probability were assigned a specific class, while those below the threshold were assigned either second-, third-, or fourth-degree relatedness. Last, we used the PONDEROSA “remove_relatives.py” script to filter individuals who were related closer than third degree. This script does not remove all relatives but rather retains one of the related pair of individuals to maximize the number of unrelated individuals. For example, if a sibling pair existed in one of the populations, the function would retain one of the siblings in the unrelated subset of samples while placing the other in the relative’s subset.

### ADMIXTURE

Before running ADMIXTURE, we first identified all first- and second-degree relatives using KING (*87*) and partitioned relatives into multiple non-overlapping “running groups” (RGs). We then performed separate ADMIXTURE analyses for each RG. ADMIXTURE assumes that all samples are unrelated because shared IBD tracts between close relatives can bias allele-frequency estimates and consequently inflate or deflate inferred ancestry proportions. Excluding close kin from each run ensures unbiased allele-frequency estimates, while systematically rotating relatives across groups guarantees that every individual contributes to at least one model fit.

The three Khoe-San reference populations were independently assessed for relatedness using PONDEROSA (as above). An internal R script was used to generate (i) an unrelated subset of individuals, (ii) randomized groups of first- and second-degree relatives while ensuring minimal overlap of related individuals across groups, and (iii) a list of unrelated individuals in the dataset. A total of 10 groups were created to divide relatives; each then merged with the larger list of unrelated individuals. Corresponding PLINK files were generated for each of the final 10 RGs.

The three southern African reference populations underwent a similar process independently of one another. However, instead of partitioning all first- and second-degree relatives into different RGs, we retained one individual from each related pair in the unrelated subset using the PONDEROSA “remove_relatives.py” script. Individuals classified as relatives were divided into 10 randomized groups and added to their respective RG PLINK files with the core set of unrelated individuals. This approach allowed us to retain the maximal number of Khoe-San individuals in the core (an unrelated set of individuals shared across the RGs), while the related individuals were randomly distributed across the 10 RGs, minimizing overlap among relatives. A flowchart of this pipeline is provided in fig. S1.

European and Asian populations were thinned to only include the GBR, IBS, GIH, BEB, SES, CHB, KHV, and Indonesians in admixture. Linkage disequilibrium was calculated on the merged dataset of 806,460 markers, including the reference populations and newly genotyped southern African samples using PLINK 1.9 (*88*). Data were conservatively pruned using the PLINK --indep-pairwise flag for markers with a linkage disequilibrium of 0.5 or greater within a window size of 50 SNPs, with a sliding window size of 20 SNPs. The pruned dataset contained 479,497 variants. Ten iterations of ADMIXTURE *k* = 4:10, with *k* being the number of clusters ADMIXTURE infers, were performed for each RG, all with fivefold cross-validation and randomly selected starting seed. With respect to the level of *k*, results for each RG were averaged across individuals. ADMIXTURE results were visualized using pong (Fig. 2A and figs. S2 and S3) (*89*). For the analysis comparing Khoe-San ancestry proportions to geographic distance, we included previously published estimates for additional South African samples from Lankheet *et al.* (*10*), alongside the 620 newly collected samples, to provide further geographic coverage of the country.

### Local ancestry inference

Local ancestry was inferred using Gnomix (*33*), which has been shown to outperform other programs in accuracy and speed. Similar to other LAI programs, Gnomix treats each reference population as an independent ancestral group without considering the relationships between populations. In other words, the individuals within each ancestral group must have a high ancestry proportion with respect to the ancestral group they are representing.

This was a relatively simple requirement to meet for most of the references but particularly difficult when using reference populations who are admixed, such as the southern African reference individuals. On the basis of the ADMIXTURE results for *k* = 6, we filtered for ≠Khomani and Nama individuals having at least 85% Khoe-San ancestry and the Botswanan San for minimum 90% Khoe-San ancestry. We chose to use varying cutoffs to retain the maximum number of individuals while accounting for within the Khoe-San population structure and differences in sample sizes. Southern African individuals below the cutoff were then added, along with the Zulu as a control, given that they are expected to have 15 to 20% global Khoe-San ancestry (*36*). Including the Zulu in the query helped verify that the summarized local ancestry proportions within the population matched expectations, providing confidence in our LAI model.

Given that the SAC populations are often described as five-way admixed, we assembled a LAI reference panel that accounts for each putative ancestry. Putative source populations for the KHS components in our study samples include the ≠Khomani San, Nama, and the Ju|’hoansi and !Xoo from Botswana. Putative source populations for European ancestry include GBR and IBS (EUR); those for South Asian ancestry include BEB, GIH, and STU (SAS); and those for East/Southeast Asian ancestry included CHB, KHV, and Indonesians (EAS). We separated the KHS populations, that is, the Nama, ≠Khomani San, and Botswanan San, from other Africans and aggregated all NKA populations into an independent category. We opted to use this method because (i) the Khoe-San are highly diverged from other NKA populations, providing confidence that we can distinguish between Khoe-San and NKA haplotypes, and (ii) the study samples likely have NKA ancestry from various source populations, making it difficult to discriminate between the putative populations. Populations included in NKA are the Yoruba, Luhya, and Gumuz. The Omoro, Amhara, Wolayta, and Somali individuals were removed from the NKA reference panel because of the back-to-Africa (*90*) ancestry, which weakened the model’s assignment of EUR and NKA segments. In summary, our five-binned local ancestry reference panel had populations representing EUR, SAS, EAS, KHS, and NKA ancestry.

Gnomix (*33*) was executed using the recommended array config, a logistic regression base, and xgboost smoother modules. All settings were set to default, with the exception of adjusting for a larger window (0.4 cM) and smoother size (*82*). We assessed multiple models and found this model to have the most accuracy and to be more stable against phase switching because of the increased window and smoother size. These changes reduced sensitivity with a decreased window size but increased model accuracy. Local ancestry results were evaluated using five methods: (i) a confusion matrix, (ii) ADMIXTURE and Gnomix global ancestry correlations, (iii) track lengths, (iv) probability distribution for each ancestry, and (v) visualizing karyograms. See the Supplementary Materials for more details on local ancestry assessment.

### Ancestry-specific MDS

We used multiarray ancestry-specific MDS (maasMDS) (*32*) to evaluate whether the projected ancestry-specific haplotypes produced by GNOMIX in our southern African study samples would gravitate toward one or more putative source populations relative to others. This method calculates the dissimilarities among the assigned reference populations and then incorporates ancestry-specific haplotypes from the study population by their distances relative to the existing reference clusters, effectively projecting them onto the predefined coordinate axes. The analysis was carried out using average pairwise genetic distances, including only haplotypes with at least 75% probability of the target ancestry. Each ancestry had an independent maasMDS run where only the respective putative reference populations were specified. For example, when running NKA maasMDS, the KHS, EUR, SAS, and EAS populations were removed from the analysis. The flag “IS_WEIGHTED” was set to true. Reference populations were given a weight of “1,” while study samples were assigned “0.01” to ensure that they are projected within the MDS space of the reference haplotypes. Visualization of the output data was done using the ggplot2 (*91*) library in R. The asMDS pipeline can be found in fig. S27.

### Euclidean distance computation

Using the coordinates from asMDS1 and asMDS2, we calculated the Euclidean distances between population average distributions. Individuals were assigned to populations on the basis of predefined labels, and for each population, the MDS1 and MDS2 centroid was calculated by computing the arithmetic mean of the individual MDS1 values. Pairwise Euclidean distances between population centroids were computed in one dimension (MDS1) using the absolute difference between the centroids. The final pairwise Euclidean distance matrix was visualized as a table. Using the gridExtra package in R, the formatted distance matrix was converted into a table with a custom theme.

### SPRUCE

To estimate and identify potential environmental factors contributing to gene flow, we used SPRUCE (*92*), a machine learning approach to integrate genetic and environmental data, to determine which factors may be influencing the population structure in individuals from Nama, Khomani, and Khoe-San descendant communities. SPRUCE uses the MAPS output to extract environmental values from the coordinates corresponding to the produced demes.

MAPS (*42*) estimates time-resolved dispersal rates and population densities on the basis of the number of shared long Pairwise Coalescent Segments. Before calling IBD segments, we performed phasing with SHAPEIT4 (*80*) and removed first- and second-degree relatives using the output from PONDEROSA (see “Identifying relatives” and “Phasing” sections) (*93*). IBD segments were then called using hap-ibd version 1.0 (*94*) and were repaired with the program merge-ibd-segments. For running MAPS, an IBD matrix was constructed using code provided by the SPRUCE authors, and the IBD calls were split into two groups: 2 to 6 cM and greater than 6 cM. The categories were chosen on the basis of historical dates corresponding to pre- and post-European contact in South Africa. The mean ages of the categories are 56.25 and 12.50 generations (*66*). Following the recommendation of the SPRUCE authors, we chose to use 300 demes for higher-resolution migration patterns. Migration surfaces were visualized using the R package plotmaps (*42*).

Climate data such as mean annual temperature, maximum temperature of the hottest month, minimum temperature of the coldest month, mean annual precipitation, precipitation in the wettest month, and precipitation in the driest month were sourced from the free, online repository CHELSA (Climatologies at High Resolution for Earth’s Land Surface Areas) (*95*). Slope data were obtained from the Geomorpho90m dataset (*96*), and elevation was obtained from MERIT DEM (Multi-Error-Removed Improved Terrain Digital Elevation Models) (*97*). Processing steps were performed using the Geospatial Data Abstraction Library (GDAL/OGR Contributors 2021) in a Bash environment. The geographic and environmental data were clipped within the geographic range of interest and the demes.txt output file from MAPS software.

The predictor variables for the random forest regression were the values of 10 spatial variables related to climate, ecology, and geography at each deme. All variables were numeric and continuous with the exception of lakes and rivers, which were coded as “1” for a body of water being present and “0” for a body of water being not present. The response variable was calculated by using the migration rate at each deme from the “mRates.txt” file outputted by MAPS and converting them to 10*^m^* (*92*). The R package randomForestSRC was used to perform the random forest regressions (https://CRAN.R-project.org/package=randomForestSRC). We performed random forest regressions for both 56 to 13 generations ago (IBD segments greater than 6 cM) and less than 13 generations ago (IBD segments between 6 and 2 cM), which were our time intervals of interest. All other model specifications and considerations were applied as described by Wickham (*91*).

### Within- and between-population IBD sharing

GERMLINE2 (*98*) was used to call IBD segments to investigate IBD sharing both within and between communities across South Africa. Phasing was performed using SHAPEIT (see the “Phasing” section). First- and second-degree relatives were removed using PONDEROSA (see the “Identifying relatives” section) and KING IBDseg. IBD segments were repaired using merge-ibd-segments. GERMLINE2 reports all pairwise IBD segments for each chromosome. We filtered for IBD segments greater than 6 cM and calculated the sum of the IBD segment lengths for each pair of individuals to get the total length of pairwise shared IBD across the genome. We then filtered the total length of pairwise shared IBD for segments greater than 13 cM. A heatmap analyzing within- and between-community IBD sharing was generated in R using pheatmap (version 1.0.12). The mean of the total shared IBD segments was calculated for the heatmap to look at the average shared IBD within and between communities. IBD networks were generated in R using iGraph.

## Supplementary Materials

### This PDF file includes:

Supplementary Text Tables S1 to S7 Figs. S1 to S27 References
