# Supplementary material for: Recovering the precolonial population structure of Khoe-San descendant populations
Source: Sci Adv. 2026 Sep 2;12(36):eadz7115. doi: 10.1126/sciadv.adz7115 (PMC13537254; doi:10.1126/sciadv.adz7115)
Supplement: Supplementary file 1 [file sciadv.adz7115_sm.pdf]

Science Advances
AAAS

# Supplementary Materials for

## Recovering the precolonial population structure of Khoe-San descendant populations

Stacy L. Edington *et al.*

Corresponding author: Brenna M. Henn, bmhenn@ucdavis.edu; Austin W. Reynolds, austin.reynolds@unthsc.edu

*Sci. Adv.* **12**, eadz7115 (2026)
DOI: 10.1126/sciadv.adz7115

**This PDF file includes:**

Supplementary Text
Tables S1 to S7
Figs. S1 to S27
References

## Supplemental Tables

| Broad_Anc | Population | Abbreviation | n | References |
|---|---|---|---|---|
| Query | Cederberg | CDB | 170 | This Study |
| Query | Karoo | KRO | 171 | This Study |
| Query | Northern Cape TB | NCTB | 183 | This Study |
| Query | MiddleOrangeRiver | ORG | 96 | This Study |
| East African | Amhara | | 22 | AGVP (71) |
| East African | Gumuz | | 23 | AGVP (71) |
| East African | Oromo | | 24 | AGVP (71) |
| East African | Somali | | 23 | AGVP (71) |
| East African | Wolayta | | 24 | AGVP (71) |
| East Asian | HanChinese | | 103 | 1000G(74) |
| East Asian | Vietnamese | | 99 | 1000G(74) |
| European | British | | 91 | 1000G(74) |
| European | Iberian | | 106 | 1000G(74) |
| SoutheastAsian | Mentawai | | 10 | IDGP(73) |
| SoutheastAsian | Nias | | 15 | IDGP(73) |
| SoutheastAsian | Sumatra | | 7 | IDGP(73) |
| SoutheastAsian | Borneo | | 7 | IDGP(73) |
| SoutheastAsian | Java | | 7 | IDGP(73) |
| SoutheastAsian | Bali | | 1 | IDGP(73) |
| SoutheastAsian | Sulawesi | | 12 | IDGP(73) |
| SoutheastAsian | Flores | | 47 | IDGP(73) |
| SoutheastAsian | Tanimbar | | 6 | IDGP(73) |
| SoutheastAsian | Lebata | | 7 | IDGP(73) |
| SoutheastAsian | Kei | | 6 | IDGP(73) |
| SoutheastAsian | Alor | | 5 | IDGP(73) |
| Khoe-San | BotswananSan | | 313 | Crawford et al. (75) |
| Khoe-San | Khomani | | 115 | Martin et al. (76) |
| Khoe-San | Nama | | 92 | Martin et al. (76) |
| South Asian | Bengali | | 86 | 1000G(74) |
| South Asian | Tamil | | 103 | 1000G(74) |
| South Asian | Gujarati | | 102 | 1000G(74) |
| Niger-Congo | Himba | | 123 | Scelza et al. (77) |
| Niger-Congo | Luhya | | 99 | 1000G(74) |
| Niger-Congo | Yoruba | | 108 | 1000G(74) |
| Niger-Congo | Zulu | | 98 | AGVP(71) |

| | | | |
|---|---|---|---|
| Niger-Congo | Botswana_Kalanga | 3 | Fortes-Lima et al. (25) |
| Niger-Congo | Mozambique_Tsonga | 7 | Fortes-Lima et al. (25) |
| Niger-Congo | Mozambique_Chopi | 4 | Fortes-Lima et al. (25) |
| Niger-Congo | Mozambique_Tswa | 4 | Fortes-Lima et al. (25) |
| Niger-Congo | Mozambique_Chuwabu | 3 | Fortes-Lima et al. (25) |
| Niger-Congo | Mozambique_Nhungwe | 3 | Fortes-Lima et al. (25) |
| Niger-Congo | Mozambique_TongaMoz | 3 | Fortes-Lima et al. (25) |
| Niger-Congo | Mozambique_Ronga | 2 | Fortes-Lima et al. (25) |
| Niger-Congo | Mozambique_Makhuwa | 1 | Fortes-Lima et al. (25) |
| Niger-Congo | Mozambique_Sena | 1 | Fortes-Lima et al. (25) |
| Niger-Congo | Namibia_Himba | 76 | Fortes-Lima et al. (25) |
| Niger-Congo | Namibia_Herero | 22 | Fortes-Lima et al. (25) |
| Niger-Congo | Namibia_Wambo | 22 | Fortes-Lima et al. (25) |
| Niger-Congo | SouthAfrica_Zulu | 63 | Fortes-Lima et al. (25) |
| Niger-Congo | SouthAfrica_Tswana | 32 | Fortes-Lima et al. (25) |
| Niger-Congo | SouthAfrica_Xhosa | 31 | Fortes-Lima et al. (25) |
| Niger-Congo | SouthAfrica_Tsonga | 30 | Fortes-Lima et al. (25) |
| Niger-Congo | SouthAfrica_SothoSouthern | 29 | Fortes-Lima et al. (25) |
| Niger-Congo | SouthAfrica_Pedi | 28 | Fortes-Lima et al. (25) |
| Niger-Congo | SouthAfrica_Venda | 24 | Fortes-Lima et al. (25) |
| Niger-Congo | SouthAfrica_Bhaca | 13 | Fortes-Lima et al. (25) |
| Niger-Congo | SouthAfrica_Ndebele | 8 | Fortes-Lima et al. (25) |
| Niger-Congo | SouthAfrica_Sotho | 7 | Fortes-Lima et al. (25) |
| Niger-Congo | SouthAfrica_Swazi | 6 | Fortes-Lima et al. (25) |
| Niger-Congo | SouthAfrica_Hlubi | 3 | Fortes-Lima et al. (25) |
| Niger-Congo | SouthAfrica_Mputhi | 3 | Fortes-Lima et al. (25) |
| Niger-Congo | SouthAfrica_Shona | 1 | Fortes-Lima et al. (25) |
| Niger-Congo | SouthAfrica_Soto | 1 | Fortes-Lima et al. (25) |
| Niger-Congo | SouthAfrica_SothoNorthern | 20 | Fortes-Lima et al. (25) |
| Niger-Congo | Zambia_Lozi | 21 | Fortes-Lima et al. (25) |
| Niger-Congo | Zambia_Chewa | 17 | Fortes-Lima et al. (25) |
| Niger-Congo | Zambia_Bemba | 13 | Fortes-Lima et al. (25) |
| Niger-Congo | Zambia_Tonga | 10 | Fortes-Lima et al. (25) |
| Niger-Congo | Zambia_Ngoni | 8 | Fortes-Lima et al. (25) |
| Niger-Congo | Zambia_Kaonde | 5 | Fortes-Lima et al. (25) |
| Niger-Congo | Zambia_Shona | 4 | Fortes-Lima et al. (25) |
| Niger-Congo | Zambia_Nsenga | 3 | Fortes-Lima et al. (25) |
| Niger-Congo | Zambia_Tumbuka | 3 | Fortes-Lima et al. (25) |

**Table S1)** Sample Size for populations included in analyses, as well as their respective broader ancestry (Broad_Anc) category. Newly collected samples have the broader ancestry “Query”.

| **Pop** | **EAFR** | **EUR** | **SAS** | **IND** | **WBNT** | **EBNT** | **KHS** | **EAS** |
|---|---|---|---|---|---|---|---|---|
| CDB | 0.024 | 0.266 | 0.117 | 0.031 | 0.015 | 0.097 | 0.420 | 0.031 |
| KRO | 0.010 | 0.158 | 0.063 | 0.021 | 0.010 | 0.212 | 0.505 | 0.021 |
| ORG | 0.034 | 0.207 | 0.034 | 0.010 | 0.095 | 0.124 | 0.486 | 0.010 |
| NCTB | 0.020 | 0.199 | 0.051 | 0.013 | 0.034 | 0.175 | 0.494 | 0.014 |
| KHM | 0.015 | 0.066 | 0.013 | 0.003 | 0.006 | 0.075 | 0.819 | 0.004 |
| NAM | 0.065 | 0.160 | 0.017 | 0.002 | 0.056 | 0.031 | 0.668 | 0.001 |
| Crawford | 0.015 | 0.007 | 0.001 | 0.000 | 0.039 | 0.100 | 0.838 | 0.000 |
| Amhara | 0.574 | 0.413 | 0.010 | 0.001 | 0.000 | 0.000 | 0.002 | 0.000 |
| BEB | 0.000 | 0.064 | 0.793 | 0.030 | 0.000 | 0.000 | 0.000 | 0.112 |
| CHB | 0.000 | 0.005 | 0.004 | 0.002 | 0.000 | 0.000 | 0.000 | 0.989 |
| GBR | 0.000 | 0.997 | 0.003 | 0.000 | 0.000 | 0.000 | 0.000 | 0.000 |
| GIH | 0.000 | 0.126 | 0.868 | 0.002 | 0.000 | 0.000 | 0.000 | 0.003 |
| Gumuz | 0.833 | 0.000 | 0.000 | 0.000 | 0.007 | 0.127 | 0.032 | 0.001 |
| HMB | 0.012 | 0.003 | 0.003 | 0.002 | 0.872 | 0.092 | 0.014 | 0.002 |
| IBS | 0.023 | 0.974 | 0.002 | 0.000 | 0.000 | 0.001 | 0.000 | 0.000 |
| IND | 0.001 | 0.001 | 0.011 | 0.617 | 0.000 | 0.000 | 0.000 | 0.369 |
| KHV | 0.000 | 0.001 | 0.006 | 0.086 | 0.000 | 0.000 | 0.000 | 0.907 |
| LWK | 0.248 | 0.008 | 0.002 | 0.002 | 0.075 | 0.640 | 0.022 | 0.002 |
| Oromo | 0.603 | 0.376 | 0.008 | 0.001 | 0.000 | 0.002 | 0.010 | 0.000 |
| STU | 0.001 | 0.044 | 0.908 | 0.029 | 0.000 | 0.000 | 0.000 | 0.018 |
| Somali | 0.693 | 0.292 | 0.001 | 0.000 | 0.001 | 0.012 | 0.000 | 0.000 |
| Wolayta | 0.647 | 0.293 | 0.012 | 0.002 | 0.002 | 0.005 | 0.038 | 0.001 |
| YRI | 0.108 | 0.001 | 0.002 | 0.002 | 0.121 | 0.764 | 0.001 | 0.002 |
| Zulu | 0.011 | 0.007 | 0.002 | 0.001 | 0.010 | 0.787 | 0.181 | 0.002 |

**Table S2)** ADMIXTURE k=8

| Pop | S_SAN | WBNT | EAS | EAFR | IND1 | SAS | N_SAN | EUR | EBNT | IND2 |
|---|---|---|---|---|---|---|---|---|---|---|
| CDB | 0.420 | 0.018 | 0.014 | 0.020 | 0.030 | 0.121 | 0.026 | 0.241 | 0.093 | 0.017 |
| KRO | 0.466 | 0.012 | 0.008 | 0.008 | 0.023 | 0.065 | 0.068 | 0.133 | 0.207 | 0.011 |
| ORG | 0.468 | 0.098 | 0.007 | 0.029 | 0.008 | 0.036 | 0.047 | 0.180 | 0.120 | 0.006 |
| NCTB | 0.431 | 0.036 | 0.006 | 0.017 | 0.014 | 0.053 | 0.090 | 0.177 | 0.169 | 0.007 |
| KHM | 0.600 | 0.006 | 0.002 | 0.011 | 0.003 | 0.013 | 0.255 | 0.042 | 0.066 | 0.002 |
| NAM | 0.696 | 0.057 | 0.002 | 0.054 | 0.001 | 0.019 | 0.022 | 0.119 | 0.030 | 0.001 |
| Crawford | 0.100 | 0.016 | 0.000 | 0.013 | 0.000 | 0.001 | 0.764 | 0.007 | 0.099 | 0.000 |
| Amhara | 0.003 | 0.000 | 0.000 | 0.575 | 0.000 | 0.010 | 0.000 | 0.410 | 0.000 | 0.000 |
| BEB | 0.000 | 0.000 | 0.090 | 0.000 | 0.034 | 0.793 | 0.000 | 0.064 | 0.000 | 0.019 |
| CHB | 0.000 | 0.000 | 0.951 | 0.000 | 0.045 | 0.001 | 0.000 | 0.002 | 0.000 | 0.000 |
| GBR | 0.000 | 0.000 | 0.000 | 0.000 | 0.000 | 0.003 | 0.000 | 0.997 | 0.000 | 0.000 |
| GIH | 0.000 | 0.000 | 0.002 | 0.000 | 0.002 | 0.868 | 0.000 | 0.127 | 0.000 | 0.002 |
| Gumuz | 0.000 | 0.003 | 0.000 | 0.830 | 0.000 | 0.000 | 0.038 | 0.000 | 0.128 | 0.000 |
| HMB | 0.009 | 0.871 | 0.002 | 0.012 | 0.002 | 0.002 | 0.007 | 0.002 | 0.090 | 0.002 |
| IBS | 0.000 | 0.000 | 0.000 | 0.023 | 0.000 | 0.001 | 0.000 | 0.974 | 0.001 | 0.000 |
| IND | 0.000 | 0.000 | 0.077 | 0.000 | 0.459 | 0.012 | 0.000 | 0.001 | 0.000 | 0.450 |
| KHV | 0.000 | 0.000 | 0.651 | 0.000 | 0.335 | 0.008 | 0.000 | 0.001 | 0.000 | 0.005 |
| LWK | 0.008 | 0.068 | 0.001 | 0.253 | 0.002 | 0.002 | 0.016 | 0.011 | 0.638 | 0.002 |
| Oromo | 0.009 | 0.000 | 0.000 | 0.603 | 0.001 | 0.008 | 0.004 | 0.373 | 0.002 | 0.001 |
| STU | 0.000 | 0.000 | 0.013 | 0.001 | 0.013 | 0.908 | 0.000 | 0.044 | 0.000 | 0.022 |
| Somali | 0.001 | 0.001 | 0.000 | 0.696 | 0.000 | 0.001 | 0.000 | 0.289 | 0.012 | 0.000 |
| Wolayta | 0.021 | 0.001 | 0.001 | 0.647 | 0.001 | 0.012 | 0.021 | 0.289 | 0.005 | 0.002 |
| YRI | 0.001 | 0.112 | 0.002 | 0.114 | 0.001 | 0.003 | 0.001 | 0.002 | 0.761 | 0.003 |
| Zulu | 0.167 | 0.008 | 0.001 | 0.013 | 0.001 | 0.001 | 0.023 | 0.004 | 0.780 | 0.001 |

**Table S3)** ADMIXTURE k=10

| | KHM | NAM | CDB | KRO | ORG | NCTB | !Xoo | Jul'hoansi | Zulu |
|---|---|---|---|---|---|---|---|---|---|
| KHM | 0.000 | 0.080 | 0.036 | 0.022 | 0.034 | 0.023 | 0.100 | 0.067 | 0.010 |
| NAM | 0.080 | 0.000 | 0.043 | 0.059 | 0.046 | 0.058 | 0.131 | 0.146 | 0.073 |
| CDB | 0.036 | 0.043 | 0.000 | 0.016 | 0.005 | 0.014 | 0.108 | 0.103 | 0.030 |
| KRO | 0.022 | 0.059 | 0.016 | 0.000 | 0.012 | 0.002 | 0.098 | 0.087 | 0.014 |
| ORG | 0.034 | 0.046 | 0.005 | 0.012 | 0.000 | 0.011 | 0.103 | 0.099 | 0.027 |
| NCTB | 0.023 | 0.058 | 0.014 | 0.002 | 0.011 | 0.000 | 0.100 | 0.088 | 0.015 |
| !Xoo | 0.100 | 0.131 | 0.108 | 0.098 | 0.103 | 0.100 | 0.000 | 0.104 | 0.094 |
| Jul'hoansi | 0.067 | 0.146 | 0.103 | 0.087 | 0.099 | 0.088 | 0.104 | 0.000 | 0.073 |
| Zulu | 0.010 | 0.073 | 0.030 | 0.014 | 0.027 | 0.015 | 0.094 | 0.073 | 0.000 |

**Table S4)** Khoe-San Euclidean distances between population centroids using Khoe-San ancestry-specific MDS1 and MDS2 coordinates.

| | Botswana | Mozambique | Namibia | SouthAfrica | Zambia | CDB | KRO | NCTB |
|---|---|---|---|---|---|---|---|---|
| **Botswana** | 0.000 | 0.012 | 0.066 | 0.042 | 0.019 | 0.007 | 0.023 | 0.021 |
| **Mozambique** | 0.012 | 0.000 | 0.071 | 0.031 | 0.030 | 0.011 | 0.014 | 0.017 |
| **Namibia** | 0.066 | 0.071 | 0.000 | 0.074 | 0.073 | 0.061 | 0.065 | 0.057 |
| **SouthAfrica** | 0.042 | 0.031 | 0.074 | 0.000 | 0.060 | 0.037 | 0.020 | 0.026 |
| **Zambia** | 0.019 | 0.030 | 0.073 | 0.060 | 0.000 | 0.025 | 0.041 | 0.039 |
| **CDB** | 0.007 | 0.011 | 0.061 | 0.037 | 0.025 | 0.000 | 0.017 | 0.014 |
| **KRO** | 0.023 | 0.014 | 0.065 | 0.020 | 0.041 | 0.017 | 0.000 | 0.009 |
| **NCTB** | 0.021 | 0.017 | 0.057 | 0.026 | 0.039 | 0.014 | 0.009 | 0.000 |
| **ORG** | 0.033 | 0.035 | 0.037 | 0.039 | 0.047 | 0.027 | 0.027 | 0.019 |

**Table S5)** Non-Khoe-San African Euclidean distances between population centroids using non-Khoe-San African ancestry-specific MDS1 and MDS2 coordinates.

| | KHM | NAM | CDB | KRO | ORG | NCTB | FIN | French | GBR | IBS | TSI |
|---|---|---|---|---|---|---|---|---|---|---|---|
| KHM | 0.000 | 0.005 | 0.003 | 0.004 | 0.002 | 0.003 | 0.066 | 0.011 | 0.042 | 0.020 | 0.052 |
| NAM | 0.005 | 0.000 | 0.003 | 0.003 | 0.003 | 0.003 | 0.070 | 0.006 | 0.037 | 0.020 | 0.055 |
| CDB | 0.003 | 0.003 | 0.000 | 0.001 | 0.002 | 0.000 | 0.067 | 0.009 | 0.038 | 0.021 | 0.055 |
| KRO | 0.004 | 0.003 | 0.001 | 0.000 | 0.002 | 0.001 | 0.067 | 0.008 | 0.037 | 0.022 | 0.056 |
| ORG | 0.002 | 0.003 | 0.002 | 0.002 | 0.000 | 0.001 | 0.067 | 0.009 | 0.040 | 0.020 | 0.054 |
| NCTB | 0.003 | 0.003 | 0.000 | 0.001 | 0.001 | 0.000 | 0.067 | 0.009 | 0.039 | 0.021 | 0.055 |
| FIN | 0.066 | 0.070 | 0.067 | 0.067 | 0.067 | 0.067 | 0.000 | 0.075 | 0.088 | 0.082 | 0.088 |
| French | 0.011 | 0.006 | 0.009 | 0.008 | 0.009 | 0.009 | 0.075 | 0.000 | 0.034 | 0.019 | 0.058 |
| GBR | 0.042 | 0.037 | 0.038 | 0.037 | 0.040 | 0.039 | 0.088 | 0.034 | 0.000 | 0.052 | 0.092 |
| IBS | 0.020 | 0.020 | 0.021 | 0.022 | 0.020 | 0.021 | 0.082 | 0.019 | 0.052 | 0.000 | 0.040 |
| TSI | 0.052 | 0.055 | 0.055 | 0.056 | 0.054 | 0.055 | 0.088 | 0.058 | 0.092 | 0.040 | 0.000 |

**Table S6)** European Euclidean distances between population centroids using European ancestry-specific MDS1 and MDS2 coordinates.

| Pop | SAS | EAS | NKA | KHS | EUR |
|---|---|---|---|---|---|
| CDB | 0.118 | 0.056 | 0.116 | 0.432 | 0.278 |
| KRO | 0.066 | 0.038 | 0.214 | 0.518 | 0.165 |
| ORG | 0.036 | 0.018 | 0.227 | 0.502 | 0.217 |
| NCTB | 0.053 | 0.025 | 0.208 | 0.507 | 0.207 |
| KHM | 0.013 | 0.006 | 0.073 | 0.838 | 0.070 |
| NAM | 0.019 | 0.004 | 0.099 | 0.699 | 0.180 |
| Crawford | 0.002 | 0.001 | 0.129 | 0.859 | 0.009 |
| Amhara | 0.033 | 0.014 | 0.395 | 0.040 | 0.517 |
| BEB | 0.753 | 0.136 | 0.000 | 0.000 | 0.111 |
| CHB | 0.001 | 0.992 | 0.000 | 0.000 | 0.007 |
| GBR | 0.002 | 0.000 | 0.000 | 0.000 | 0.998 |
| GIH | 0.818 | 0.004 | 0.000 | 0.000 | 0.178 |
| Gumuz | 0.032 | 0.036 | 0.742 | 0.084 | 0.106 |
| HMB | 0.005 | 0.006 | 0.951 | 0.031 | 0.007 |
| IBS | 0.002 | 0.000 | 0.014 | 0.000 | 0.983 |
| IND | 0.116 | 0.863 | 0.011 | 0.009 | 0.001 |
| KHV | 0.012 | 0.986 | 0.000 | 0.000 | 0.002 |
| LWK | 0.015 | 0.011 | 0.887 | 0.036 | 0.050 |
| Oromo | 0.029 | 0.017 | 0.420 | 0.051 | 0.483 |
| STU | 0.864 | 0.040 | 0.000 | 0.000 | 0.096 |
| Somali | 0.013 | 0.018 | 0.507 | 0.036 | 0.425 |
| Wolayta | 0.035 | 0.020 | 0.460 | 0.081 | 0.404 |
| YRI | 0.015 | 0.008 | 0.956 | 0.005 | 0.016 |
| Zulu | 0.007 | 0.004 | 0.794 | 0.182 | 0.012 |

**Table S7)** ADMIXTURE k=5

## Supplemental Figures

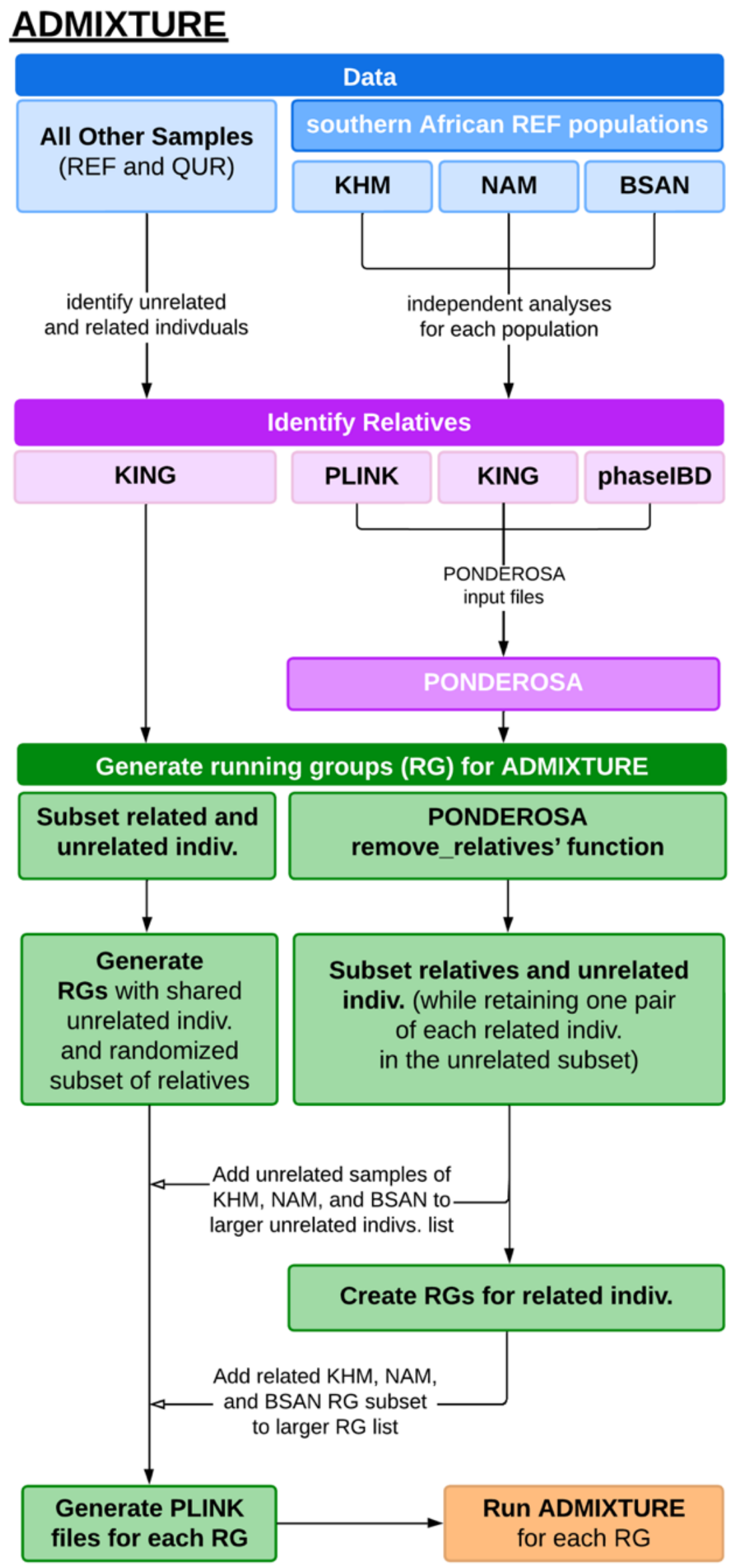


**Figure S1)** Identifying relatives' workflow. Visual of applied workflow used to identify relatives and subset samples into running groups (RG) for ADMIXTURE. Each RG contained the same set of unrelated individuals but a different subset of related individuals. They were all subject to 10 iterations of ADMIXTURE, all with a different initial starting seed and cross-validated five times. PLINK LD pruning was completed prior to sub-setting samples, but after identifying relatives. PLINK LD pruning not depicted in the workflow.

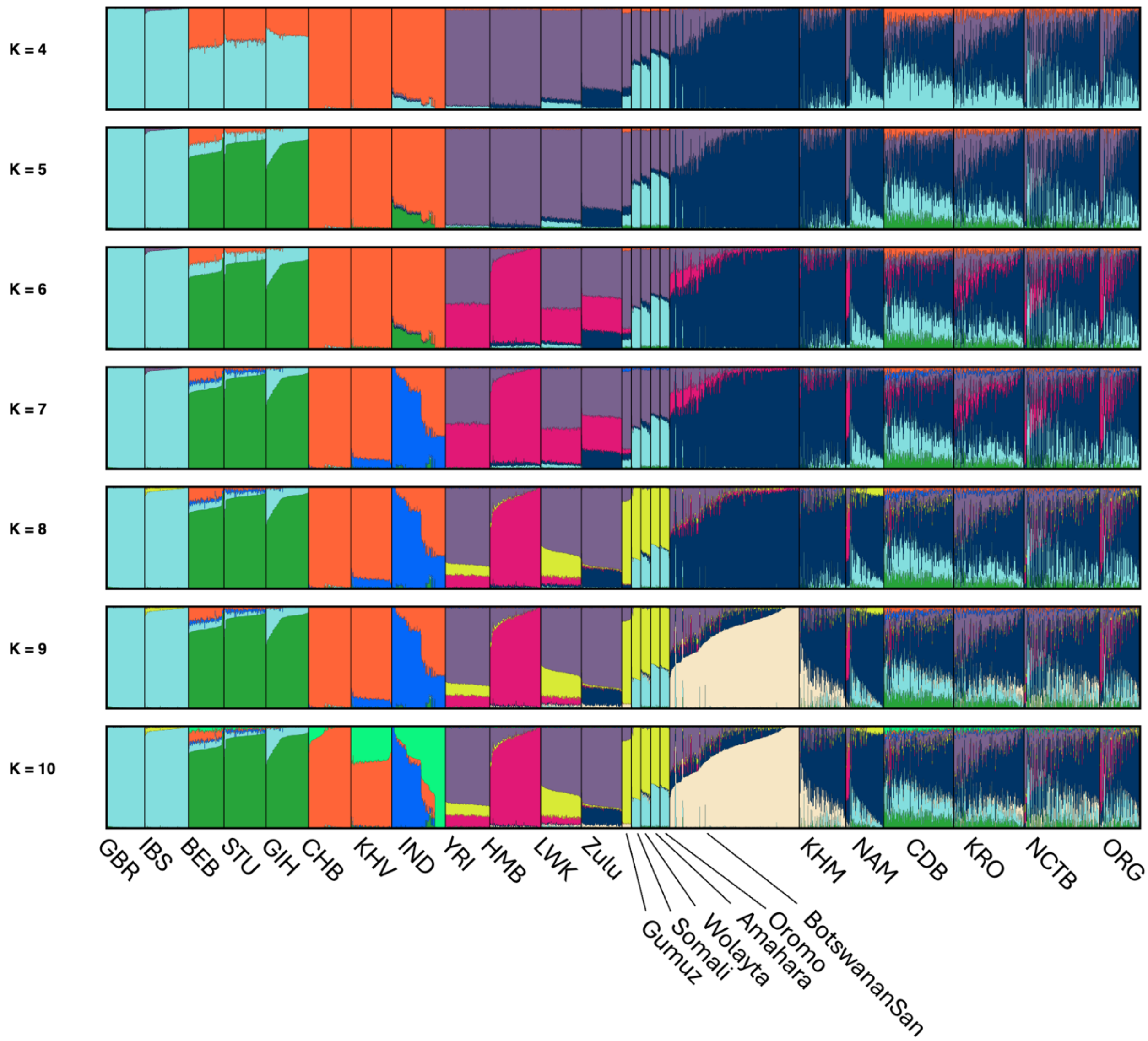


**Figure S2)** Unsupervised ADMIXTURE run of *k*=4 through *k*=10 using final merged dataset of 2,505 individuals and 806,460 markers congruent H3Africa array.

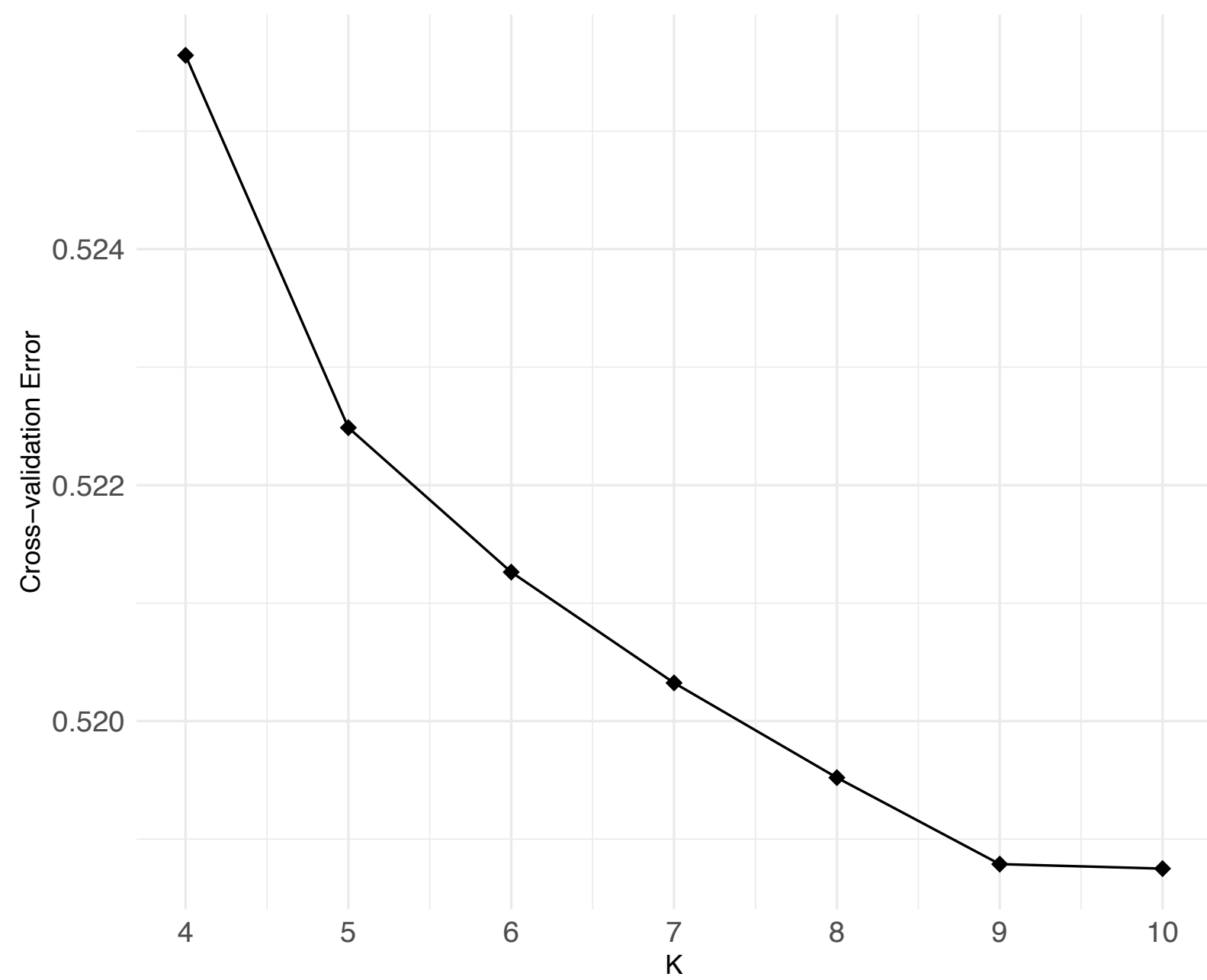


**Figure S3)** Cross-validation error rate for ADMIXTURE visualized in Supplemental Figure 6.

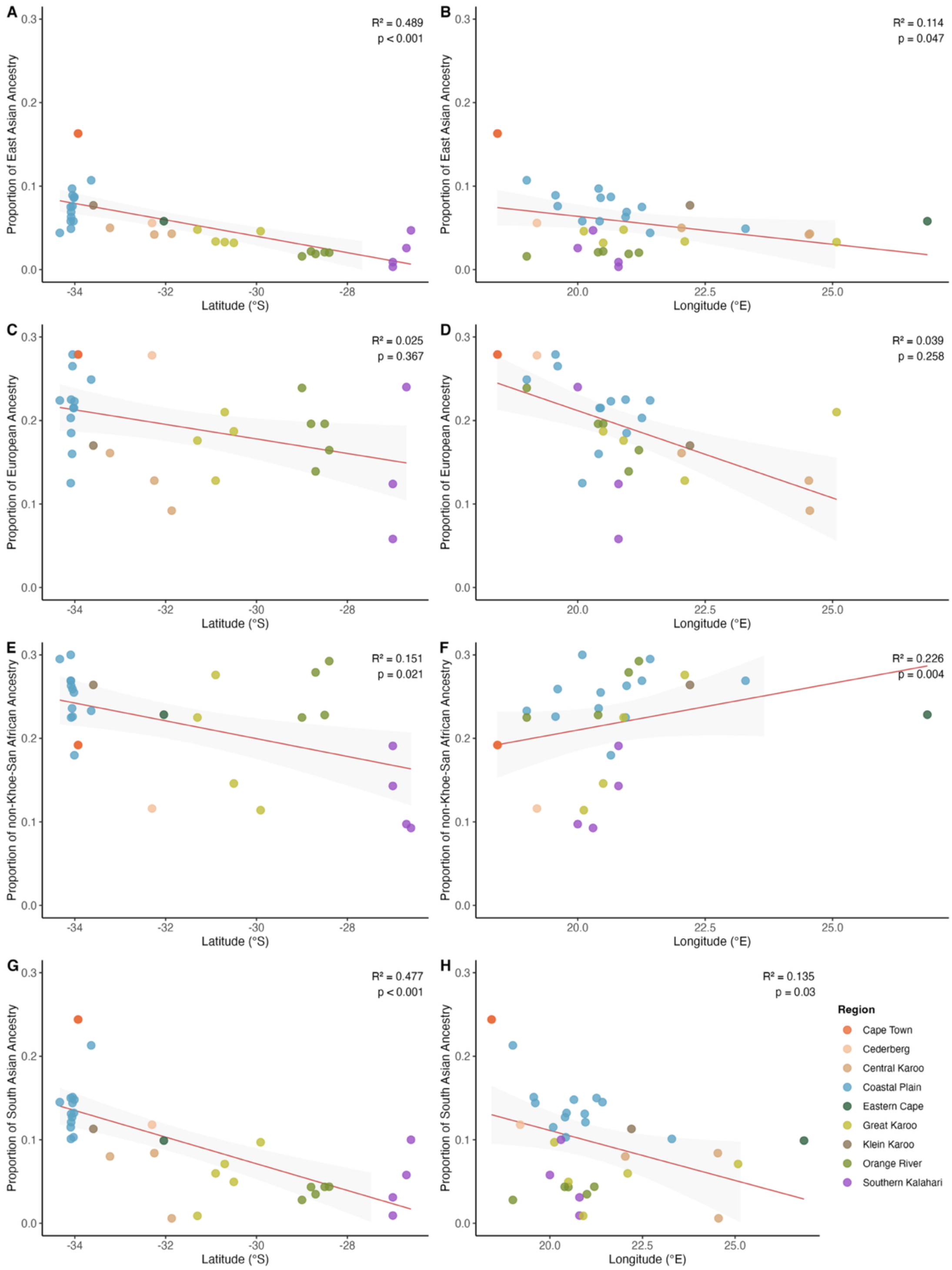


**Figure S4) Linear Regression for ancestry by distance plotted against latitude and longitude**. Points are colored according to sampled geographic region.

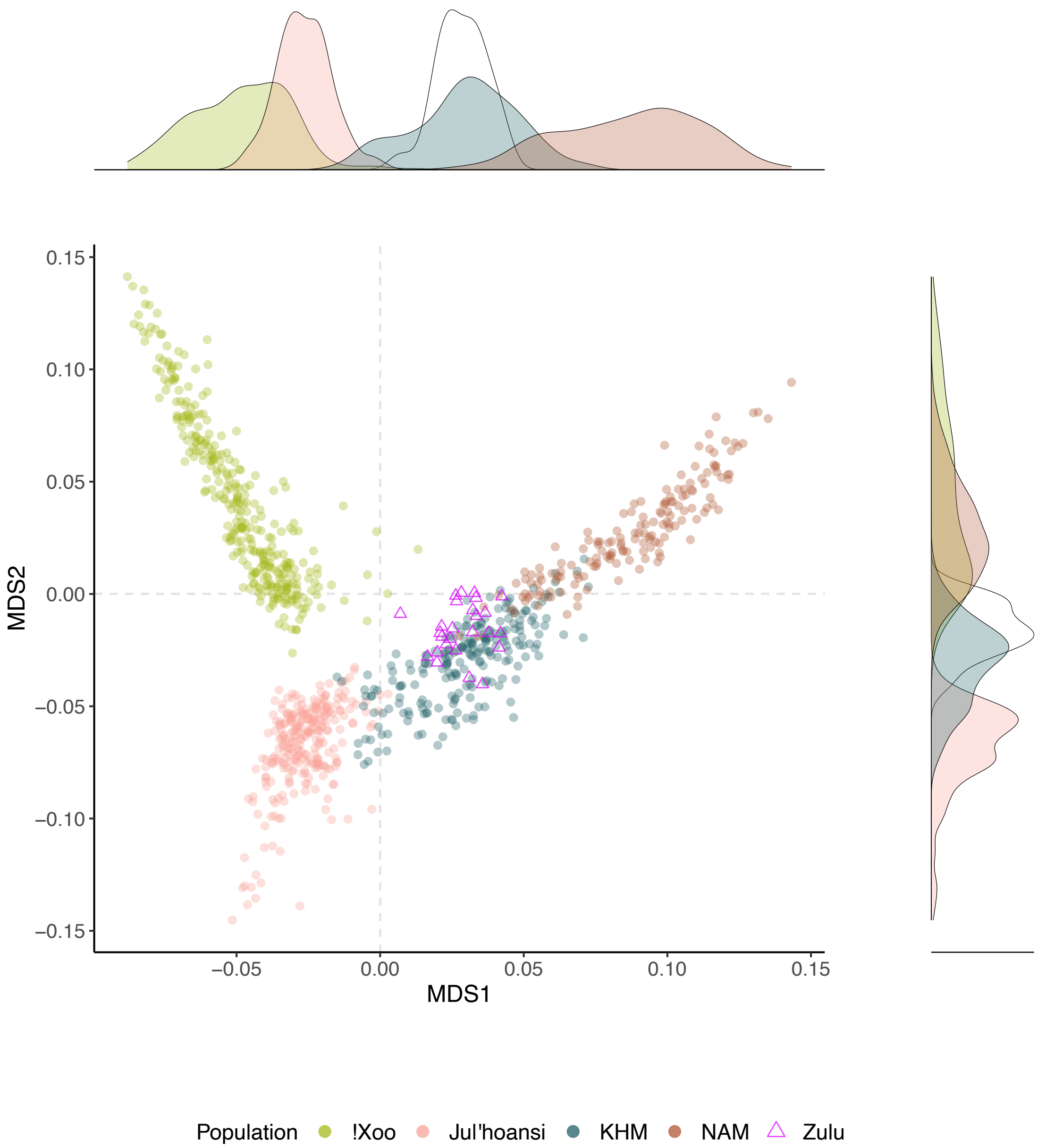


**Figure S5)** Zulu derived Khoe-San asMDS**.**

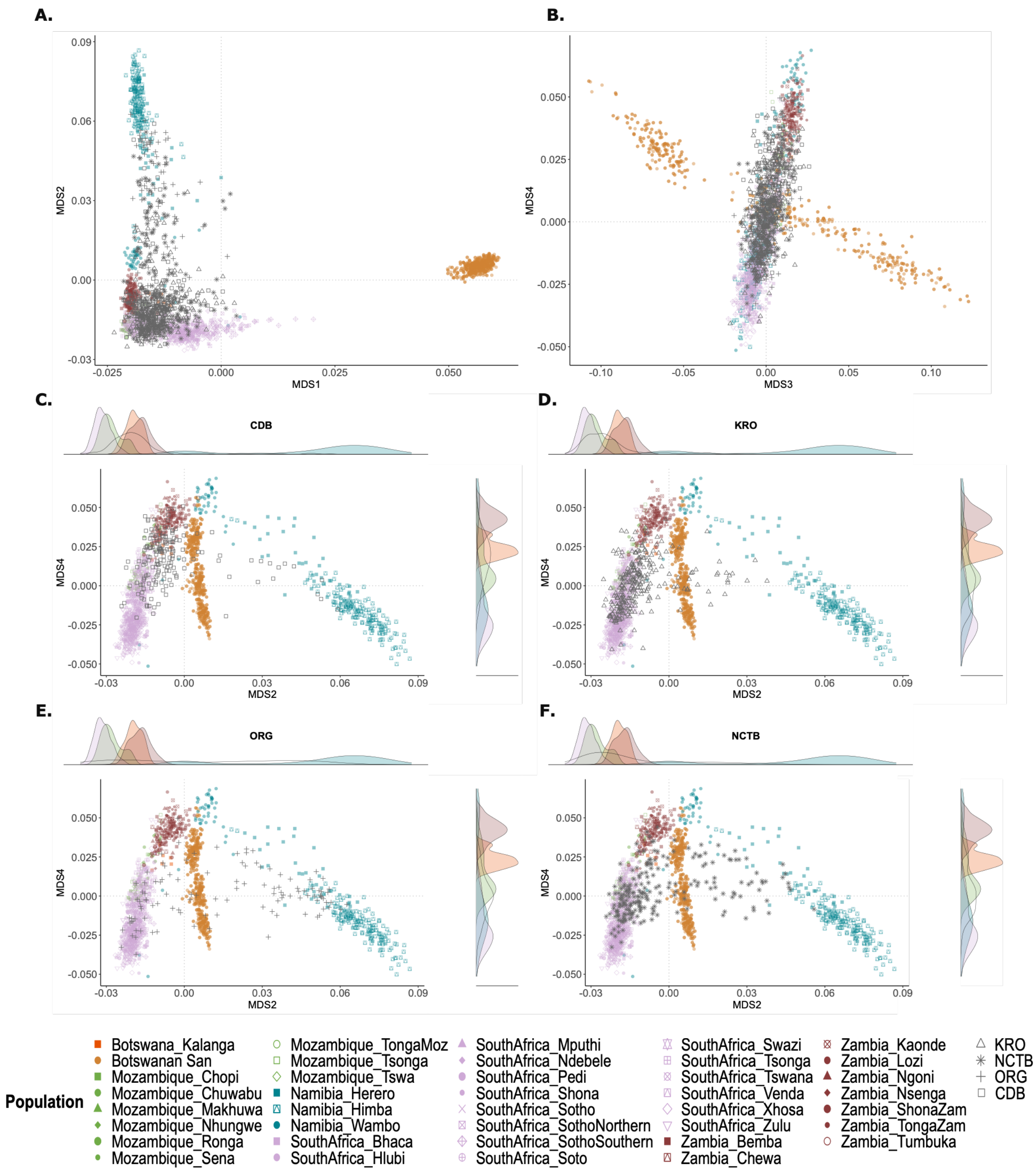


**Figure S6)** Non-Khoe-San African (NKA) asMDS results. The top row (A and B) visualizes results for MDS1 through MDS4. The bottom two rows (C, D, E, and F) visualizes MDS2 and MDS4 by query population, including density plots with the respective query population plotted with a transparent line.

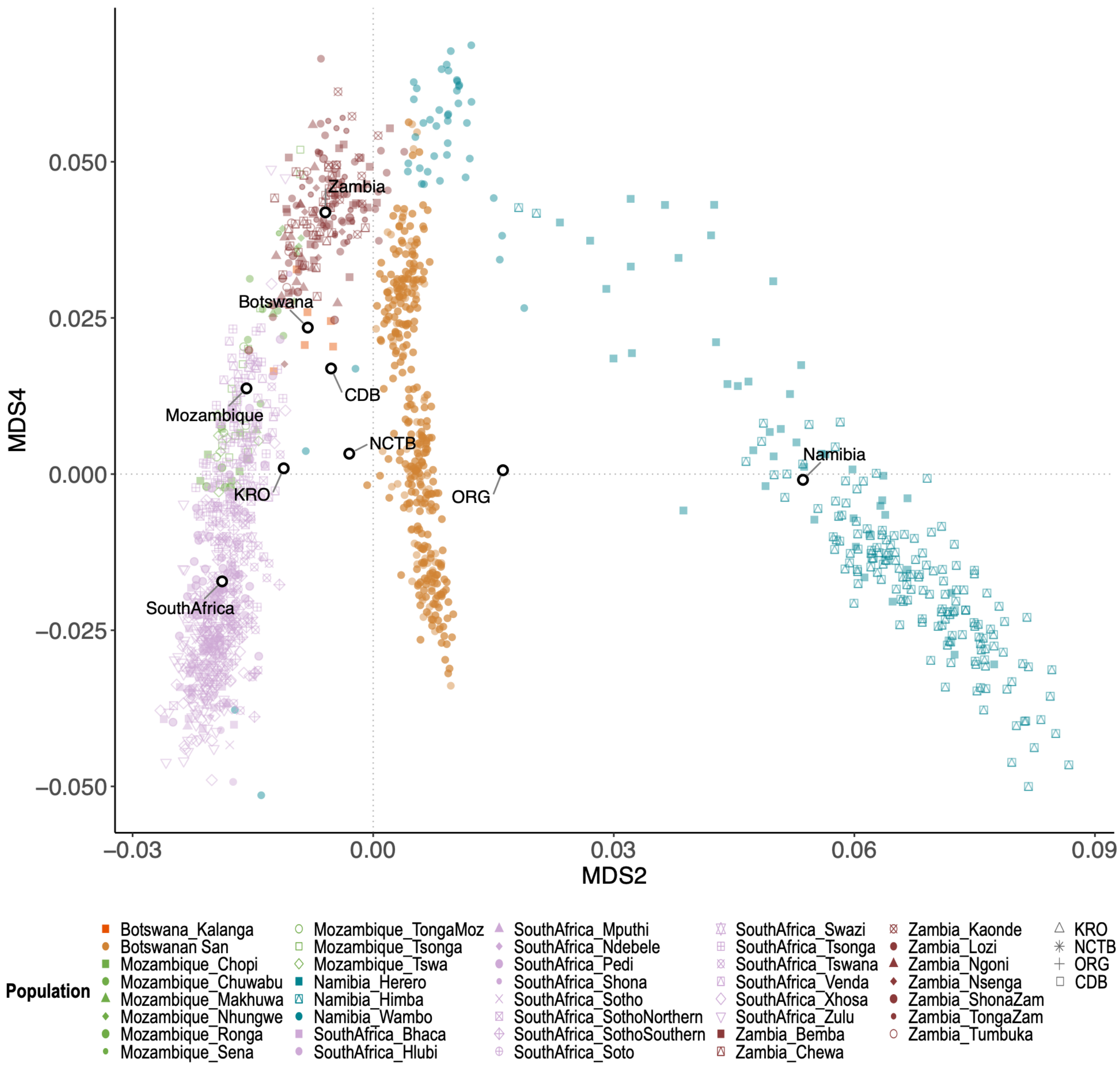


**Figure S7)** Non-Khoe-San derived population centroids plotted against non-Khoe-San reference populations. Query sample haplotypes are not plotted for clearer visualization of reference populations distributions.

**Local ancestry results**

Our model appears well-calibrated, with the confusion matrix showing >96% accuracy in predicting EAS, NKA, and KHS haplotypes, and ~90% accuracy for EUR and SAS segments. Correlation between ADMIXTURE results for 5 population clusters and Gnomix global ancestry results is high for EUR, NKA, and KHS ($r^2 \geq 0.96$), albeit slightly lower for smaller segments like EAS ($r^2 = 0.94$) and SAS ($r^2 = 0.91$), still indicating a strong correlation.

The highest local ancestry misassignment occurs between SAS and EUR ancestries, with 6.9% of SAS segments being assigned to EUR and approximately 3.5% of EUR segments assigned to SAS. This misassignment also contributes to the larger variance in EUR and SAS ancestry probability distributions (Supplemental Figure 11). We attributed this to the 'Indian cline,' referring to the geographical distribution of two primary ancestral groups across South Asia: the 'Ancestral North Indians' (ANI) and the 'Ancestral South Indians' (ASI)[104-107]. The ANI share closer genetic similarities with populations from the Near Eastern and European populations, while the ASI are more closely related to South Asians[104-107], though many populations across South Asia retain a proportion of both ancestries. The ANI component among the BEB, STU, and GIH is visualized in our ADMIXTURE, highlighted in light green.

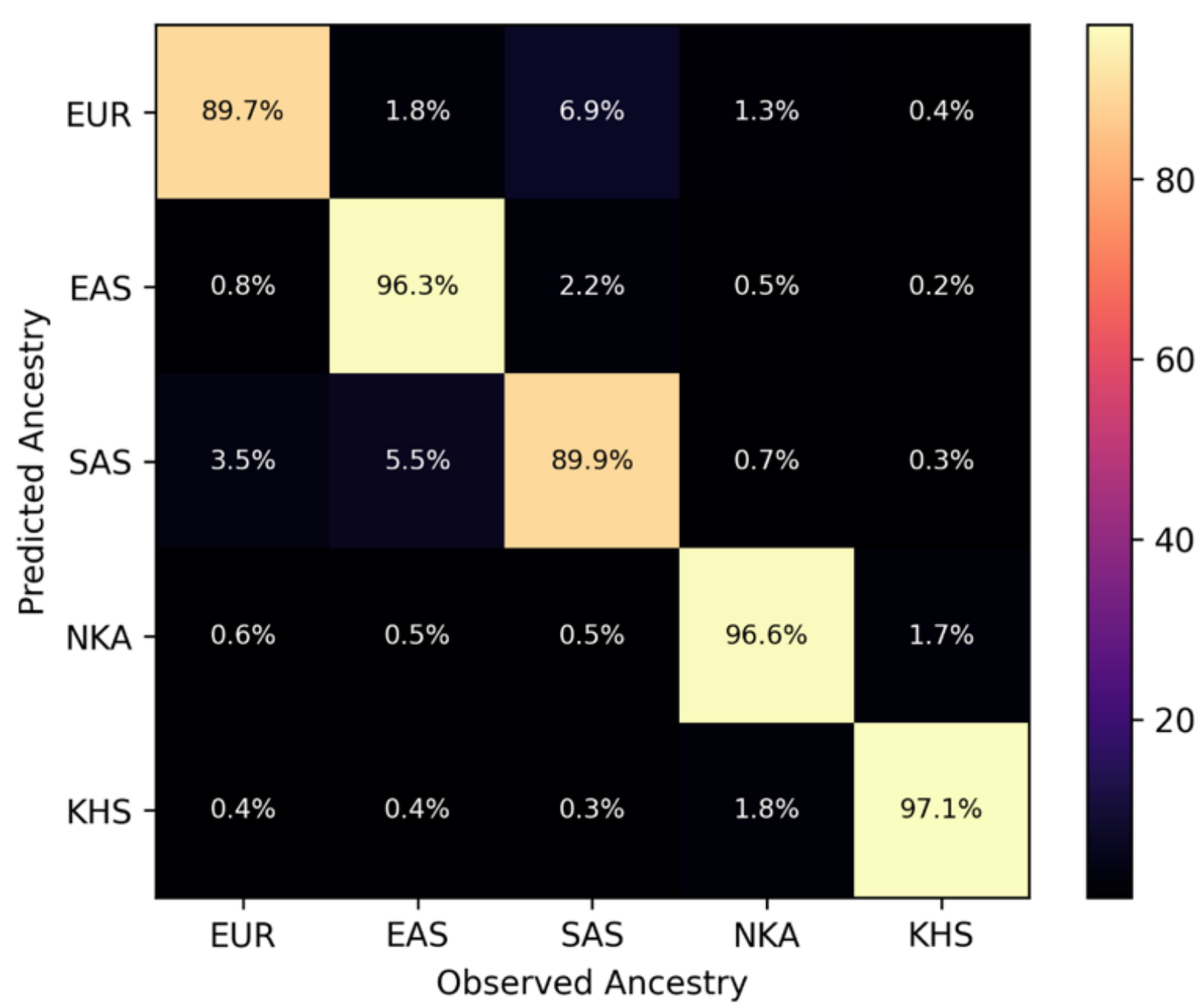


**Figure S8)** Local ancestry inference confusion matrix normalized across all chromosomes, where the x-axis is the ancestry observed, and the y-axis is the model's predicted ancestry. The heat map highlights the accuracy of our model.

| Pop | EAS | EUR | KHS | NKA | SAS |
|---|---|---|---|---|---|
| CDB | 0.052 | 0.236 | 0.447 | 0.128 | 0.137 |
| KRO | 0.039 | 0.137 | 0.517 | 0.234 | 0.073 |
| NCTB | 0.023 | 0.174 | 0.517 | 0.224 | 0.062 |
| ORG | 0.017 | 0.176 | 0.523 | 0.238 | 0.045 |
| KHM | 0.008 | 0.067 | 0.789 | 0.110 | 0.026 |
| NAM | 0.003 | 0.116 | 0.729 | 0.121 | 0.032 |
| BotswananSan | 0.000 | 0.013 | 0.736 | 0.249 | 0.002 |
| Zulu | 0.000 | 0.002 | 0.156 | 0.841 | 0.000 |

**Figure S9)** GNOMIX population-specific global ancestry average proportions.

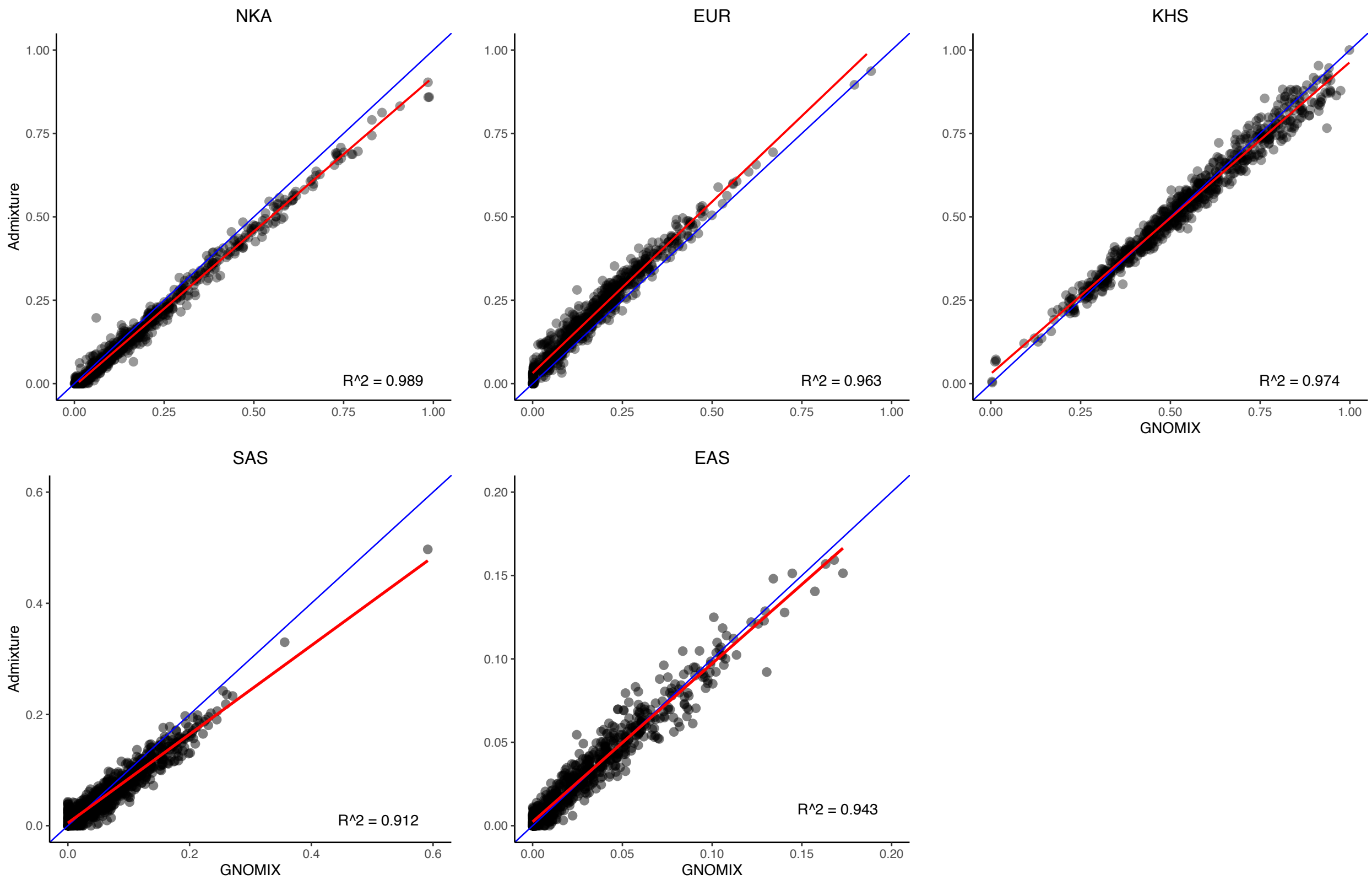


**Figure S10)** Correlation between GNOMIX global ancestry results and ADMIXTURE, where the red line is the best fit and blue line is x=y. R-squared values for each ancestry is reported on the bottom right of the respective plot.

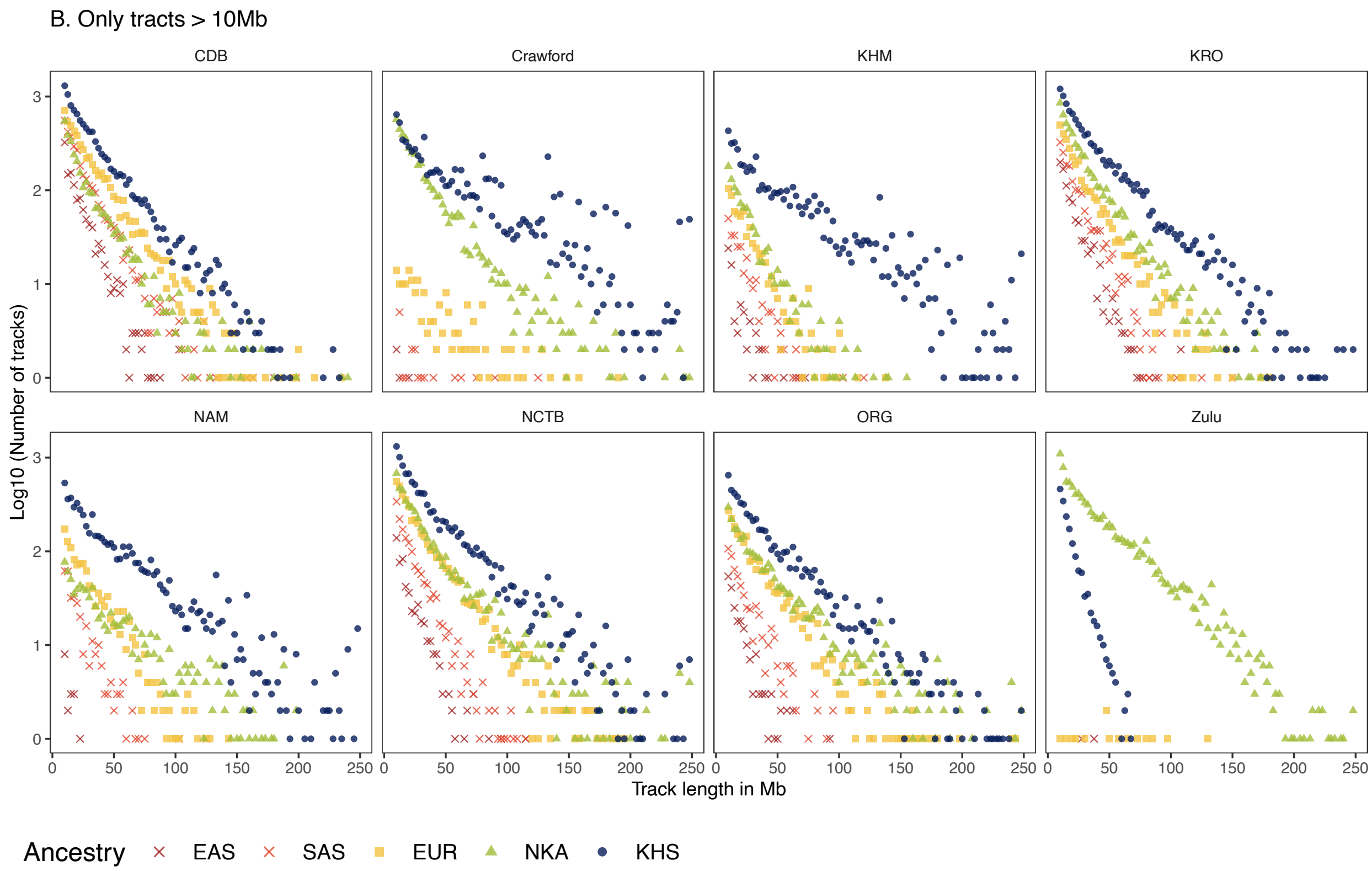


**Figure S11)** Track lengths with at least 75% probability of the respective local ancestry and greater than 10Mb across query populations. The x-axis is the track length in Mb and the y-axis is the log10 of the number of tracks.

## Ancestry-Specific MDS

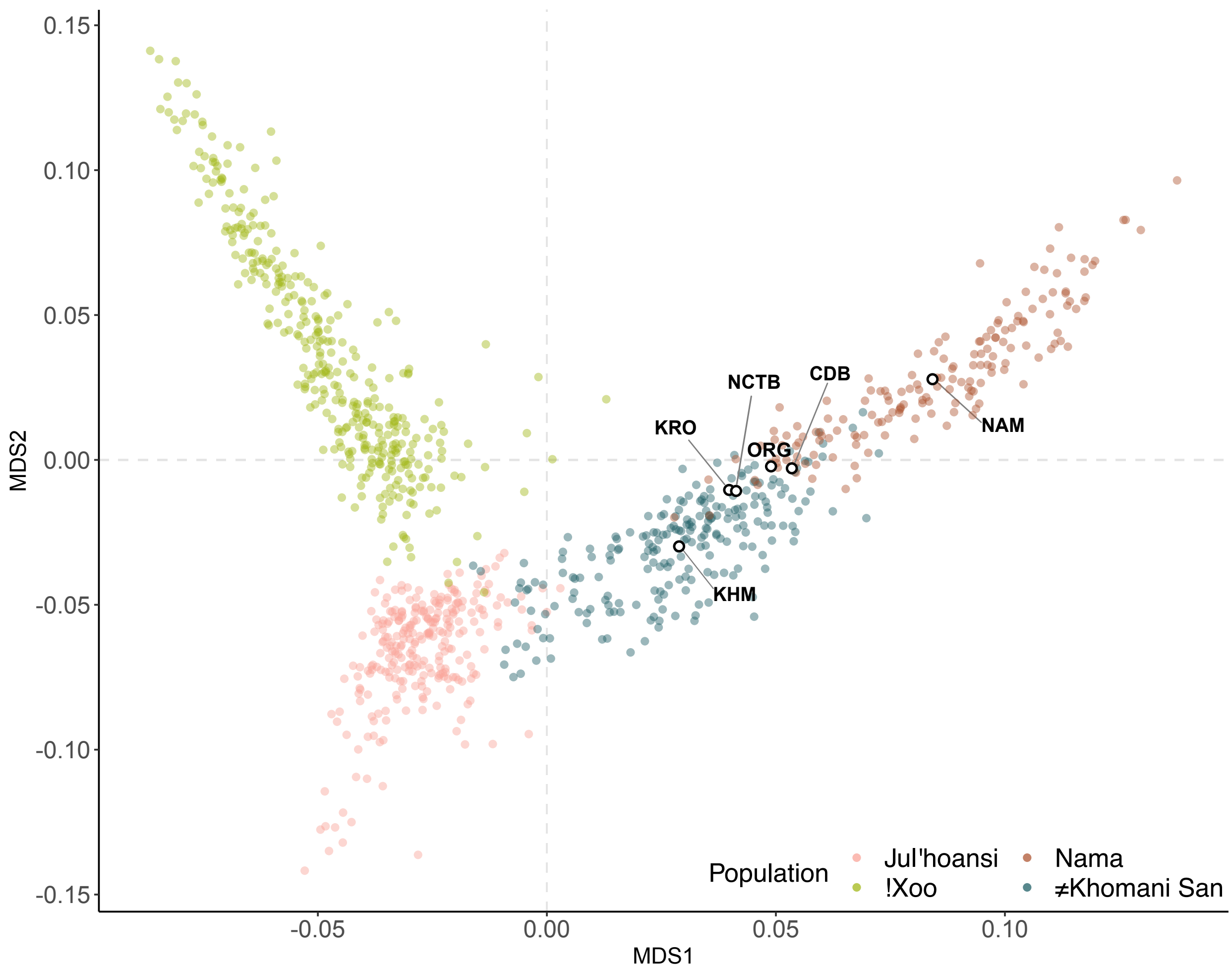


**Figure S12)** European derived centroids plotted against reference populations. Query sample haplotypes are not plotted for clearer visualization of reference populations distributions.

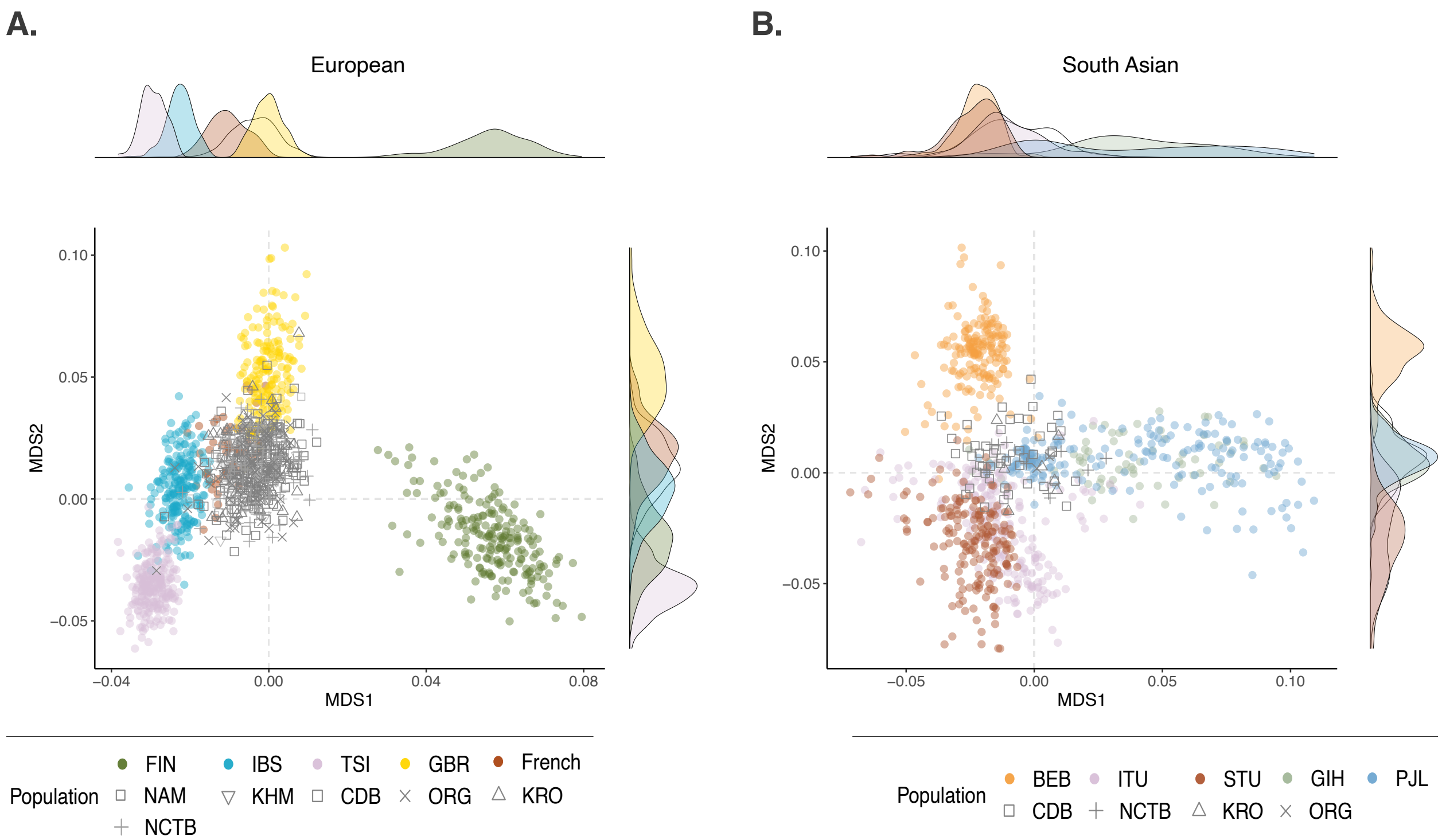


**Figure S13)** European and South Asian asMDS. Local ancestry results were filtered for haplotypes with 75% probability for the respective ancestry and individuals were filtered for at least 20% for the ancestry under investigation.

**SPRUCE**

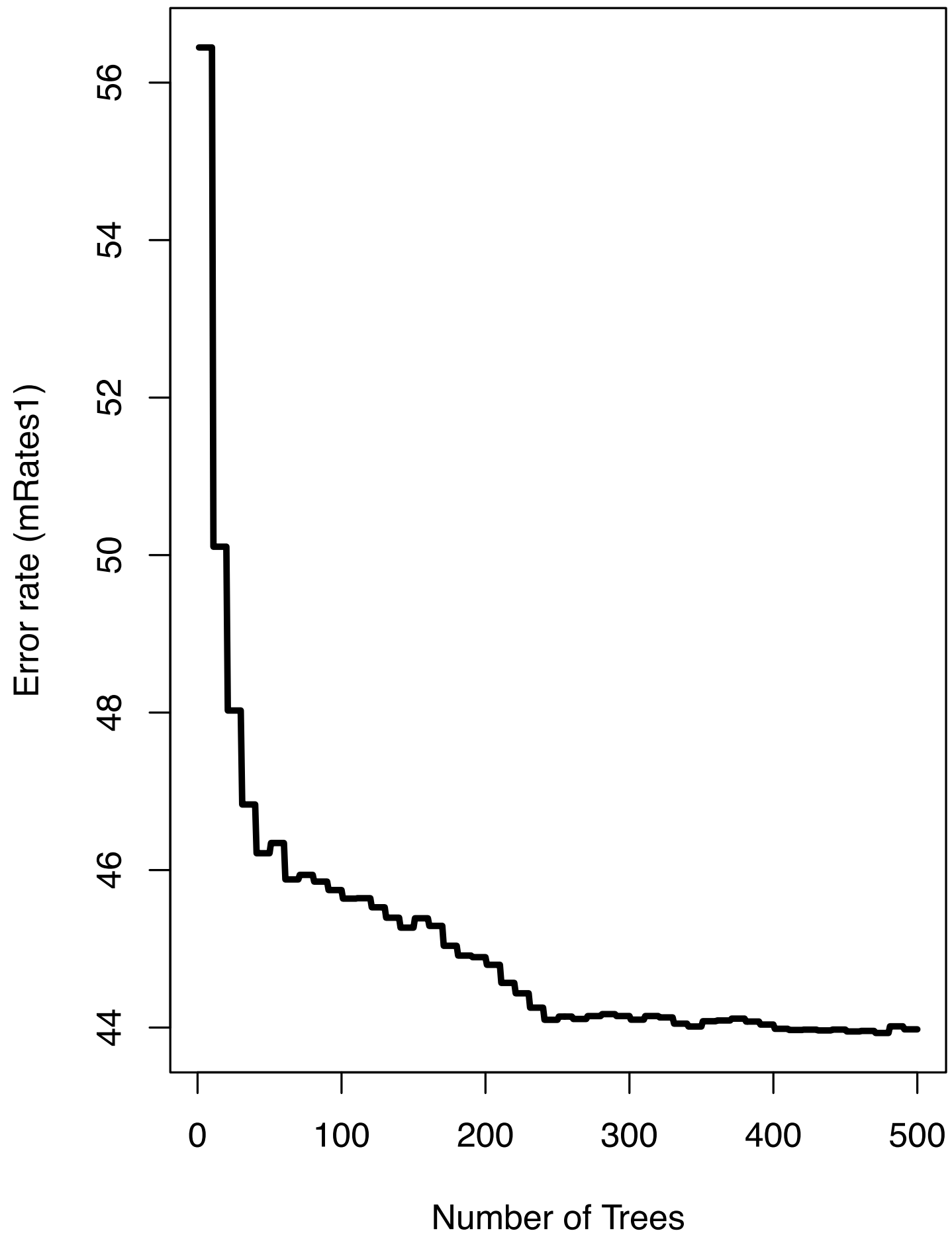


**Figure S14)** Error rate plot showing the error rate decreasing as the number of trees in the random forest increases for IBD segments 2-6 cM representing 56-13 generations ago.

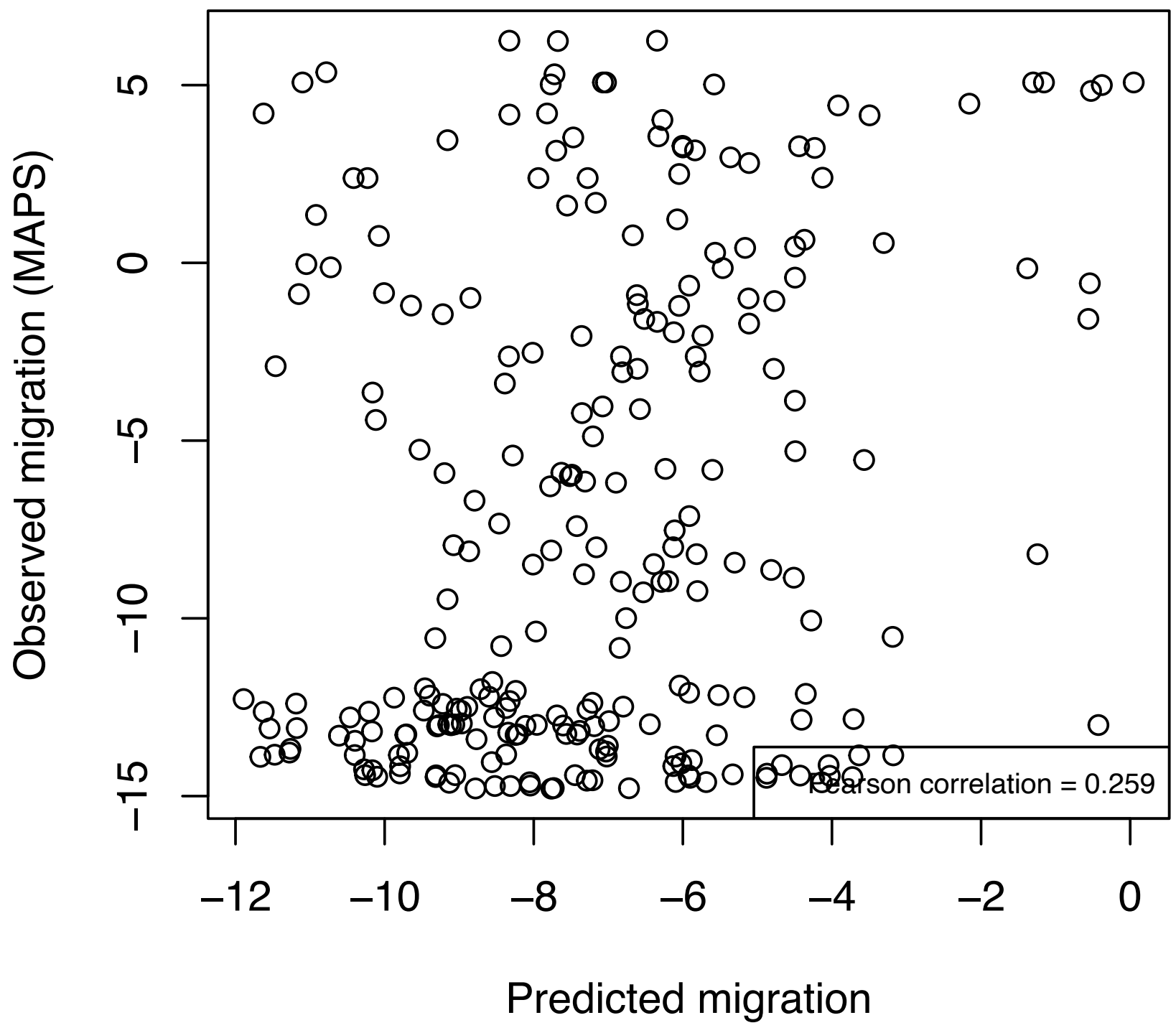


**Figure S15)** Scatterplot showing predicted migration plotted against observed migration (Pearson correlations = 0.259) for IBD segments 2-6 cM/56-13 generations ago.

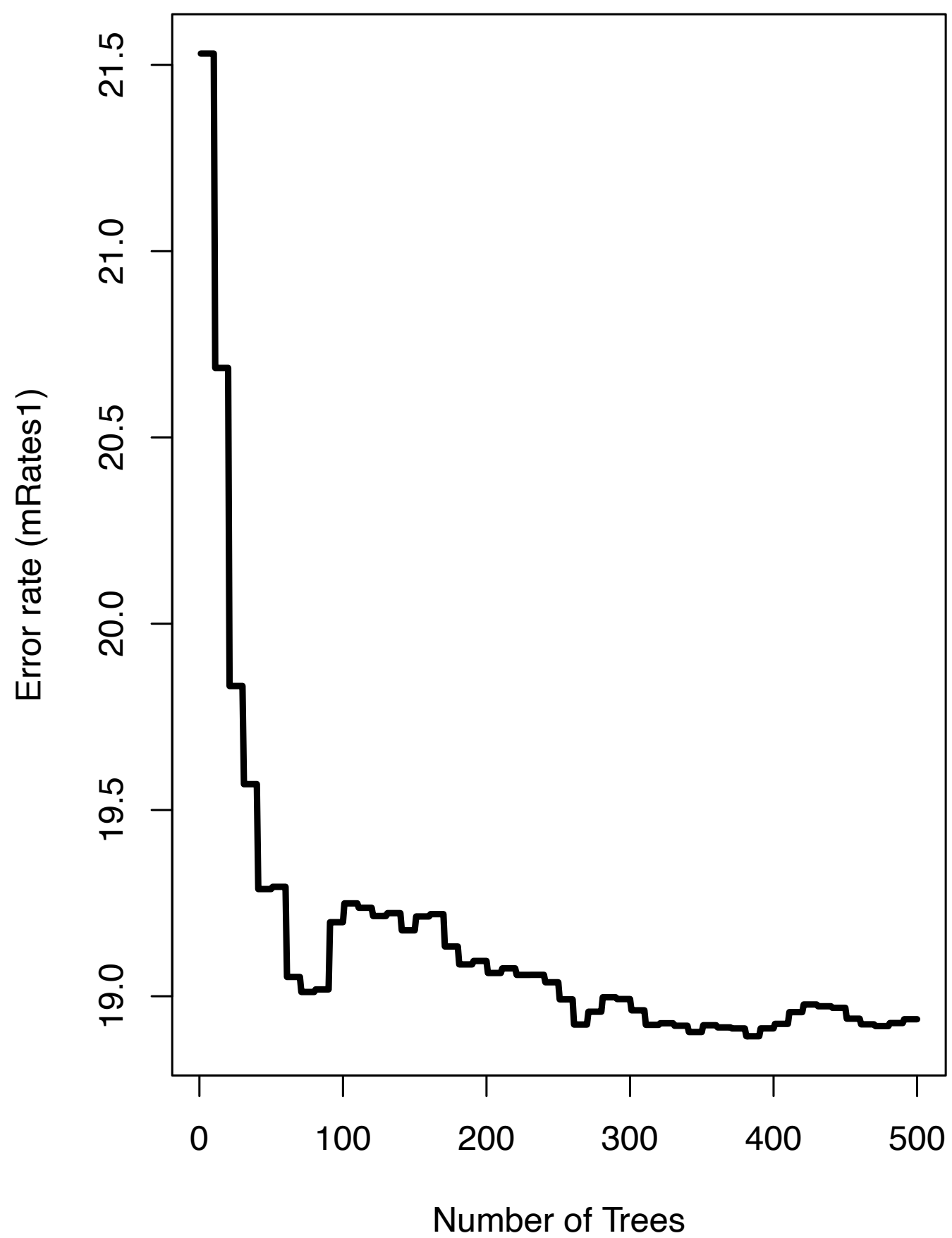


**Figure S16)** Error rate plot showing the error rate decreasing as the number of trees in the random forest increases for IBD segments greater than 6 cM representing less than 13 generations ago.

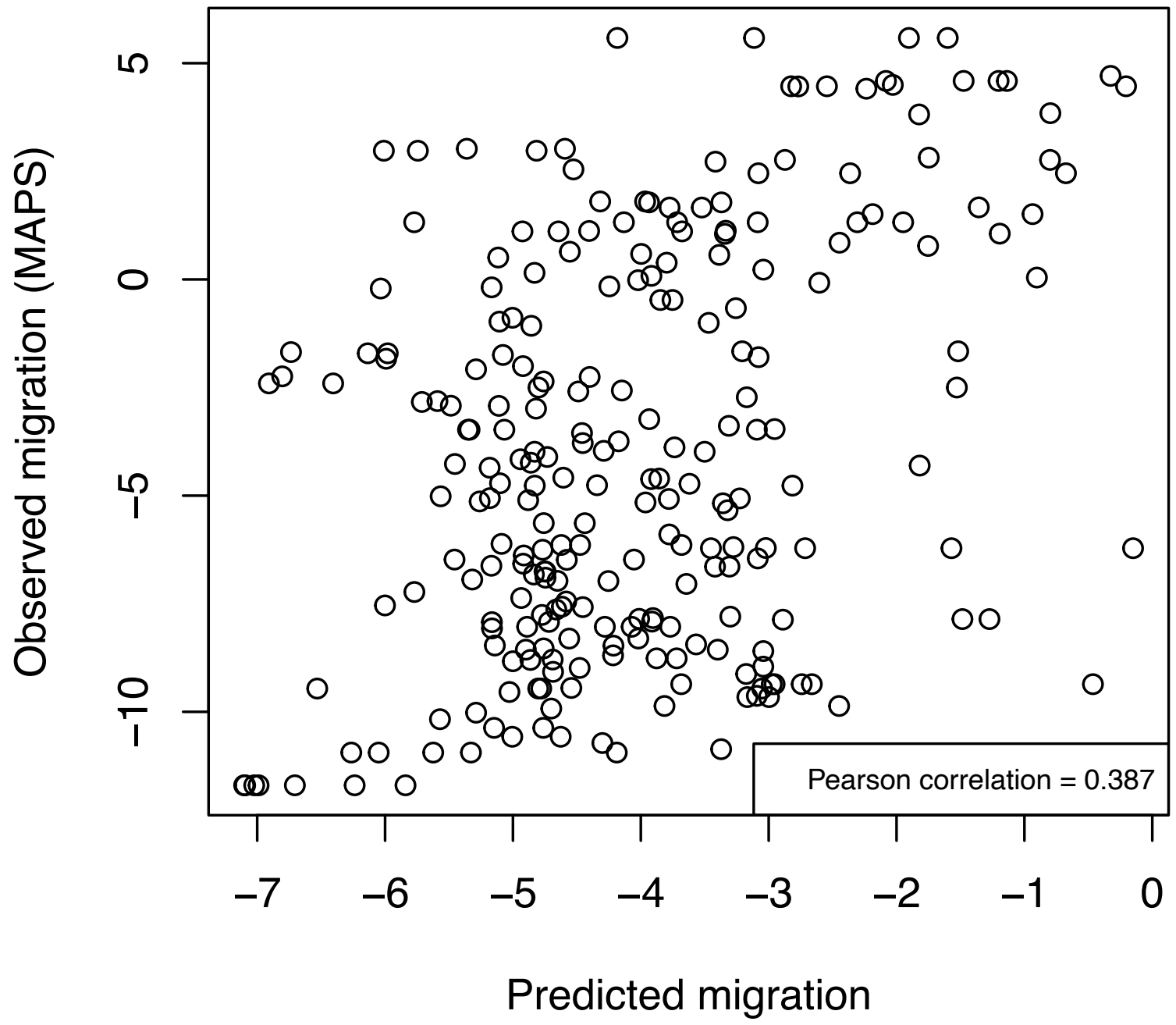


**Figure S17)** Scatterplot showing predicted migration plotted against observed migration (Pearson correlations = 0.387) for IBD segments greater than 6 cM/ less than13 generations ago.

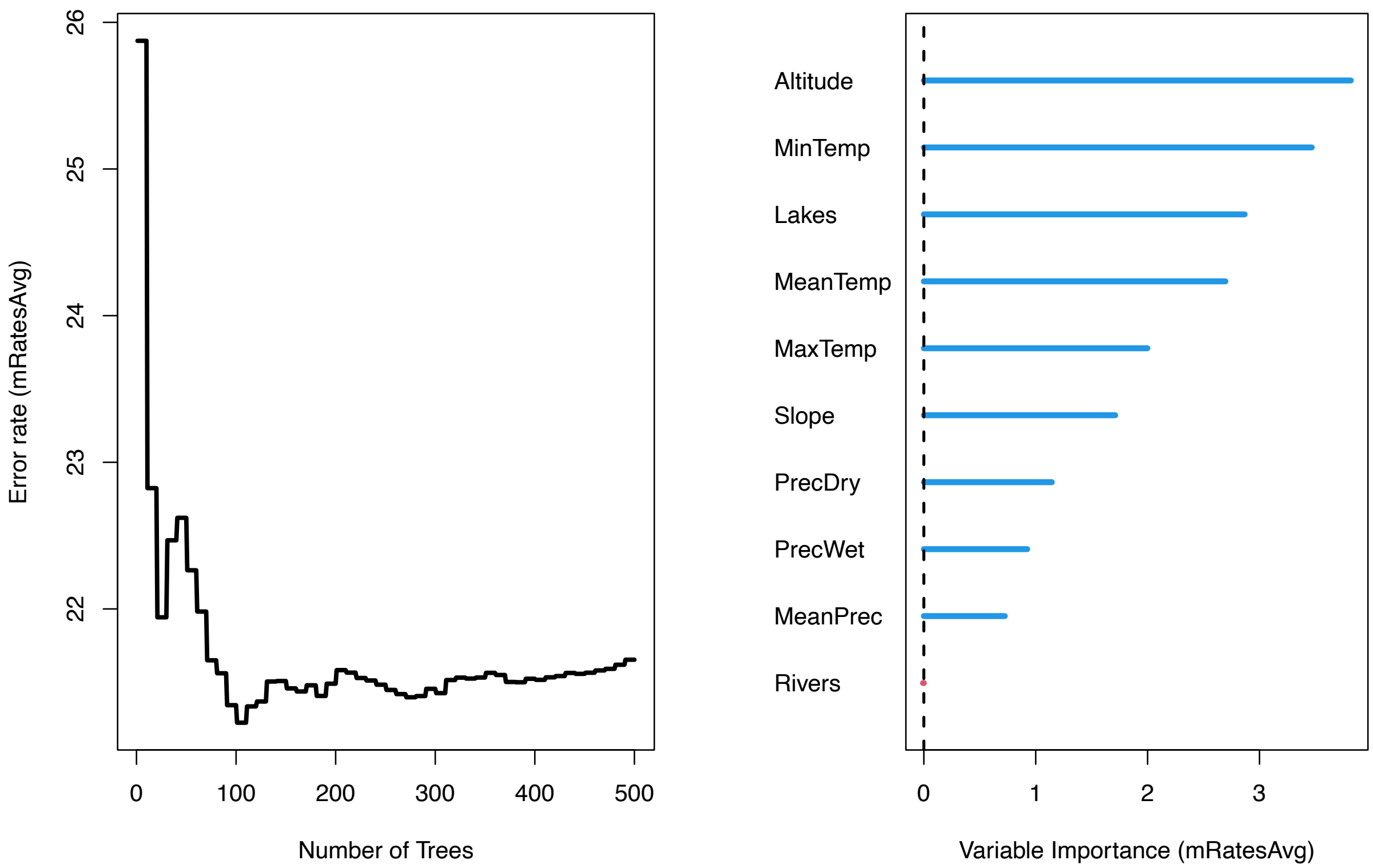


**Figure S18)** SPRUCE Random forest importance table average across time periods (2-6cM and greater than 6cM), 56> generations ago showing altitude and minimum temperature during the coldest month being the most important variables explaining the variation in estimated migration rate.

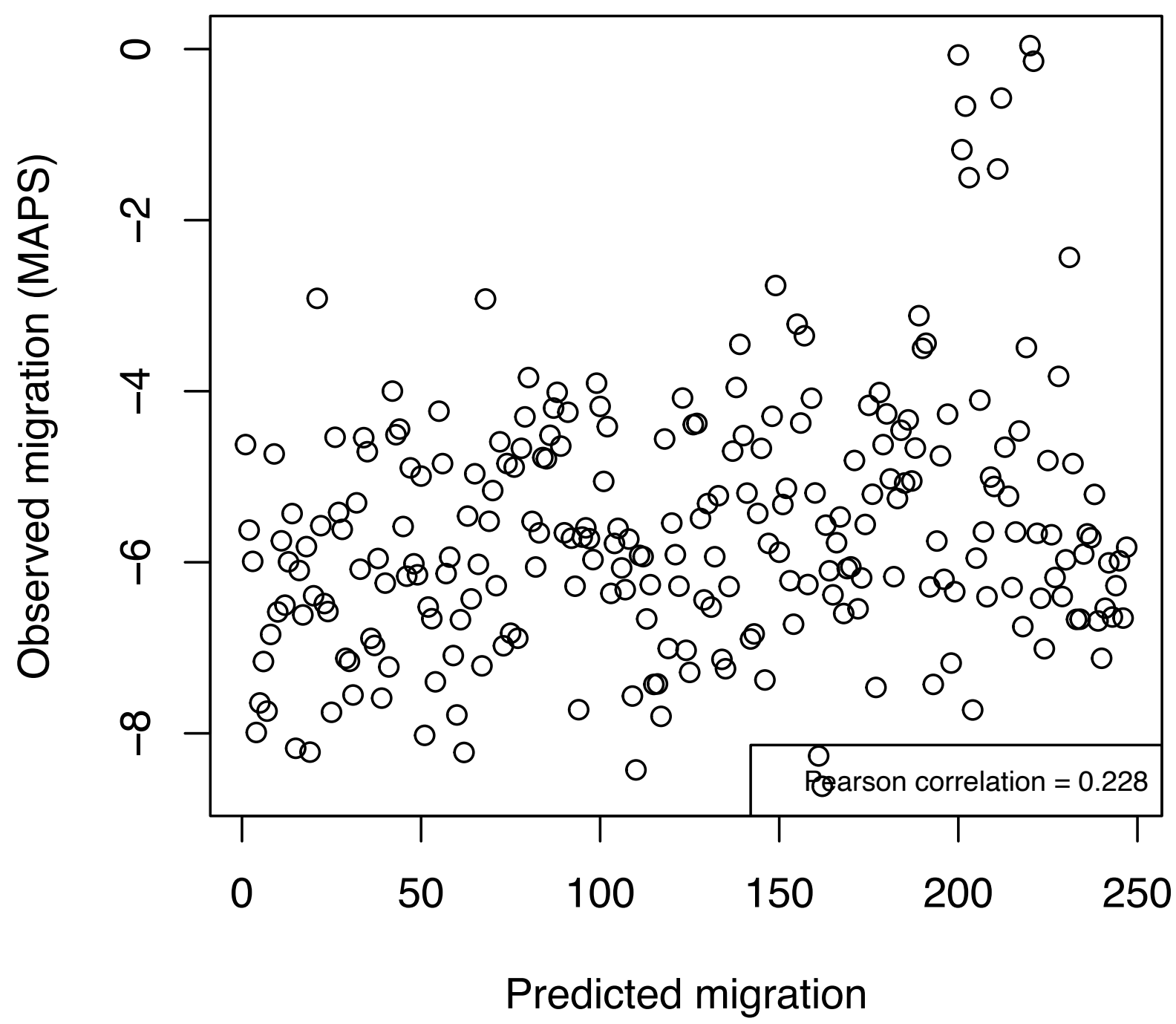


**Figure S19)** Scatterplot showing predicted migration plotted against observed migration (Pearson correlations = 0.228) for the overall model.

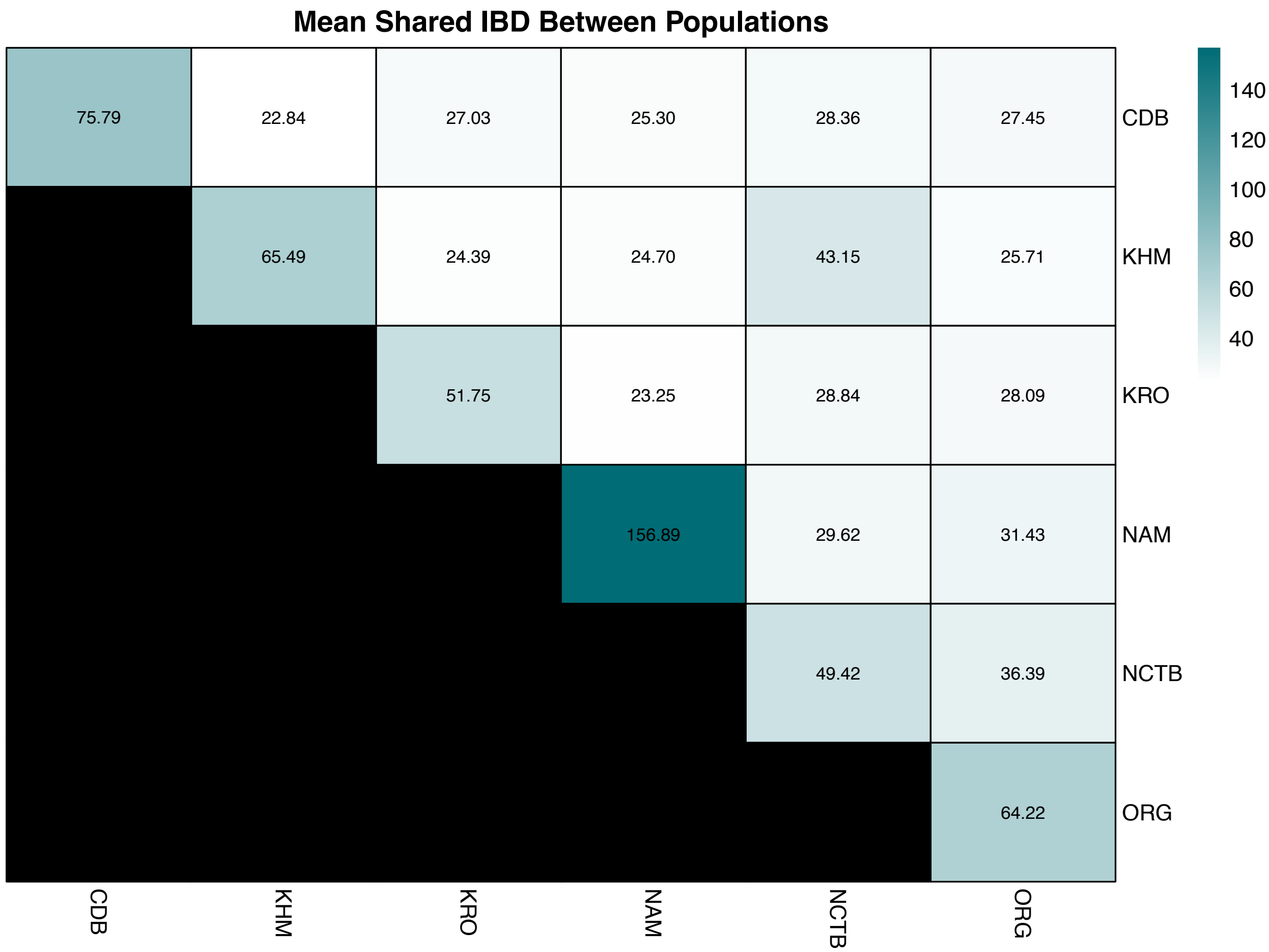


**Figure S20)** Mean shared IBD length between and within Khoe-San and Khoe-San descendant communities.

**Population Centroids and Euclidean Distances**

Supplemental Tables 4-6 and Supplemental Figures 12, 21, and 22.

We aim to best characterize specific source populations for the five primary ancestries within the admixed study populations. We further investigated the relationships among populations by computing the Euclidean distances between each population's centroid (i.e., a population's mean coordinates along MDS1 and MDS2; see *Supplemental Methods*). For instance, the distances from the ≠Khomani San to the Cederberg (CDB), Karoo (KRO), and Orange River (ORG) populations are 0.036, 0.022, and 0.034, respectively, whereas the corresponding distances from NAM to these populations are 0.043, 0.059, and 0.046. These results suggest that, although Cederberg, Karoo, and Orange River are relatively similar in their overall MDS distribution, they share a more similar Khoe-San ancestry profile with ≠Khomani San than with the Nama. The Karoo (0.022) and NCTB (0.021) show the smallest distance to ≠Khomani San, indicating particularly close clustering. Additionally, Zulu haplotypes fall with the ≠Khomani San (0.010), suggesting that the Khoe-San haplotypes present in Zulu individuals are genetically similar to ≠Khomani San.

Among the query populations, we observe generally small distances to Yoruba (YRI), Luhya (LWK), and Zulu, compared to the Himba (HMB). Haplotypes from individuals sampled in the Karoo (0.002) demonstrate close similarities to Yoruba, while the Cederberg centroid appears slightly further from the Yoruba (0.009) and Luhya (0.017) (Supplemental Table 2). Participants sampled along Middle Orange River are more distant from the Yoruba (0.028) and Zulu (0.043), but still more similar to these groups than to Himba (0.072). Overall, these patterns indicate that the Cederberg, Karoo, and Orange River populations share closer ancestry profiles with Yoruba, Luhya, and Zulu than with Himba. Similarly, ≠Khomani San NKA haplotype centroid is closest to the Yoruba (0.004) and moderately more distant to the Zulu (0.021) and Luhya (0.025), with increasing distance to Himba (0.095). In contrast, the Nama NKA haplotype centroid is moderately distant to all NKA reference groups: Yoruba (0.036), Zulu (0.051), Luhya (0.040), Himba (0.064).

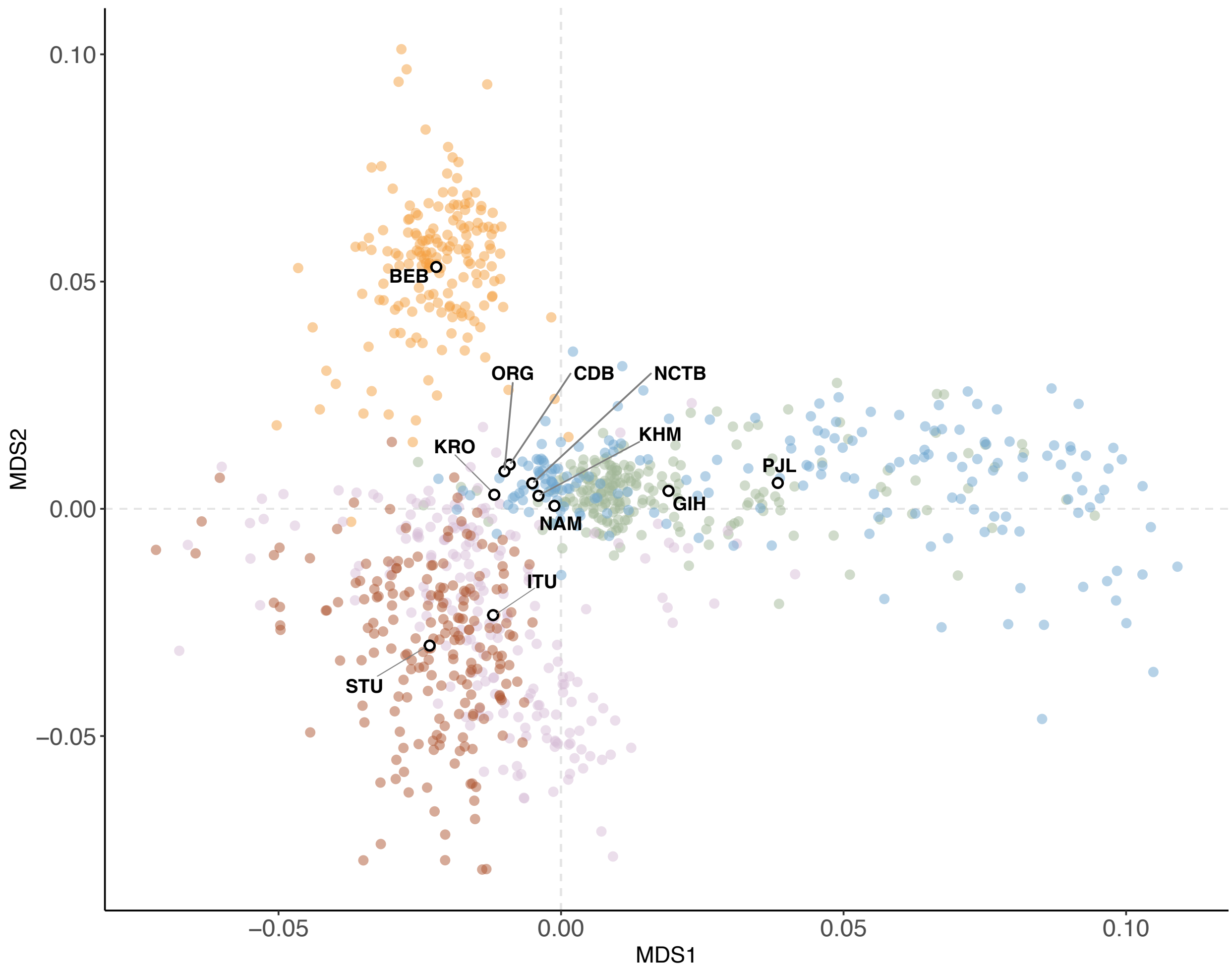


**Figure S21)** South Asian derived centroids plotted against reference populations. Query sample haplotypes are not plotted for clearer visualization of reference populations distributions.

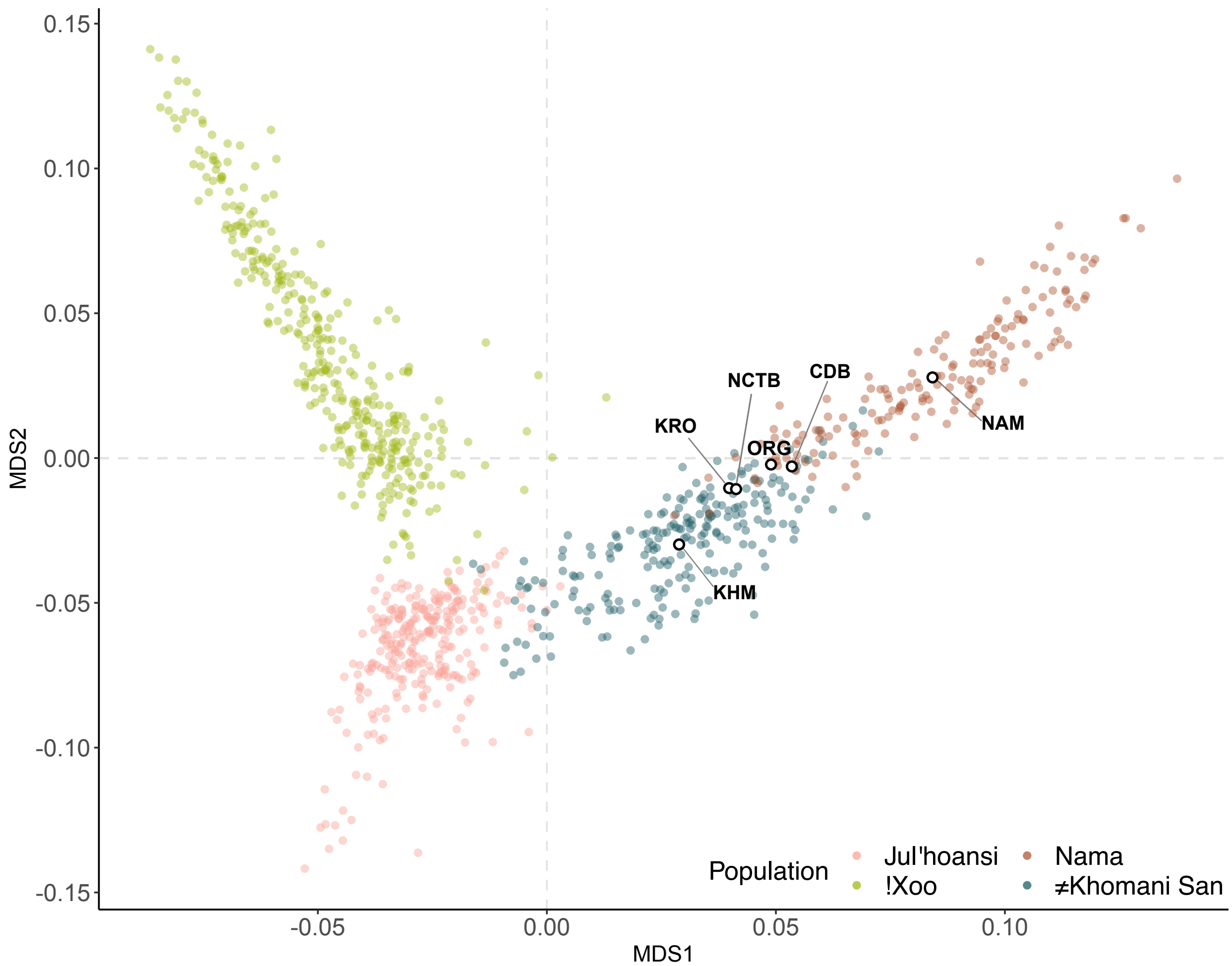


**Figure S22)** Khoe-San derived population centroids plotted against reference populations. Query sample haplotypes are not plotted for clearer visualization of reference populations distributions.

Inferring Botswanan Populations

We began by running an unsupervised ADMIXTURE inferring 2 to 7 population clusters (k) with the entirety of the Crawford et al. (2017) data (Supplemental Figure 1), along with the GBR, BEB, STU, GIH, KHV, IND, YRI, HMB, LWK, Zulu, Gumuz, Somali, Wolayta, Omoro, KHM, and Nama, and query populations. From there, we subset the Crawford et al. (2017) data for the 314 individuals with the highest Khoe-San ancestry proportion (Supplemental Figure 1A), as that is the reported number of San individuals sampled in the study,[75] and then re-ran ADMIXTURE k2 through k7 with those individuals (Supplemental Figure 1B).

We then filtered the Botswanan San, ≠Khomani, and Nama individuals with 85% or greater Khoe-San ancestry as inferred by ADMIXTURE k5 and plotted them on a PCA (Supplemental Figure 2A). We observe a previously reported cline among the Kalahari San populations, ≠Khomani, and Nama. The Botswanan San split into two groups, one of which anchored a corner of PC2. From there, we were able to infer the cluster of Botswanan San individuals driving PC1 likely have greater genetic variation relative to the other high Khoe-San populations. This leads us to infer that cluster of individuals to be the !Xoo, who were reported to have a higher population effective size relative to the Ju|'haonsi.[99]

We continued to our KHS ancestry-specific MDS. We add ≠Khomani, Nama, and Botswanan San individuals who did not meet the 85% Khoe-San ancestry cut-off into the query. We project those haplotypes, along with all first- and second-degree relatives in the data set, onto an MDS with the 85% and greater KHS reference. Projecting these individuals minimizes the effect relatives and admixture can have on dimensionality reduction. We use the mclust[100] R package to read in MDS2 data for the Botswanan San, the dimension where the Botswanan San split into two clusters. This package is designed for complex clustering and classification using a Gaussian finite mixture, meaning it assumes the data is made up of several Gaussian distributions. We use it to model MDS2 distribution with two clusters and identify the mean and standard deviation of both clusters (Supplemental Figure 2B-D). We filtered our model results to include only individuals with a probability of 0.9 or higher of belonging to a cluster. Upon examining samples across both clusters, we found no overlap between individuals assigned to each cluster. Most individuals who did not meet the ≥0.9 probability threshold are those positioned between the two cluster peaks, i.e., more than one standard deviation away from the modeled mean. These 10 individuals were labeled as BSAN_UNK (Botswanan San, Unknown). The individuals within the cluster positioned on the right side of MDS2 were labeled !Xoo, as they represent the samples contributing most to the genetic variation in MDS1. This classification leaves the remaining cluster of individuals to be identified as Ju|'hoansi (Supplemental Figure 3).

In order to refine the clustering of the assigned BSAN_UNK individuals, we performed a dimensionality reduction with uniform manifold approximation and projection (UMAP)[101] followed by genetic clustering with Hierarchical Density-Based Spatial Clustering of Applications with Noise implementation, HDBSCAN($\hat{\varepsilon}$).[102] We used as input the first 100 principal components (PCs) obtained with SmartPCA program from EIGENSOFT 7.2.1[27] and applied the same approach described in Diaz-Papkovich et al 2023.[103] For visualization purposes, UMAP was run with 25 number of nearest neighbors, a minimum distance of 0.3, and a dimension reduction of 2 components. For clustering, UMAP parameters included 50 number of neighbors, minimum distance of 0.01 and 5 number of components. Next, HDBSCAN($\hat{\varepsilon}$) was used to extract clusters using an $\hat{\varepsilon}$ of 0.3 and a minimum number of points in a cluster of 50.

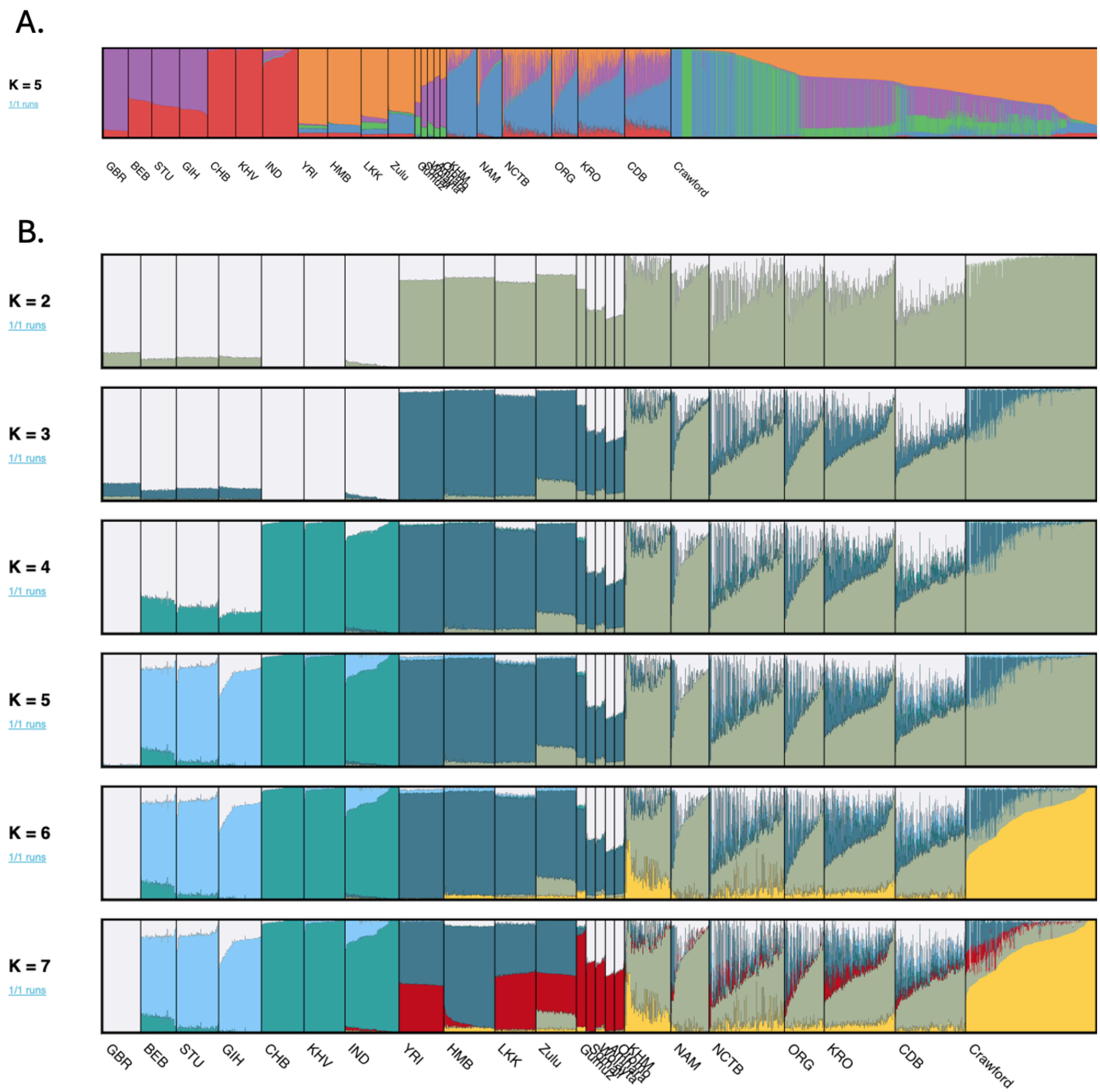


**Figure S23)** Identifying the Botswanan San using ADMIXTURE. **A)** ADMIXTURE at k5 with the entire Crawford et al. (2017) data. **B)** ADMIXTURE k2 through k7 with only 314 Botswanan San with the highest Khoe-San ancestry as inferred by figure A's results.

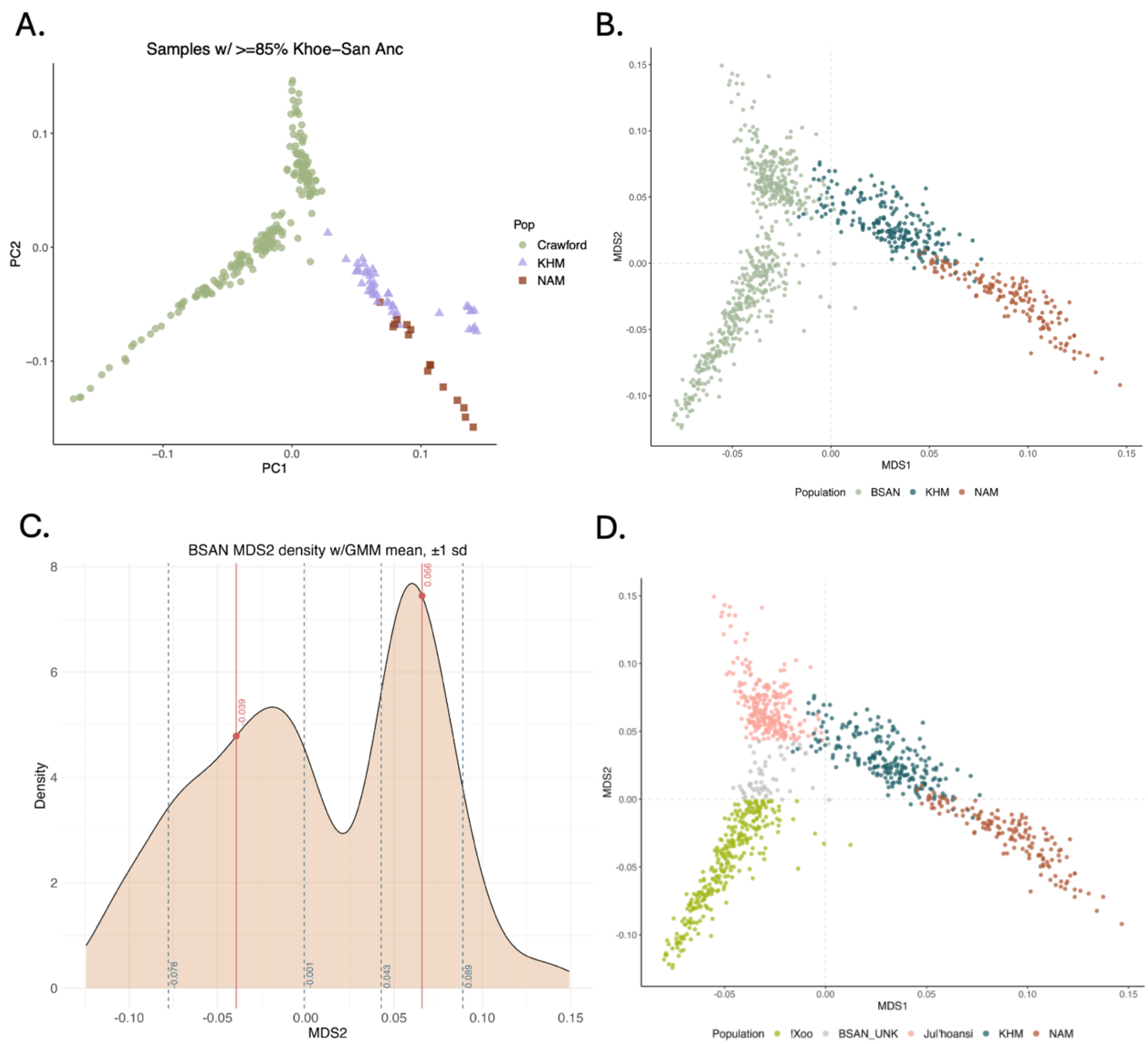


**Figure S24)** Differentiating Botswanan San Populations. **A)** Individuals with greater than 85% Khoe-San ancestry as inferred by ADMIXTURE k5 from Supplemental Figure 1A. **B)** Khoe-San (KHS) ancestry-specific multidimensional scaling (MDS) 1 and 2 of KHS reference populations. Individuals with less than 85% Khoe-San ancestry were added to the local ancestry inference query and projected into the MDS space, along with first- and second-degree relatives. **C)** mclust Gaussian mixture model identified mean and 1 standard deviation of MDS2 Botswanan San density. Red line specified averages while dashed blue lines highlight the 1 standard deviation cut offs. **D)** Cluster/population assignment as inferred by the Gaussian mixture model from mclust R package and previously published population effective size data[2].

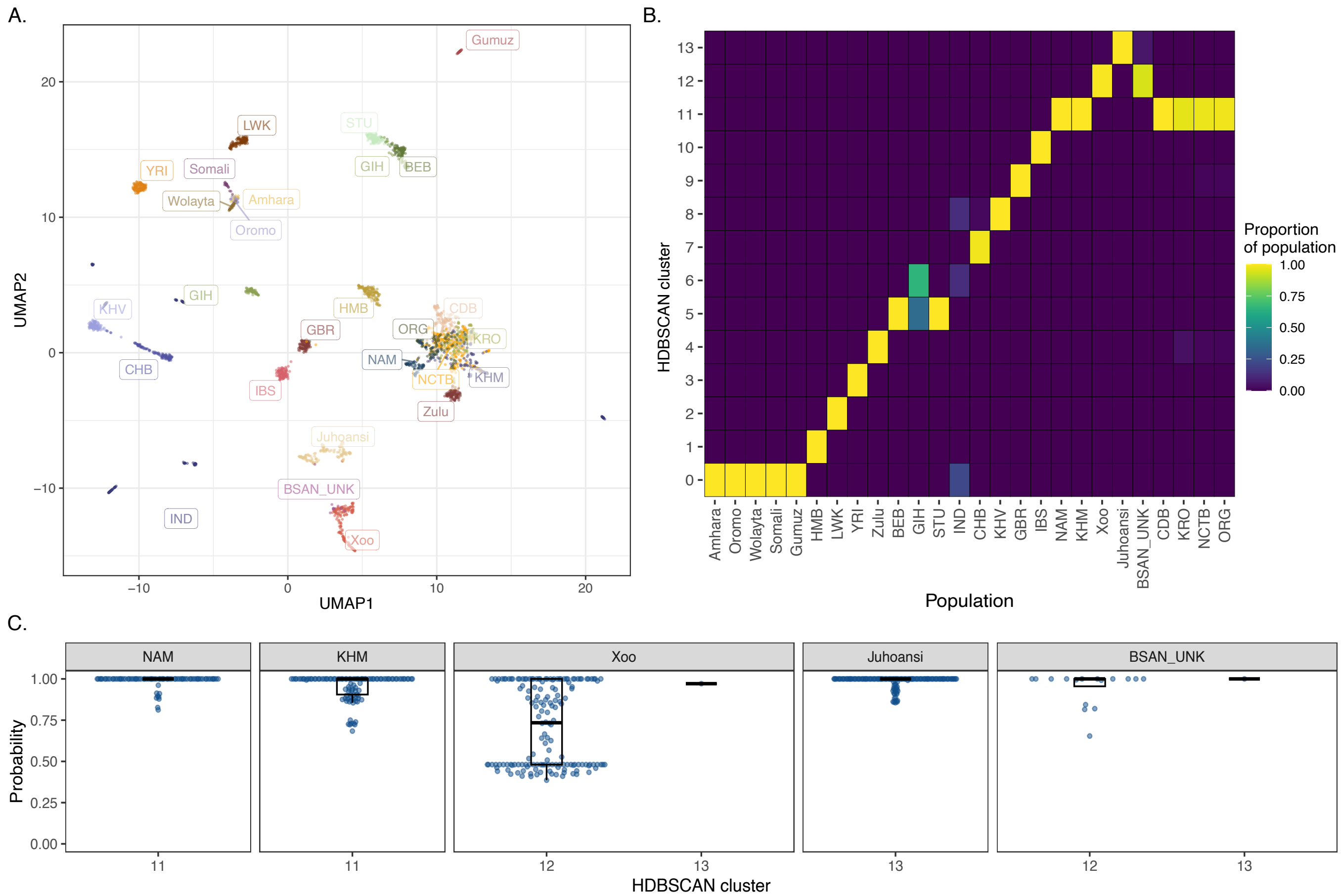


**Figure S25)** UMAP and HBDscan of reference and query populations **A.** UMAP generated with 25 nearest neighbors, and minimum distance of 0.3. Labels refer to population labels. **B.** Proportion of individuals of each population contained within a given cluster inferred from HDBscan algorithm (see Methods for further details on the parameters used). **C.** Distribution probabilities per individual to be in the cluster inferred in B for each Khoe-San reference population.

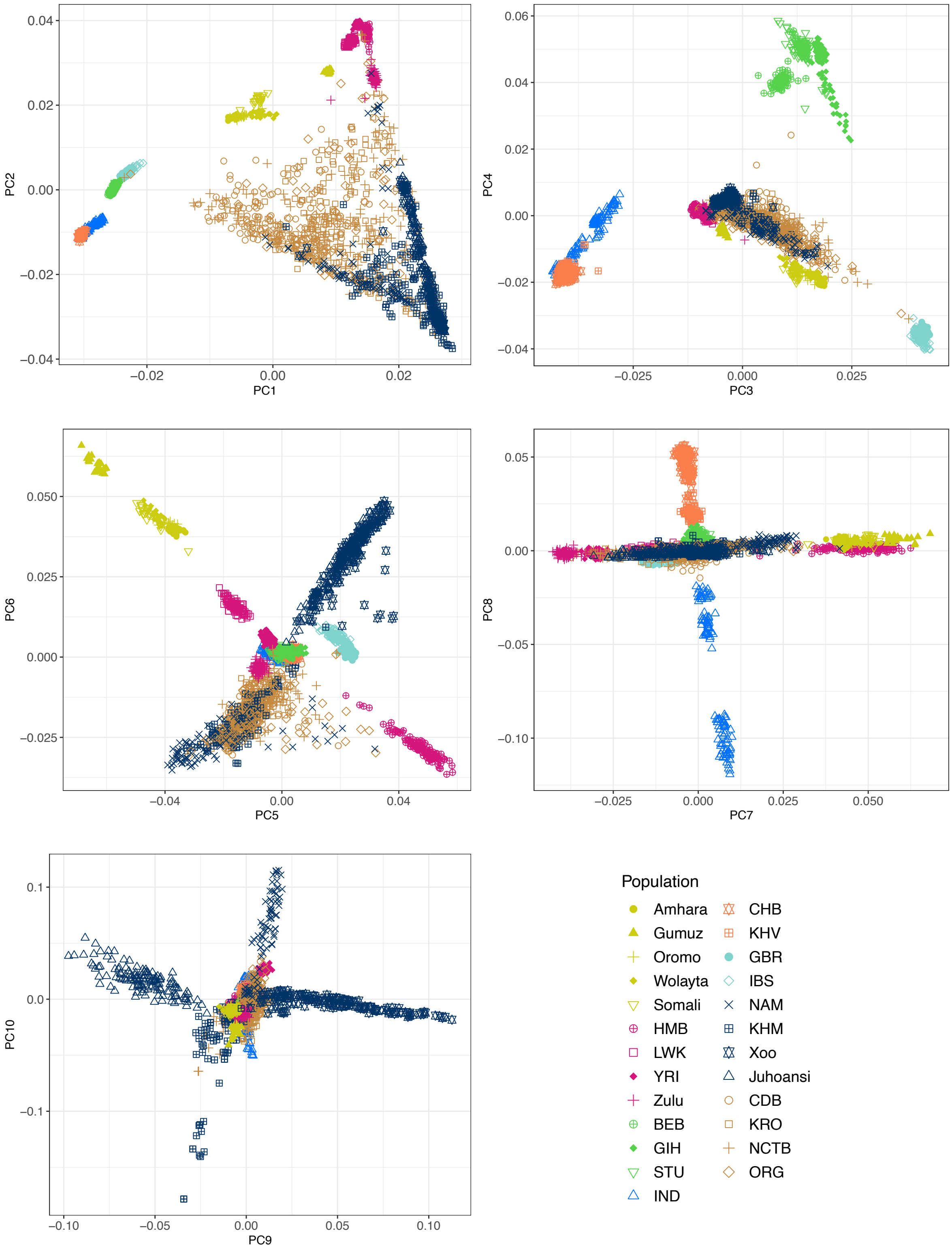


**Figure S26)** PC1 through PC10. PCs 1 through 10 were generated using smartPCA and plotted using ggplot2 in R. Colors roughly represent broader ancestry, while shapes represent population.

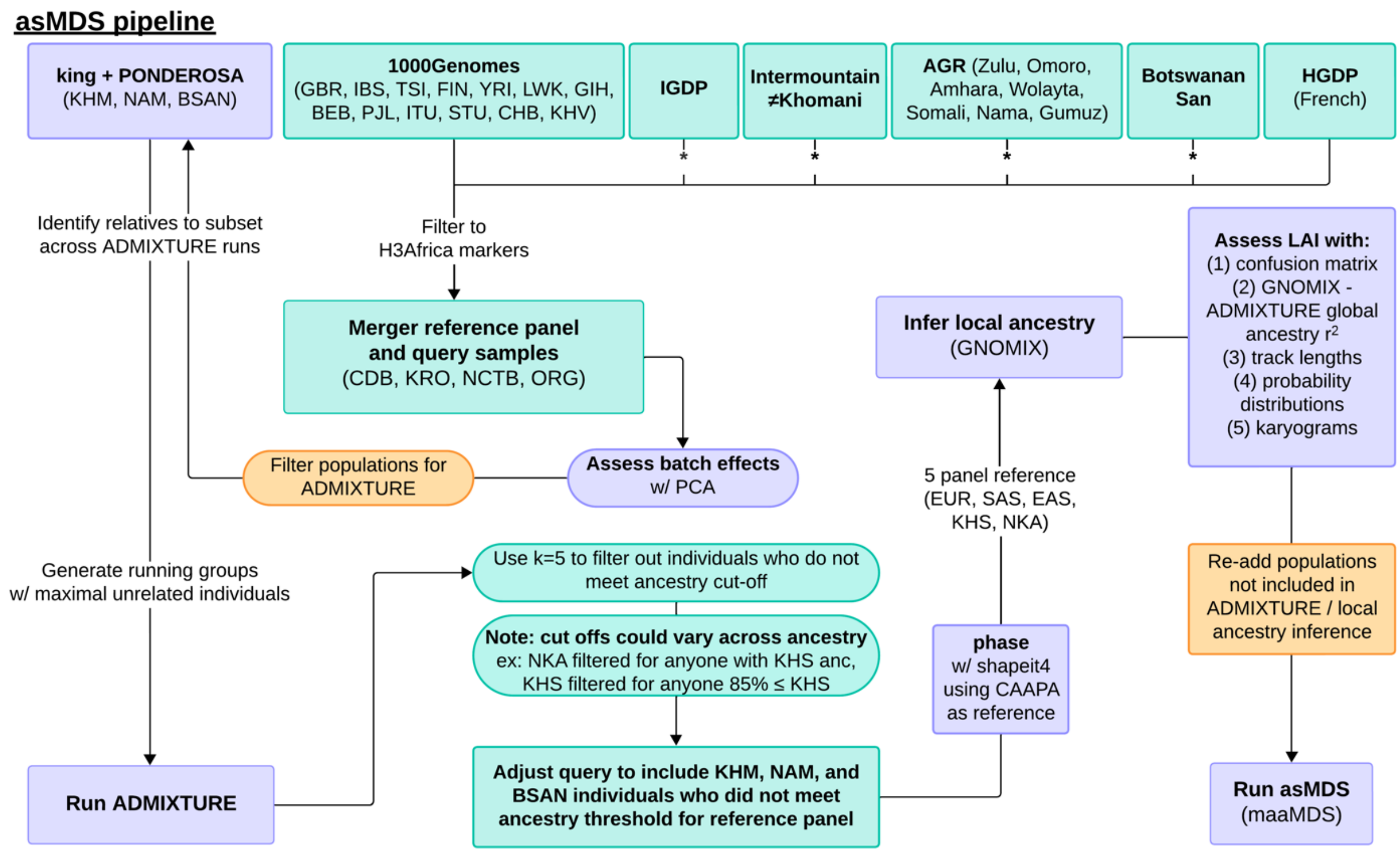


**Figure S27)** Local Ancestry and ancestry-specific MDS pipeline. Shapes in turquoise highlight steps taken regarding sample consolidation and filtering, while orange shapes are steps taken to filter or add entire populations. Shapes in purple major analyses executed throughout the pipeline. The * within lines represent whole genomes that were lifted from HG19 to HG38.

## REFERENCES


1. G. Breton, C. M. Schlebusch, M. Lombard, P. Sjödin, H. Soodyall, M. Jakobsson, Lactase persistence alleles reveal partial East African ancestry of Southern African khoe pastoralists. *Curr. Biol.* **24**, 852–858 (2014).

2. E. Macholdt, V. Lede, C. Barbieri, S. W. Mpoloka, H. Chen, M. Slatkin, B. Pakendorf, M. Stoneking, Tracing pastoralist migrations to Southern Africa with lactase persistence alleles. *Curr. Biol.* **24**, 875–879 (2014).

3. R. Elphick, H. Giliomee, *The Shaping of South African Society, 1652–1840* (Wesleyan Univ. Press, 2014).

4. N. Worden, "Slavery at the Cape history" in *Oxford Research Encyclopedia of African History*, T. Spear, Ed. (Oxford Academic, 2017).

5. R. F. Rohde, M. T. Hoffman, One hundred years of separation: The historical ecology of a South African "coloured reserve". *Africa* **78**, 189–222 (2007).

6. N. Penn, *The Forgotten Frontier: Colonist and Khoisan on the Cape's Northern Frontier in the 18th Century* (Ohio Univ. Press, 2005).

7. M. Adhikari, *Genocide on Settler Frontiers: When Hunter-Gatherers and Commercial Stock Farmers Clash* (University of Cape Town Press, 2015).

8. Statistics South Africa, Republic of South Africa, Census 2022 Population Count Results 10 October 2023 (2023); https://census.statssa.gov.za/assets/documents/2022/P03014_Census_2022_Statistical_Release.pdf.

9. M. Daya, L. van der Merwe, U. Galal, M. Möller, M. Salie, E. R. Chimusa, J. M. Galanter, P. D. van Helden, B. M. Henn, C. R. Gignoux, E. Hoal, A panel of ancestry informative markers for the complex five-way admixed South African Coloured population. *PLOS ONE* **8**, e82224 (2013).

10. I. Lankheet, R. Hammarén, L. X. A. Caballero, M. Larena, H. Malmström, C. Jolly, H. Soodyall, M. de Jongh, C. Schlebusch, Wide-scale geographical analysis of genetic ancestry in the South African coloured population. *BMC Biol.* **23**, 219 (2025).

11. E. de Wit, W. Delport, C. E. Rugamika, A. Meintjes, M. Möller, P. D. van Helden, C. Seoighe, E. G. Hoal, Genome-wide analysis of the structure of the South African coloured population in the Western Cape. *Hum. Genet.* **128**, 145–153 (2010).

12. L. Quintana-Murci, C. Harmant, H. Quach, O. Balanovsky, V. Zaporozhchenko, C. Bormans, P. D. van Helden, E. G. Hoal, D. M. Behar, Strong maternal khoisan contribution to the South African coloured population: A case of gender-biased admixture. *Am. J. Hum. Genet.* **86**, 611–620 (2010).

13. D. C. Petersen, O. Libiger, E. A. Tindall, R.-A. Hardie, L. I. Hannick, R. H. Glashoff, M. Mukerji, I. G. V. Consortium, P. Fernandez, W. Haacke, N. J. Schork, V. M. Hayes, Complex patterns of genomic admixture within Southern Africa. *PLOS Genet.* **9**, e1003309 (2013).

14. I. Lankheet, R. Hammarén, L. X. A. Caballero, M. Larena, H. Malmström, C. Jolly, H. Soodyall, M. de Jongh, C. Schlebusch, Wide-scale geographical analysis of genetic ancestry in the South African coloured population. bioRxiv 598620 [Preprint] (2024); www.biorxiv.org/content/10.1101/2024.06.13.598620v1.full.

15. B. M. Henn, C. R. Gignoux, M. Jobin, J. M. Granka, J. M. Macpherson, J. M. Kidd, L. Rodríguez-Botigué, S. Ramachandran, L. Hon, A. Brisbin, A. A. Lin, P. A. Underhill, D. Comas, K. K. Kidd, P. J. Norman, P. Parham, C. D. Bustamante, J. L. Mountain, M. W. Feldman, Hunter-gatherer genomic diversity suggests a southern African origin for modern humans. *Proc. Natl. Acad. Sci. U.S.A.* **108**, 5154–5162 (2011).

16. Y.-S. Chen, A. Olckers, T. G. Schurr, A. M. Kogelnik, K. Huoponen, D. C. Wallace, mtDNA variation in the South African Kung and Khwe—And their genetic relationships to other African populations. *Am. J. Hum. Genet.* **66**, 1362–1383 (2000).

17. P. A. Underhill, G. Passarino, A. A. Lin, P. Shen, M. M. Lahr, R. A. Foley, P. J. Oefner, L. L. Cavalli-Sforza, The phylogeography of Y chromosome binary haplotypes and the origins of modern human populations. *Ann. Hum. Genet.* **65**, 43–62 (2001).

18. G. D. Poznik, B. M. Henn, M.-C. Yee, E. Sliwerska, G. M. Euskirchen, A. A. Lin, M. Snyder, L. Quintana-Murci, J. M. Kidd, P. A. Underhill, C. D. Bustamante, Sequencing Y chromosomes resolves discrepancy in time to common ancestor of males versus females. *Science* **341**, 562–565 (2013).

19. C. Barbieri, A. Hübner, E. Macholdt, S. Ni, S. Lippold, R. Schröder, S. W. Mpoloka, J. Purps, L. Roewer, M. Stoneking, B. Pakendorf, Refining the Y chromosome phylogeny with southern African sequences. *Hum. Genet.* **135**, 541–553 (2016).

20. J. K. Pickrell, N. Patterson, C. Barbieri, F. Berthold, L. Gerlach, T. Güldemann, B. Kure, S. W. Mpoloka, H. Nakagawa, C. Naumann, M. Lipson, P.-R. Loh, J. Lachance, J. Mountain, C. D. Bustamante, B. Berger, S. A. Tishkoff, B. M. Henn, M. Stoneking, D. Reich, B. Pakendorf, The genetic prehistory of southern Africa. *Nat. Commun.* **3**, 1143 (2012).

21. C. M. Schlebusch, P. Skoglund, P. Sjödin, L. M. Gattepaille, D. Hernandez, F. Jay, S. Li, M. D. Jongh, A. Singleton, M. G. B. Blum, H. Soodyall, M. Jakobsson, Genomic variation in seven Khoe-San groups reveals adaptation and complex African history. *Science* **338**, 374–379 (2012).

22. A. Barnard, *Hunters and Herders of Southern Africa* (Cambridge Univ. Press, 2012).

23. M. Vicente, M. Jakobsson, P. Ebbesen, C. M. Schlebusch, Genetic affinities among southern Africa hunter-gatherers and the impact of admixing farmer and herder populations. *Mol. Biol. Evol.* **36**, 1849–1861 (2019).

24. N. Patterson, D. C. Petersen, R. E. van der Ross, H. Sudoyo, R. H. Glashoff, S. Marzuki, D. Reich, V. M. Hayes, Genetic structure of a unique admixed population: Implications for medical research. *Hum. Mol. Genet.* **19**, 411–419 (2010).

25. C. A. Fortes-Lima, C. Burgarella, R. Hammarén, A. Eriksson, M. Vicente, C. Jolly, A. Semo, H. Gunnink, S. Pacchiarotti, L. Mundeke, I. Matonda, J. K. Muluwa, P. Coutros, T. S. Nyambe, J.

C. Cikomola, V. Coetzee, M. de Castro, P. Ebbesen, J. Delanghe, M. Stoneking, L. Barham, M. Lombard, A. Meyer, M. Steyn, H. Malmström, J. Rocha, H. Soodyall, B. Pakendorf, K. Bostoen, C. M. Schlebusch, The genetic legacy of the expansion of Bantu-speaking peoples in Africa. *Nature* **625**, 540–547 (2024).

26. D. Sengupta, A. Choudhury, C. Fortes-Lima, S. Aron, G. Whitelaw, K. Bostoen, H. Gunnink, N. Chousou-Polydouri, P. Delius, S. Tollman, F. X. Gómez-Olivé, S. Norris, F. Mashinya, M. Alberts, A.-G. Study, H. Consortium, S. Hazelhurst, C. M. Schlebusch, M. Ramsay, Genetic substructure and complex demographic history of South African Bantu speakers. *Nat. Commun.* **12**, 2080 (2021).

27. N. Patterson, A. L. Price, D. Reich, Population structure and eigenanalysis. *PLOS Genet.* **2**, e190 (2006).

28. D. H. Alexander, J. Novembre, K. Lange, Fast model-based estimation of ancestry in unrelated individuals. *Genome Res.* **19**, 1655–1664 (2009).

29. N. A. Swinford, S. P. Prall, S. Gopalan, C. M. Williams, J. Sheehama, B. A. Scelza, B. M. Henn, Increased homozygosity due to endogamy results in fitness consequences in a human population. *Proc. Natl. Acad. Sci. U.S.A.* **120**, e2309552120 (2023).

30. L. H. Robbins, A. C. Campbell, M. L. Murphy, G. A. Brook, F. Liang, S. A. Skaggs, P. Srivastava, A. A. Mabuse, S. Badenhorst, Recent archaeological research at Toteng, Botswana: Early domesticated livestock in the Kalahari. *J. Afr. Archaeol.* **6**, 131–149 (2008).

31. M. Vicente, I. Lankheet, T. Russell, N. Hollfelder, V. Coetzee, H. Soodyall, M. D. Jongh, C. M. Schlebusch, Male-biased migration from East Africa introduced pastoralism into southern Africa. *BMC Biol.* **19**, 259 (2021).

32. A. G. Ioannidis, J. Blanco-Portillo, K. Sandoval, E. Hagelberg, J. F. Miquel-Poblete, J. V. Moreno-Mayar, J. E. Rodríguez-Rodríguez, C. D. Quinto-Cortés, K. Auckland, T. Parks, K. Robson, A. V. S. Hill, M. C. Avila-Arcos, A. Sockell, J. R. Homburger, G. L. Wojcik, K. C. Barnes, L. Herrera, S. Berríos, M. Acuña, E. Llop, C. Eng, S. Huntsman, E. G. Burchard, C. R.

Gignoux, L. Cifuentes, R. A. Verdugo, M. Moraga, A. J. Mentzer, C. D. Bustamante, A. Moreno-Estrada, Native American gene flow into Polynesia predating Easter Island settlement. *Nature* **583**, 572–577 (2020).

33. H. Hilmarsson, A. S. Kumar, R. Rastogi, C. D. Bustamante, D. M. Montserrat, A. G. Ioannidis, High resolution ancestry deconvolution for next generation genomic data. bioRxiv 460980 [Preprint] (2021); www.biorxiv.org/content/10.1101/2021.09.19.460980v1.

34. B. Heine, D. Nurse, K. Williamson, R. Blench, L. M. Bender, R. J. Hayward, T. Güldemann, R. Vossen, G. N. Clements, G. J. Dimmendaal, J. R. Watters, D. Creissels, P. Newman, C. Ehret, H. Ekkehard Wolff, *African Languages* (Cambridge Univ. Press, 2000).

35. R. Blench, *Archaeology, Language, and the African Past* (AltaMira Press, 2006).

36. C. M. Schlebusch, M. Jakobsson, Tales of human migration, admixture, and selection in Africa. *Annu. Rev. Genomics Hum. Genet.* **19**, 405–428 (2018).

37. F. Montinaro, G. B. J. Busby, M. Gonzalez-Santos, O. Oosthuitzen, E. Oosthuitzen, P. Anagnostou, G. Destro-Bisol, V. L. Pascali, C. Capelli, Complex ancient genetic structure and cultural transitions in Southern African populations. *Genetics* **205**, 303–316 (2017).

38. R. B. Beck, *The History of South Africa* (ABC-CLIO, 2013), 10.5040/9798400665363.

39. G. Hudjashov, T. M. Karafet, D. J. Lawson, S. Downey, O. Savina, H. Sudoyo, J. S. Lansing, M. F. Hammer, M. P. Cox, Complex patterns of admixture across the Indonesian archipelago. *Mol. Biol. Evol.* **34**, 2439–2452 (2017).

40. L. Mbeki, M. van Rossum, Private slave trade in the Dutch Indian Ocean world: A study into the networks and backgrounds of the slavers and the enslaved in South Asia and South Africa. *Slavery Abolit.* **38**, 95–116 (2017).

41. R. M. Byrnes, *South Africa: A Country Study* (Federal Research Division, Library of Congress, 1997).

42. H. Al-Asadi, D. Petkova, M. Stephens, J. Novembre, Estimating recent migration and population-size surfaces. *PLOS Genet.* **15**, e1007908 (2019).

43. E. A. Thompson, Identity by descent: Variation in meiosis, across genomes, and in populations. *Genetics* **194**, 301–326 (2013).

44. N. Hollfelder, G. Breton, P. Sjödin, M. Jakobsson, The deep population history in Africa. *Hum. Mol. Genet.* **30**, ddab005 (2021).

45. A. Choudhury, S. Aron, D. Sengupta, S. Hazelhurst, M. Ramsay, African genetic diversity provides novel insights into evolutionary history and local adaptations. *Hum. Mol. Genet.* **27**, R209–R218 (2018).

46. A. Choudhury, M. Ramsay, S. Hazelhurst, S. Aron, S. Bardien, G. Botha, E. R. Chimusa, A. Christoffels, J. Gamieldien, M. J. Sefid-Dashti, F. Joubert, A. Meintjes, N. Mulder, R. Ramesar, J. Rees, K. Scholtz, D. Sengupta, H. Soodyall, P. Venter, L. Warnich, M. S. Pepper, Whole-genome sequencing for an enhanced understanding of genetic variation among South Africans. *Nat. Commun.* **8**, 2062 (2017).

47. E. R. Chimusa, M. Daya, M. Möller, R. Ramesar, B. M. Henn, P. D. van Helden, N. J. Mulder, E. G. Hoal, Determining ancestry proportions in complex admixture scenarios in South Africa using a novel proxy ancestry selection method. *PLOS ONE* **8**, e73971 (2013).

48. N. Hollfelder, J. C. Erasmus, R. Hammaren, M. Vicente, M. Jakobsson, J. M. Greeff, C. M. Schlebusch, Patterns of African and Asian admixture in the Afrikaner population of South Africa. *BMC Biol.* **18**, 16 (2020).

49. P. Harries, Mozambique Island, Cape Town and the organisation of the slave trade in the South-West Indian Ocean, c.1797–1807. *J. South. Afr. Stud.* **42**, 409–427 (2016).

50. C. Uren, M. Kim, A. R. Martin, D. Bobo, C. R. Gignoux, P. D. van Helden, M. Möller, E. G. Hoal, B. M. Henn, Fine-scale human population structure in Southern Africa reflects ecogeographic boundaries. *Genetics* **204**, 303–314 (2016).

51. D. Philip, “The Cape Huguenots and their legacy in apartheid South Africa” in *Memory and Identity: The Huguenots in France and the Atlantic Diaspora*, B. V. Ruymbeke, R. J. Sparks, Eds. (Columbia, University of South Carolina Press, 2003), pp. 285–309.

52. W. Rees, The Oxford History of South Africa. Volume I, South Africa to 1870. Edited by Monica Wilson and Leonard Thompson. (New York: Oxford University Press. 1969. Pp. xviii, 502. $8.00.). *Am. Hist. Rev.* **75**, 899–900 (1970).

53. N. Etherington, The Great Treks: The Transformation of Southern Africa, 1815-1854. *J. South. Afr. Stud.* **28**, 651–660 (2002).

54. L. M. Kootker, L. Mbeki, A. G. Morris, H. Kars, G. R. Davies, Dynamics of Indian ocean slavery revealed through isotopic data from the colonial era cobern street burial site, Cape Town, South Africa (1750–1827). *PLOS ONE* **11**, e0157750 (2016).

55. M. Vink, “The world’s oldest trade”: Dutch slavery and slave trade in the Indian ocean in the seventeenth century. *J. World Hist.* **14**, 131–177 (2003).

56. N. Worden, “Indian Ocean slavery and its demise in the Cape Colony” in *Abolition and its aftermath in the Indian Ocean Africa and Asia*, G. Campbell, Ed. (Routledge, 2005), pp. 29–49.

57. I. D. du Plessis, *The Cape Malays* (Maskew Miller, Zambia, 1947).

58. L. Guelke, Frontier settlement in early Dutch South Africa. *Ann. Assoc. Am. Geogr.* **66**, 25–42 (1976).

59. L. J. Mitchell, Traces in the landscape: Hunters, herders and farmers on the cedarberg frontier, South Africa, 1725-95. *J. Afr. Hist.* **43**, 431–450 (2002).

60. A. W. Reynolds, M. N. Grote, J. W. Myrick, D. R. Al-Hindi, R. L. Siford, M. Mastoras, M. Möller, B. M. Henn, Persistence of matrilocal postmarital residence across multiple generations in Southern Africa. *Hum. Nat.* **34**, 295–323 (2023).

61. A. Desai, G. Vahed, “Between splitting and lumping: The naming and framing of Coloureds and Indians before apartheid” in *The Oxford Handbook of South African History* (Oxford Academic, 2022); 10.1093/oxfordhb/9780190921767.013.28.

62. C. M. Schlebusch, H. Soodyall, Extensive population structure in San, Khoe, and mixed ancestry populations from Southern Africa revealed by 44 short 5-SNP haplotypes. *Hum. Biol.* **84**, 695–724 (2012).

63. C. Barbieri, M. Vicente, J. Rocha, S. W. Mpoloka, M. Stoneking, B. Pakendorf, Ancient substructure in early mtDNA lineages of southern Africa. *Am. J. Hum. Genet.* **92**, 285–92 (2013).

64. C. Barbieri, T. Güldemann, C. Naumann, L. Gerlach, F. Berthold, H. Nakagawa, S. W. Mpoloka, M. Stoneking, B. Pakendorf, Unraveling the complex maternal history of Southern African Khoisan populations. *Am. J. Phys. Anthropol.* **153**, 435–448 (2013).

65. S. Gopalan, R. E. W. Berl, J. W. Myrick, Z. H. Garfield, A. W. Reynolds, B. K. Bafens, G. Belbin, M. Mastoras, C. Williams, M. Daya, A. N. Negash, M. W. Feldman, B. S. Hewlett, B. M. Henn, Hunter-gatherer genomes reveal diverse demographic trajectories during the rise of farming in Eastern Africa. *Curr. Biol.* **32**, 1852–1860.e5 (2022).

66. J. N. Fenner, Cross-cultural estimation of the human generation interval for use in genetics-based population divergence studies. *Am. J. Phys. Anthr.* **128**, 415–423 (2005).

67. M. Barry, Now another thing must happen: Richtersveldand the dilemmas of land reform in post-apartheid South Africa. *S. Afr. J. Hum. Rights* **20**, 355–382 (2004).

68. R. Ross, *Cape of Torments: Slavery and Resistance in South Africa* (Routledge & Kegan Paul, 1983).

69. W. Dooling, The origins and aftermath of the Cape colony’s “Hottentot Code” of 1809. *Kronos* **31**, 50–61 (2005).

70. O. P. Oyageshio, J. W. Myrick, J. Saayman, L. van der Westhuizen, D. R. Al-Hindi, A. W. Reynolds, N. Zaitlen, E. G. Hoal, C. Uren, M. Möller, B. M. Henn, Strong effect of demographic changes on tuberculosis susceptibility in South Africa. *PLOS Glob. Public Heal.* **4**, e0002643 (2024).

71. D. Gurdasani, T. Carstensen, F. Tekola-Ayele, L. Pagani, I. Tachmazidou, K. Hatzikotoulas, S. Karthikeyan, L. Iles, M. O. Pollard, A. Choudhury, G. R. S. Ritchie, Y. Xue, J. Asimit, R. N. Nsubuga, E. H. Young, C. Pomilla, K. Kivinen, K. Rockett, A. Kamali, A. P. Doumatey, G. Asiki, J. Seeley, F. Sisay-Joof, M. Jallow, S. Tollman, E. Mekonnen, R. Ekong, T. Oljira, N. Bradman, K. Bojang, M. Ramsay, A. Adeyemo, E. Bekele, A. Motala, S. A. Norris, F. Pirie, P. Kaleebu, D. Kwiatkowski, C. Tyler-Smith, C. Rotimi, E. Zeggini, M. S. Sandhu, The African Genome Variation Project shapes medical genetics in Africa. *Nature* **517**, 327–332 (2015).

72. H. M. Cann, C. de Toma, L. Cazes, M.-F. Legrand, V. Morel, L. Piouffre, J. Bodmer, W. F. Bodmer, B. Bonne-Tamir, A. Cambon-Thomsen, Z. Chen, J. Chu, C. Carcassi, L. Contu, R. Du, L. Excoffier, G. B. Ferrara, J. S. Friedlaender, H. Groot, D. Gurwitz, T. Jenkins, R. J. Herrera, X. Huang, J. Kidd, K. K. Kidd, A. Langaney, A. A. Lin, S. Q. Mehdi, P. Parham, A. Piazza, M. P. Pistillo, Y. Qian, Q. Shu, J. Xu, S. Zhu, J. L. Weber, H. T. Greely, M. W. Feldman, G. Thomas, J. Dausset, L. L. Cavalli-Sforza, A human genome diversity cell line panel. *Science* **296**, 261–262 (2002).

73. D. M. Vespasiani, G. S. Jacobs, L. E. Cook, N. Brucato, M. Leavesley, C. Kinipi, F.-X. Ricaut, M. P. Cox, I. G. Romero, Denisovan introgression has shaped the immune system of present-day Papuans. *PLOS Genet.* **18**, e1010470 (2022).

74. 1000 Genomes Project Consortium, A. Auton, L. D. Brooks, R. M. Durbin, E. P. Garrison, H. M. Kang, J. O. Korbel, J. L. Marchini, S. McCarthy, G. A. McVean, A global reference for human genetic variation. *Nature* **526**, 68–74 (2015).

75. A. R. Martin, M. Lin, J. M. Granka, J. W. Myrick, X. Liu, A. Sockell, E. G. Atkinson, C. J. Werely, M. Möller, M. S. Sandhu, D. M. Kingsley, E. G. Hoal, X. Liu, M. J. Daly, M. W. Feldman, C. R. Gignoux, C. D. Bustamante, B. M. Henn, An unexpectedly complex architecture for skin pigmentation in Africans. *Cell* **171**, 1340–1353.e14 (2017).

76. N. G. Crawford, D. E. Kelly, M. E. B. Hansen, M. H. Beltrame, S. Fan, S. L. Bowman, E. Jewett, A. Ranciaro, S. Thompson, Y. Lo, S. P. Pfeifer, J. D. Jensen, M. C. Campbell, W. Beggs, F. Hormozdiari, S. W. Mpoloka, G. G. Mokone, T. Nyambo, D. W. Meskel, G. Belay, J. Haut, N. C. S. Program, H. Rothschild, L. Zon, Y. Zhou, M. A. Kovacs, M. Xu, T. Zhang, K. Bishop, J. Sinclair, C. Rivas, E. Elliot, J. Choi, S. A. Li, B. Hicks, S. Burgess, C. Abnet, D. E. Watkins-Chow, E. Oceana, Y. S. Song, E. Eskin, K. M. Brown, M. S. Marks, S. K. Loftus, W. J. Pavan, M. Yeager, S. Chanock, S. A. Tishkoff, Loci associated with skin pigmentation identified in African populations. *Science* **358**, eaan8433 (2017).

77. B. A. Scelza, S. P. Prall, N. Swinford, S. Gopalan, E. G. Atkinson, R. McElreath, J. Sheehama, B. M. Henn, High rate of extrapair paternity in a human population demonstrates diversity in human reproductive strategies. *Sci. Adv.* **6**, eaay6195 (2020).

78. G. Genovese, N. B. Rockweiler, B. R. Gorman, T. B. Bigdeli, M. T. Pato, C. N. Pato, K. Ichihara, S. A. McCarroll, BCFtools/liftover: An accurate and comprehensive tool to convert genetic variants across genome assemblies. *Bioinformatics* **40**, btae038 (2024).

79. P. Danecek, J. K. Bonfield, J. Liddle, J. Marshall, V. Ohan, M. O. Pollard, A. Whitwham, T. Keane, S. A. McCarthy, R. M. Davies, H. Li, Twelve years of SAMtools and BCFtools. *GigaScience* **10**, giab008 (2021).

80. O. Delaneau, J.-F. Zagury, M. R. Robinson, J. L. Marchini, E. T. Dermitzakis, Accurate, scalable and integrative haplotype estimation. *Nat. Commun.* **10**, 5436 (2019).

81. R. A. Mathias, M. A. Taub, C. R. Gignoux, W. Fu, S. Musharoff, T. D. O'Connor, C. Vergara, D. G. Torgerson, M. Pino-Yanes, S. S. Shringarpure, L. Huang, N. Rafaels, M. P. Boorgula, H. R. Johnston, V. E. Ortega, A. M. Levin, W. Song, R. Torres, B. Padhukasahasram, C. Eng, D.-A. Mejia-Mejia, T. Ferguson, Z. S. Qin, A. F. Scott, M. Yazdanbakhsh, J. G. Wilson, J. Marrugo, L. A. Lange, R. Kumar, P. C. Avila, L. K. Williams, H. Watson, L. B. Ware, C. Olopade, O. Olopade, R. Oliveira, C. Ober, D. L. Nicolae, D. Meyers, A. Mayorga, J. Knight-Madden, T. Hartert, N. N. Hansel, M. G. Foreman, J. G. Ford, M. U. Faruque, G. M. Dunston, L. Caraballo, E. G. Burchard, E. Bleecker, M. I. Araujo, E. F. Herrera-Paz, K. Gietzen, W. E. Grus, M. Bamshad, C. D. Bustamante, E. E. Kenny, R. D. Hernandez, T. H. Beaty, I. Ruczinski, J. Akey,

CAAPA, K. C. Barnes, A continuum of admixture in the Western Hemisphere revealed by the African Diaspora genome. *Nat. Commun.* **7**, 12522 (2016).

82. C. M. Williams, B. A. Scelza, S. D. Slack, N. Font-Porterias, D. R. Al-Hindi, R. A. Mathias, H. Watson, K. C. Barnes, E. Lange, R. K. Johnson, C. R. Gignoux, S. Ramachandran, B. M. Henn, A rapid accurate approach to inferring pedigrees in endogamous populations. *Genetics*, **230**, iyaf094 (2025).

83. M. Caballero, D. N. Seidman, Y. Qiao, J. Sannerud, T. D. Dyer, D. M. Lehman, J. E. Curran, R. Duggirala, J. Blangero, S. Carmi, A. L. Williams, Crossover interference and sex-specific genetic maps shape identical by descent sharing in close relatives. *PLOS Genet.* **15**, e1007979 (2019).

84. C. L. Campbell, N. A. Furlotte, N. Eriksson, D. Hinds, A. Auton, Escape from crossover interference increases with maternal age. *Nat. Commun.* **6**, 6260 (2015).

85. C. Bhérer, C. L. Campbell, A. Auton, Refined genetic maps reveal sexual dimorphism in human meiotic recombination at multiple scales. *Nat. Commun.* **8**, 14994 (2017).

86. W. A. Freyman, K. F. McManus, S. S. Shringarpure, E. M. Jewett, K. Bryc, 23 and Me Research Team, A. Auton, Fast and robust identity-by-descent inference with the templated positional Burrows–Wheeler transform. *Mol. Biol. Evol.* **38**, 2131–2151 (2020).

87. A. Manichaikul, J. C. Mychaleckyj, S. S. Rich, K. Daly, M. Sale, W.-M. Chen, Robust relationship inference in genome-wide association studies. *Bioinformatics* **26**, 2867–2873 (2010).

88. S. Purcell, B. Neale, K. Todd-Brown, L. Thomas, M. A. R. Ferreira, D. Bender, J. Maller, P. Sklar, P. I. W. de Bakker, M. J. Daly, P. C. Sham, PLINK: A tool set for whole-genome association and population-based linkage analyses. *Am. J. Hum. Genet.* **81**, 559–575 (2007).

89. A. A. Behr, K. Z. Liu, G. Liu-Fang, P. Nakka, S. Ramachandran, pong: Fast analysis and visualization of latent clusters in population genetic data. *Bioinformatics* **32**, 2817–2823 (2016).

90. B. M. Henn, L. R. Botigué, S. Gravel, W. Wang, A. Brisbin, J. K. Byrnes, K. Fadhlaoui-Zid, P. A. Zalloua, A. Moreno-Estrada, J. Bertranpetit, C. D. Bustamante, D. Comas, Genomic ancestry of North Africans supports back-to-Africa migrations. *PLOS Genet.* **8**, e1002397 (2012).

91. H. Wickham, ggplot2. *Wiley Interdiscip. Rev. Comput. Stat.* **3**, 180–185 (2011).

92. E. Pless, A. M. Eckburg, B. M. Henn, Predicting environmental and ecological drivers of human population structure. *Mol. Biol. Evol.* **40**, msad094 (2023).

93. C. M. Williams, B. A. Scelza, M. Daya, E. M. Lange, C. R. Gignoux, B. M. Henn, A rapid, accurate approach to inferring pedigrees in endogamous populations. bioRxiv 965376 [Preprint] (2020); www.biorxiv.org/content/10.1101/2020.02.25.965376v2.

94. Y. Zhou, S. R. Browning, B. L. Browning, A fast and simple method for detecting identity-by-descent segments in large-scale data. *Am. J. Hum. Genet.* **106**, 426–437 (2020).

95. D. N. Karger, O. Conrad, J. Böhner, T. Kawohl, H. Kreft, R. W. Soria-Auza, N. E. Zimmermann, H. P. Linder, M. Kessler, Climatologies at high resolution for the earth's land surface areas. *Sci. Data* **4**, 170122 (2017).

96. G. Amatulli, D. McInerney, T. Sethi, P. Strobl, S. Domisch, Geomorpho90m, empirical evaluation and accuracy assessment of global high-resolution geomorphometric layers. *Sci. Data* **7**, 162 (2020).

97. D. Yamazaki, D. Ikeshima, R. Tawatari, T. Yamaguchi, F. O'Loughlin, J. C. Neal, C. C. Sampson, S. Kanae, P. D. Bates, A high-accuracy map of global terrain elevations. *Geophys. Res. Lett.* **44**, 5844–5853 (2017).

98. J. N. Saada, G. Kalantzis, D. Shyr, F. Cooper, M. Robinson, A. Gusev, P. F. Palamara, Identity-by-descent detection across 487,409 British samples reveals fine scale population structure and ultra-rare variant associations. *Nat. Commun.* **11**, 6130 (2020).

99. S. Fan, J.P. Spence, Y. Feng, M.E.B. Hansen, J. Terhorst, M.H. Beltrame, A. Ranciaro, J. Hirbo, W. Beggs, N. Thomas, T. Nyambo, S. W. Mpoloka, G. G. Mokone, A. K. Njamnshi, C.

Fokunang, D. W. Meskel, G. Belay, Y. S. Song, S. A. Tishkoff, Whole-genome sequencing reveals a complex African population demographic history and signatures of local adaptation. *Cell* **186**, 923–939.e14 (2023).

100. L. Scrucca, M. Fop, T.B. Murphy, A.E. Raftery, mclust 5: Clustering, classification and density estimation using gaussian finite mixture models. *R J.* **8**, 289–317 (2016).

101. L. McInnes, J. Healy, N. Saul, L. Großberger, UMAP: Uniform manifold approximation and projection. *J. Open Source Softw.* **3**, 861 (2018).

102. C. Malzer, M. Baum, "A hybrid approach to hierarchical density-based cluster selection," in *2020 IEEE International Conference on Multisensor Fusion and Integration for Intelligent Systems (MFI)* (IEEE, 2020), pp. 223–228.

103. A. Diaz-Papkovich, S. Zabad, H. Snell, C. Ben-Eghan, L. Anderson-Trocmé, G. Femerling, V. Nathan, J. Patel, S. Gravel, Topological stratification of continuous genetic variation in large biobanks. *PLOS Genet.* **22**, e1012068 (2026).

104. D. Reich, K. Thangaraj, N. Patterson, A. L. Price, L. Singh, Reconstructing Indian population history. *Nature* **461**, 489–494 (2009).

105. M. Metspalu, M. Mondal, G. Chaubey, The genetic makings of South Asia. *Curr. Opin. Genet. Dev.* **53**, 128–133 (2018).

106. E. Kerdoncuff, L. Skov, N. Patterson, J. Banerjee, P. Khobragade, S. S. Chakrabarti, A. Chakrawarty, P. Chatterjee, M. Dhar, M. Gupta, J. P. John, P. A. Koul, S. S. Lehl, R. R. Mohanty, M. Padmaja, A. Perianayagam, C. Rajguru, L. Sankhe, A. Talukdar, M. Varghese, S. R. Yadati, W. Zhao, Y. Y. Leung, G. D. Schellenberg, Y. Z. Wang, J. A. Smith, S. Dey, A. Ganna, A. B. Dey, S. L. R. Kardia, J. Lee, P. Moorjani, 50,000 years of evolutionary history of India: Impact on health and disease variation. *Cell* **188**, 3389–3404.e6 (2025).

107. P. Moorjani, K. Thangaraj, N. Patterson, M. Lipson, P.-R. Loh, P. Govindaraj, B. Berger, D. Reich, L. Singh, Genetic evidence for recent population mixture in India. *Am. J. Hum. Genet.* **93**, 422–438 (2013).
